# Assessment of network module identification across complex diseases

**DOI:** 10.1038/s41592-019-0509-5

**Published:** 2019-08-30

**Authors:** Sarvenaz Choobdar, Mehmet E. Ahsen, Jake Crawford, Mattia Tomasoni, Tao Fang, David Lamparter, Junyuan Lin, Benjamin Hescott, Xiaozhe Hu, Johnathan Mercer, Ted Natoli, Rajiv Narayan, The DREAM Module Identification Challenge Consortium, Fabian Aicheler, Nicola Amoroso, Alex Arenas, Karthik Azhagesan, Aaron Baker, Michael Banf, Serafim Batzoglou, Anaïs Baudot, Roberto Bellotti, Sven Bergmann, Keith A. Boroevich, Christine Brun, Stanley Cai, Michael Caldera, Alberto Calderone, Gianni Cesareni, Weiqi Chen, Christine Chichester, Sarvenaz Choobdar, Lenore Cowen, Jake Crawford, Hongzhu Cui, Phuong Dao, Manlio De Domenico, Andi Dhroso, Gilles Didier, Mathew Divine, Antonio del Sol, Tao Fang, Xuyang Feng, Jose C. Flores-Canales, Santo Fortunato, Anthony Gitter, Anna Gorska, Yuanfang Guan, Alain Guénoche, Sergio Gómez, Hatem Hamza, András Hartmann, Shan He, Anton Heijs, Julian Heinrich, Benjamin Hescott, Xiaozhe Hu, Ying Hu, Xiaoqing Huang, V. Keith Hughitt, Minji Jeon, Lucas Jeub, Nathan T. Johnson, Keehyoung Joo, InSuk Joung, Sascha Jung, Susana G. Kalko, Piotr J. Kamola, Jaewoo Kang, Benjapun Kaveelerdpotjana, Minjun Kim, Yoo-Ah Kim, Oliver Kohlbacher, Dmitry Korkin, Kiryluk Krzysztof, Khalid Kunji, Zoltàn Kutalik, Kasper Lage, David Lamparter, Sean Lang-Brown, Thuc Duy Le, Jooyoung Lee, Sunwon Lee, Juyong Lee, Dong Li, Jiuyong Li, Junyuan Lin, Lin Liu, Antonis Loizou, Zhenhua Luo, Artem Lysenko, Tianle Ma, Raghvendra Mall, Daniel Marbach, Tomasoni Mattia, Mario Medvedovic, Jörg Menche, Johnathan Mercer, Elisa Micarelli, Alfonso Monaco, Felix Müller, Rajiv Narayan, Oleksandr Narykov, Ted Natoli, Thea Norman, Sungjoon Park, Livia Perfetto, Dimitri Perrin, Stefano Pirrò, Teresa M. Przytycka, Xiaoning Qian, Karthik Raman, Daniele Ramazzotti, Emilie Ramsahai, Balaraman Ravindran, Philip Rennert, Julio Saez-Rodriguez, Charlotta Schärfe, Roded Sharan, Ning Shi, Wonho Shin, Hai Shu, Himanshu Sinha, Donna K. Slonim, Lionel Spinelli, Suhas Srinivasan, Aravind Subramanian, Christine Suver, Damian Szklarczyk, Sabina Tangaro, Suresh Thiagarajan, Laurent Tichit, Thorsten Tiede, Beethika Tripathi, Aviad Tsherniak, Tatsuhiko Tsunoda, Dénes Türei, Ehsan Ullah, Golnaz Vahedi, Alberto Valdeolivas, Jayaswal Vivek, Christian von Mering, Andra Waagmeester, Bo Wang, Yijie Wang, Barbara A. Weir, Shana White, Sebastian Winkler, Ke Xu, Taosheng Xu, Chunhua Yan, Liuqing Yang, Kaixian Yu, Xiangtian Yu, Gaia Zaffaroni, Mikhail Zaslavskiy, Tao Zeng, Jitao D. Zhang, Lu Zhang, Weijia Zhang, Lixia Zhang, Xinyu Zhang, Junpeng Zhang, Xin Zhou, Jiarui Zhou, Hongtu Zhu, Junjie Zhu, Guido Zuccon, Aravind Subramanian, Jitao D. Zhang, Gustavo Stolovitzky, Zoltán Kutalik, Kasper Lage, Donna K. Slonim, Julio Saez-Rodriguez, Lenore J. Cowen, Sven Bergmann, Daniel Marbach

**Affiliations:** 1 Department of Computational Biology, University of Lausanne, Lausanne, Switzerland; 2 Swiss Institute of Bioinformatics, Lausanne, Switzerland; 3 Icahn Institute for Genomics and Multiscale Biology and Department of Genetics and Genomic Sciences, Icahn School of Medicine at Mount Sinai, New York, NY USA; 4 Department of Computer Science, Tufts University, Medford, MA USA; 5 Roche Pharma Research and Early Development, Pharmaceutical Sciences, Roche Innovation Center Basel, F. Hoffmann-La Roche Ltd, Basel, Switzerland; 6 Verge Genomics, San Francisco, CA USA; 7 Department of Mathematics, Tufts University, Medford, MA USA; 8 College of Computer and Information Science, Northeastern University, Boston, MA USA; 9 Department of Surgery, Massachusetts General Hospital, Harvard Medical School, Boston, MA USA; 10 Stanley Center at the Broad Institute of MIT and Harvard, Cambridge, MA USA; 11 Broad Institute of MIT and Harvard, Cambridge, MA USA; 13 IBM T.J. Watson Research Center, Yorktown Heights, NY USA; 14 University Institute of Primary Care and Public Health, University of Lausanne, Lausanne, Switzerland; 15 Institute for Biological Psychiatry, Mental Health Center Sct. Hans, University of Copenhagen, Roskilde, Denmark; 16 Department of Immunology, Tufts University School of Medicine, Boston, MA USA; 17 Institute for Computational Biomedicine, Faculty of Medicine, Heidelberg University, Bioquant, Heidelberg, Germany; 18 RWTH Aachen University, Faculty of Medicine, Joint Research Center for Computational Biomedicine, Aachen, Germany; 19 Department of Integrative Biomedical Sciences, University of Cape Town, Cape Town, South Africa; 22 Applied Bioinformatics, Center for Bioinformatics, University of Tübingen, Tübingen, Germany; 23 Department of Physics ‘Michelangelo Merlin’, University of Bari ‘Aldo Moro’, Bari, Italy; 24 INFN, Sezione di Bari, Bari, Italy; 25 Departament d’Enginyeria Informàtica i Matemàtiques, Universitat Rovira i Virgili, Tarragona, Spain; 26 Department of Biotechnology, Bhupat and Jyoti Mehta School of Biosciences, Indian Institute of Technology Madras, Chennai, India; 27 Initiative for Biological Systems Engineering, Indian Institute of Technology Madras, Chennai, India; 28 Robert Bosch Centre for Data Science and Artificial Intelligence, Indian Institute of Technology Madras, Chennai, India; 29 Department of Biostatistics and Medical Informatics, University of Wisconsin-Madison, Madison, WI USA; 30 Department of Computer Sciences, University of Wisconsin-Madison, Madison, WI USA; 31 Morgridge Institute for Research, Madison, WI USA; 32 Department of Plant Biology, Carnegie Institution for Science, Stanford, USA; 33 Department of Computer Science, Stanford University, Stanford, USA; 34 Aix Marseille University, CNRS, Centrale Marseille, I2M, Marseille, France; 35 Centro TIRES, Bari, Italy; 36 Department of Computational Biology, University of Lausanne, Lausanne, Switzerland; 37 Swiss Institute of Bioinformatics, Lausanne, Switzerland; 38 RIKEN Center for Integrative Medical Sciences, Yokohama, Japan; 39 Aix Marseille Univ, INSERM, TAGC, Marseille, France; 40 CNRS, Marseille, France; 41 Department of Genetics, Perelman School of Medicine at the University of Pennsylvania, Philadelphia, PA USA; 42 Institute for Immunology, Perelman School of Medicine at the University of Pennsylvania, Philadelphia, PA USA; 43 Epigenetics Institute, Perelman School of Medicine at the University of Pennsylvania, Philadelphia, PA USA; 44 CeMM Research Center for Molecular Medicine of the Austrian Academy of Sciences, Vienna, Austria; 45 Bioinformatics and Computational Biology Unit, Department of Biology, Tor Vergata University, Roma, Italy; 46 School of Computer Science, The University of Birmingham, Birmingham, UK; 47 Nestle Institute of Health Sciences, Lausanne, Switzerland; 48 Department of Computer Science, Tufts University, Medford, MA USA; 49 Department of Mathematics, Tufts University, Medford, MA USA; 50 Bioinformatics and Computational Biology Program, Worcester Polytechnic Institute, Worcester, MA USA; 51 National Center for Biotechnology Information, National Institute of Health (NCBI/NLM/NIH), Bethesda, MD USA; 52 Fondazione Bruno Kessler, Povo, Italy; 53 LCSB - Luxembourg Centre for Systems Biomedicine, University of Luxembourg, Esch-sur-Alzette, Luxembourg; 54 CIC bioGUNE, Bizkaia Technology Park, Derio, Spain; 55 IKERBASQUE, Basque Foundation for Science, Bilbao, Spain; 56 Roche Pharma Research and Early Development, Pharmaceutical Sciences, Roche Innovation Center Basel, F. Hoffmann-La Roche Ltd, Basel, Switzerland; 57 Department of Cancer Biology, University of Cincinnati, Cincinnati, OH USA; 58 Center for In Silico Protein Science, Korea Institute for Advanced Study, Seoul, Korea; 59 School of Computational Sciences, Korea Institute for Advanced Study, Seoul, Korea; 60 School of Informatics, Computing and Engineering, Indiana University, Bloomington, USA; 61 Algorithms in Bioinformatics, Center for Bioinformatics, University of Tübingen, Tübingen, Germany; 62 Department of Computational Medicine and Bioinformatics, University of Michigan, Ann Arbor, MI USA; 63 Micelio, Antwerp, Belgium; 64 College of Computer and Information Science, Northeastern University, Boston, MA USA; 65 National Cancer Institute, Center for Biomedical Informatics & Information Technology, Bethesda, MD USA; 66 Center for Bioinformatics and Computational Biology, University of Maryland, College Park, MD USA; 67 Department of Cell Biology and Molecular Genetics, University of Maryland, College Park, MD USA; 68 Department of Computer Science and Engineering, Korea University, Seoul, Korea; 69 Center for Advanced Computation, Korea Institute for Advanced Study, Seoul, Korea; 70 Interdisciplinary Graduate Program in Bioinformatics, Korea University, Seoul, Korea; 71 Community High School, Ann Arbor, MI USA; 72 Biomolecular Interactions, Max Planck Institute for Developmental Biology, Tübingen, Germany; 73 Quantitative Biology Center, University of Tübingen, Tübingen, Germany; 74 Institute for Translational Bioinformatics, University Hospital Tübingen, Tübingen, Germany; 75 Data Science Program, Worcester Polytechnic Institute, Worcester, MA USA; 76 Department of Computer Science, Worcester Polytechnic Institute, Worcester, MA USA; 77 Department of Medicine, College of Physicians & Surgeons, Columbia University, New York, NY USA; 78 Qatar Computing Research Institute, Hamad Bin Khalifa University, Doha, Qatar; 79 Institute of Social and Preventive Medicine (IUMSP), Lausanne University Hospital, Lausanne, Switzerland; 80 Department of Surgery, Massachusetts General Hospital, Harvard Medical School, Boston, Massachusetts USA; 81 Institute for Biological Psychiatry, Mental Health Center Sct. Hans, University of Copenhagen, Roskilde, Denmark; 82 Stanley Center at the Broad Institute of MIT and Harvard, Cambridge, Massachusetts USA; 83 Verge Genomics, San Francisco, CA USA; 84 Division of Geriatrics, Department of Medicine, University of California, San Francisco, USA; 85 Centre for Cancer Biology, University of South Australia, Adelaide, SA Australia; 86 School of Information Technology and Mathematical Sciences, University of South Australia, Adelaide, SA Australia; 87 Department of Chemistry, Kangwon National University, Chuncheon, Republic of Korea; 88 BlueSkyIt, Amsterdam, the Netherlands; 89 The Liver Care Center and Divisions of Gastroenterology, Hepatology and Nutrition, Cincinnati Children’s Hospital Medical Center, Cincinnati, OH USA; 90 Department of Computer Science and Engineering, University at Buffalo, Buffalo, NY USA; 91 Department of Environmental Health, Division of Biostatistics and Bioinformatics, University of Cincinnati, Cincinnati, OH USA; 92 Broad Institute of Harvard and MIT, Cambridge, MA USA; 93 Bill and Melinda Gates Foundation, Washington, USA; 94 School of Electrical Engineering and Computer Science, Queensland University of Technology, Brisbane, Australia; 95 Dept. of Electrical & Computer Engineering, Texas A&M University, College Station, USA; 96 Department of Mathematics and Statistics, The University of the West Indies, Saint Augustine, Trinidad and Tobago; 97 Department of Computer Science and Engineering, Indian Institute of Technology Madras, Chennai, India; 98 Rockville, MD USA; 99 Institute for Computational Biomedicine, Faculty of Medicine, Heidelberg University, Bioquant, Heidelberg, Germany; 100 RWTH Aachen University, Faculty of Medicine, Joint Research Centre for Computational Biomedicine, Aachen, Germany; 101 Blavatnik School of Computer Science, Tel Aviv University, Tel Aviv, Israel; 102 Department of Biostatistics, the University of Texas MD Anderson Cancer Center, Houston, TX USA; 103 Sage Bionetworks, Seattle, Washington USA; 104 Institute of Molecular Life Sciences and Swiss Institute of Bioinformatics, University of Zürich, Zürich, Switzerland; 105 Memphis, TN USA; 106 CREST, JST, Tokyo, Japan; 107 Department of Medical Science Mathematics, Medical Research Institute, Tokyo Medical and Dental University, Tokyo, Japan; 108 European Molecular Biology Laboratory, European Bioinformatics Institute, Wellcome Genome Campus, Cambridge, UK; 109 ProGeLife, Marseille, France; 110 Disease Science & Technology, Biocon Bristol-Myers Squibb Research Centre, Bangalore, India; 111 Broad Institute of MIT and Harvard, Cambridge, MA UK; 112 Janssen Research and Development, Cambridge, MA USA; 113 Department of Psychiatry, Yale School of Medicine, New Haven, CT USA; 114 Institute of Intelligent Machines, Hefei Institutes of Physical Science, Chinese Academy of Sciences, Hefei, Anhui China; 115 Department of Statistics and Operations Research, University of North Carolina at Chapel Hill, Chapel Hill, NC USA; 116 Key Laboratory of Systems Biology, Institute of Biochemistry and Cell Biology, Shanghai Institutes for Biological Sciences, Chinese Academy of Sciences, Shanghai, China; 117 Computational Biology Consulting, Paris, France; 118 School of Engineering, Dali University, Dali, Yunnan China; 119 Department of Electrical Engineering, Stanford University, Stanford, USA

**Keywords:** Cellular signalling networks, Functional clustering, Gene regulatory networks, Network topology, Population genetics

## Abstract

Many bioinformatics methods have been proposed for reducing the complexity of large gene or protein networks into relevant subnetworks or modules. Yet, how such methods compare to each other in terms of their ability to identify disease-relevant modules in different types of network remains poorly understood. We launched the ‘Disease Module Identification DREAM Challenge’, an open competition to comprehensively assess module identification methods across diverse protein–protein interaction, signaling, gene co-expression, homology and cancer-gene networks. Predicted network modules were tested for association with complex traits and diseases using a unique collection of 180 genome-wide association studies. Our robust assessment of 75 module identification methods reveals top-performing algorithms, which recover complementary trait-associated modules. We find that most of these modules correspond to core disease-relevant pathways, which often comprise therapeutic targets. This community challenge establishes biologically interpretable benchmarks, tools and guidelines for molecular network analysis to study human disease biology.

## Abstract (web summary)

In this DREAM challenge, 75 methods for the identification of disease-relevant modules from molecular networks are compared and validated with GWAS data. The authors provide practical guidelines for users and establish benchmarks for network analysis.

## Main

Complex diseases involve many genes and molecules that interact within cellular networks^1–3^. Advances in experimental and computational techniques enable both physical interaction networks (for example, protein–protein interaction, signaling and regulatory networks) and functional networks (for example, co-expression, genetic and single-cell interaction networks) to be mapped with increasing accuracy. A key problem in the analysis of these networks is the identification of functional units, called modules or pathways^4^. It is well-known that molecular networks have a high degree of modularity (that is, subsets of nodes are more densely connected than expected by chance), and that individual modules often comprise genes or proteins that are involved in the same biological functions^5^. Moreover, biological networks are typically too large to be examined as a whole. Consequently, module identification is often a crucial step to gain biological insights from network data^6–9^.

Module identification, also called community detection or graph clustering, is a classic problem in network science for which a wide range of methods have been proposed^10^. These methods are typically assessed on in silico generated benchmark graphs^11^. However, how well different approaches uncover biologically relevant modules in real molecular networks remains poorly understood. Crowdsourced data competitions (known as challenges) have proved to be an effective means to rigorously assess methods and foster collaborative communities. The Dialogue on Reverse Engineering and Assessment (DREAM) is a community-driven initiative promoting data challenges in biomedicine (http://dreamchallenges.org). DREAM challenges have established robust methodologies for diverse problems, including the inference of molecular networks^12,13^. But, so far there has been no community effort addressing the downstream analysis of reconstructed networks.

**Fig. 1:**
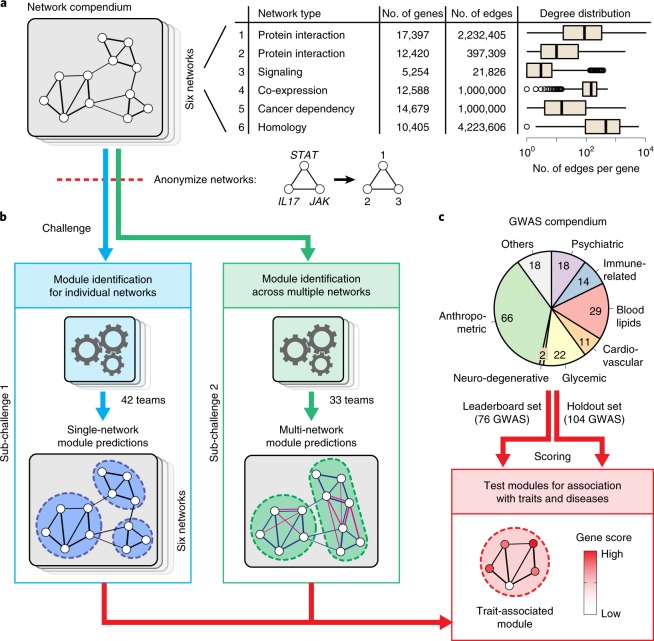
The Disease Module Identification DREAM Challenge. **a**, Network types included in the challenge. Throughout the paper, boxplot center lines show the median, box limits show upper and lower quartiles, whiskers show 1.5× interquartile range and points show outliers. **b**, Outline of the challenge. **c**, Outline of the scoring.

Here we present the Disease Module Identification DREAM Challenge, where over 400 participants from all over the world predicted disease-relevant modules in diverse gene and protein networks (Fig. 1; https://synapse.org/modulechallenge). We introduce community-driven benchmarks, dissect top-performing approaches and explore the biology of discovered network modules.

## Results

### A community challenge to assess network module identification methods

We developed a panel of diverse, human molecular networks for the challenge, including custom versions of two protein–protein interaction and a signaling network extracted from the STRING^14^, InWeb^15^ and OmniPath^16^ databases, a co-expression network inferred from 19,019 tissue samples from the Gene Expression Omnibus (GEO) repository^17^, a network of genetic dependencies derived from loss-of-function screens in 216 cancer cell lines^18,19^ and a homology-based network built from phylogenetic patterns across 138 eukaryotic species^20,21^ (Methods). We included different types of network, which also vary in their size and structural properties, to provide a heterogeneous benchmark resource (Fig. 1a).

Each network was generated specifically for the challenge and released in anonymized form (that is, we did not disclose the gene names and the identity of the networks), thus enabling rigorous ‘blinded’ assessment. That is, participants could only use unsupervised clustering algorithms, which rely exclusively on the network structure and do not depend on additional biological information such as known disease genes.

We solicited participation in two types of module identification challenge (Fig. 1b). In Sub-challenge 1, solvers were asked to run module identification on each of the provided networks individually (single-network module identification). In Sub-challenge 2, the networks were reanonymized in a way that the same gene identifier represented the same gene across all six networks. Solvers were then asked to identify a single set of non-overlapping modules by sharing information across the six networks (multi-network module identification), which allowed us to assess the potential improvement in performance offered by emerging multi-network methods compared to single-network methods. In both sub-challenges, predicted modules had to be non-overlapping and comprise between 3 and 100 genes.

The challenge was run using the open-science Synapse platform^22^. Over a 2-month period, participants could make a limited number of submissions and see the performance of all teams on a real-time leaderboard. In the final round, teams could make a single submission for each sub-challenge, which had to include method descriptions and code for reproducibility.

### Biologically interpretable scoring of modules based on trait associations

Evaluation of predicted modules is challenging because there is no ground truth of ‘correct’ modules in molecular networks. Here, we introduce a framework to empirically assess modules based on their association with complex traits and diseases using genome-wide association studies (GWAS) data (Fig. 1c). Since GWAS are based on data completely different from those used to construct the networks, they can provide independent support for biologically relevant modules. To cover diverse molecular processes, we have compiled a large collection of 180 GWAS datasets (Supplementary Table 1). Predicted modules were scored on each GWAS using the Pascal tool^23^, which aggregates trait-association *P* values of single nucleotide polymorphisms (SNPs) at the level of genes and modules. Modules that scored significantly for at least one GWAS trait were called trait-associated. Finally, the score of each challenge submission was defined as the total number of its trait-associated modules (at 5% false discovery rate (FDR), see Methods).

To detect potential overfitting, the collection of 180 GWASs was split into a leaderboard set for scoring the leaderboard submissions and a separate holdout set for scoring the single, final submission of each team. Results reported below are from the final evaluation on the holdout set.

### Top methods from different categories achieve comparable performance

**Fig. 2:**
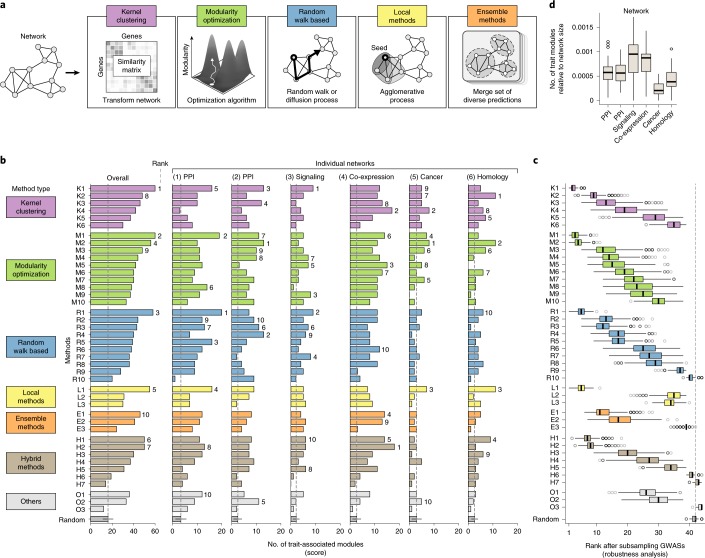
Assessment of module identification methods. **a**, Main types of module identification approach used in the challenge. **b**, Final scores of the 42 module identification methods applied in Sub-challenge 1 for each of the six networks, as well as the overall score summarizing performance across networks (evaluated using the holdout GWAS set at 5% FDR; method IDs are defined in Supplementary Table 2). Ranks are indicated for the top ten methods. The last row shows the mean performance of 17 random modularizations of the networks (error bars show the standard deviation). **c**, Robustness of the overall ranking was evaluated by subsampling the GWAS set used for evaluation 1,000 times. For each method, the resulting distribution of ranks is shown as a boxplot. **d**, Number of trait-associated modules per network. Boxplots show the number of trait-associated modules across the 42 methods, normalized by the size of the respective network.

The community contributed 42 single-network and 33 multi-network module identification methods in the final round of the two sub-challenges. We first discuss the single-network methods (Sub-challenge 1), which we grouped into seven broad categories: (1) kernel clustering, (2) modularity optimization, (3) random-walk-based, (4) local methods, (5) ensemble methods, (6) hybrid methods and (7) other methods (Fig. 2a and Supplementary Table 2). While many teams adapted existing algorithms for community detection, other teams—including the best performers—developed novel approaches. The top five methods achieved comparable performance with scores between 55 and 60, while the remaining methods did not get to scores above 50 (Fig. 2b). Although the scores were close, the top-scoring method K1 (method IDs are defined in Supplementary Table 2) performed more robustly than runner-up methods, achieving the best score: (1) in the leaderboard and final rounds (Supplementary Table 3); (2) at varying FDR cutoffs (Supplementary Fig. 1) and (3) on subsamples of the GWAS holdout set (Fig. 2c).

The top teams used different approaches: the best performers (K1) developed a novel kernel approach leveraging a diffusion-based distance metric^24,25^ and spectral clustering^26^; the runner-up team (M1) extended different modularity optimization methods with a resistance parameter that controls the granularity of modules^27^ and the third-ranking team (R1) used a random-walk method based on Markov clustering with locally adaptive granularity to balance module sizes^28^ (Methods). These teams further collaborated after the challenge to bundle their methods in a user-friendly tool^29^.

Four different method categories are represented among the top five performers, suggesting that no single approach is inherently superior for module identification. Rather, performance depends on the specifics of each individual method, including the strategy used to define the resolution (the number and size of modules). Preprocessing steps also affected performance: many of the top teams first sparsified the networks by discarding weak edges. A notable exception is the top method (K1), which performed robustly without any preprocessing of the networks.

The challenge also allows us to explore how informative different types of molecular network are for finding modules underlying complex traits. In absolute numbers, methods recovered the most trait-associated modules in the co-expression and protein–protein interaction networks (Supplementary Fig. 1). However, relative to the network size, the signaling network contained the most trait modules (Fig. 2d). These results are consistent with the importance of signaling pathways for many of the considered traits and diseases. The cancer cell line and homology-based networks, on the other hand, were less relevant for the traits in our GWAS compendium and thus comprised only a few trait modules.

### Complementarity of different module identification approaches

**Fig. 3:**
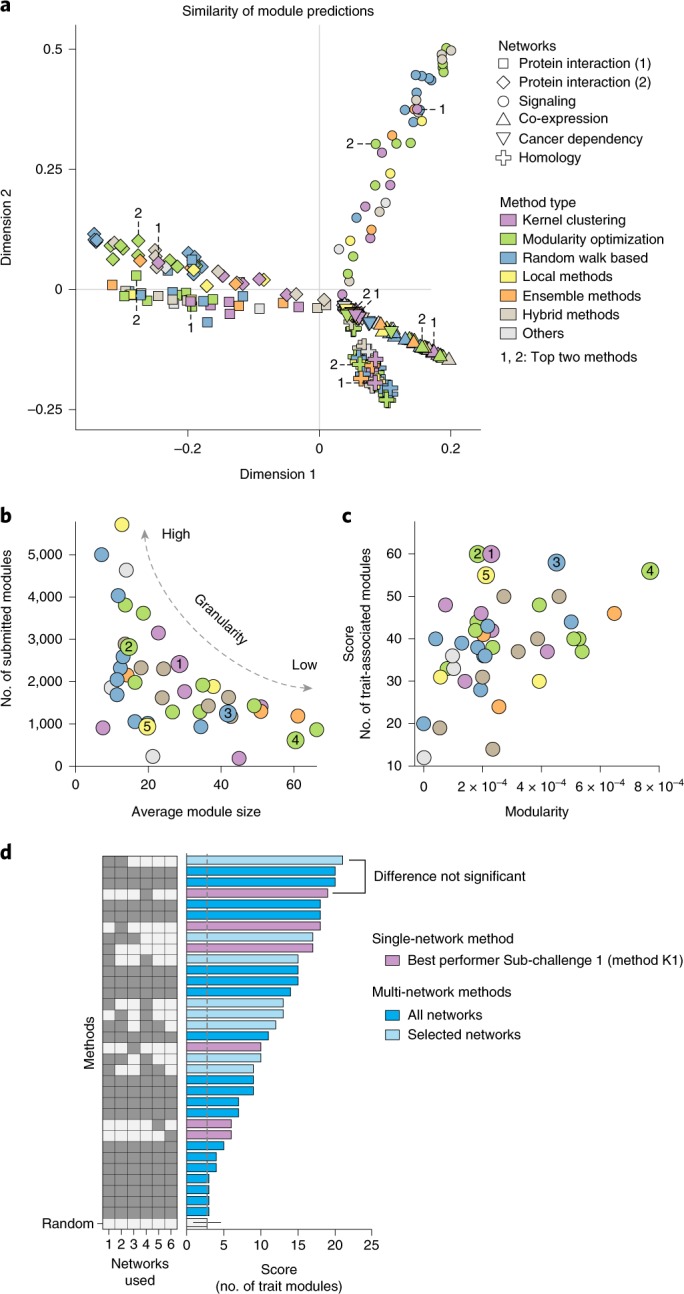
Complementarity of module predictions from different methods and networks. **a**, Similarity of module predictions from different methods (color) and networks (shape). The closer two points are in the plot, the more similar the corresponding module predictions (multidimensional scaling, see Methods). The top two methods are highlighted for each network. **b**, Total number of predicted modules versus average module size for each method (same color scheme as in **a**). The top five methods (numbered) produced modular decompositions of varying granularity. **c**, Challenge score (number of trait-associated modules) versus modularity is shown for each of the 42 methods (same color scheme as in **a**). Modularity is a topological quality metric for modules based on the fraction of within-module edges^43^. **d**, Final scores of multi-network module identification methods in Sub-challenge 2 (evaluated using the holdout GWAS set at 5% FDR). For comparison, the overall best-performing method from Sub-challenge 1 is also shown (method K1, purple). Teams used different combinations of the six challenge networks for their multi-network predictions (shown on the left). The difference between the top single-network module predictions and the top multi-network module predictions is not significant when subsampling the GWASs (Bayes factor < 3, Supplementary Fig. 5). The last row shows the mean performance of 17 random modularizations of the networks (error bars show standard deviation).

To test whether predictions from different methods and networks tend to capture the same or complementary modules, we applied a pairwise similarity metric to all 252 module predictions from Sub-challenge 1 (42 methods × 6 networks, see Methods). We find that similarity of module predictions is primarily driven by the underlying network and top-performing methods do not converge to similar module predictions (Fig. 3a and Supplementary Fig. 2). Indeed, only 46% of trait modules are recovered by multiple methods with good agreement in a given network (high overlap or submodules, Supplementary Fig. 2). Across different networks, the number of recovered modules with substantial overlap is even lower (17%). Thus, the majority of trait modules are method- and network-specific.

The modules produced by different methods also vary in terms of their structural properties. For example, submissions included between 16 and 1,552 modules per network, with an average module size ranging from 7 to 66 genes. Neither the number nor the size of submitted modules correlates with performance (Fig. 3b and Supplementary Figs. 3 and 4). Thus, there is no single optimal granularity for a given network; rather, different methods captured trait-relevant modules at varying levels of granularity. Topological quality metrics of modules such as modularity showed only modest correlation with the challenge score (Pearson’s *r* = 0.45, Fig. 3c), highlighting the need for biologically interpretable assessment of module identification methods.

### Multi-network module identification methods did not provide added power

In Sub-challenge 2, teams submitted a single modularization of the genes, for which they could leverage information from all six networks together. While some teams developed dedicated multi-network (multi-layer) community detection methods^30,31^, the majority of teams first merged the networks and then applied single-network methods (Methods).

It turned out to be very difficult to effectively leverage complementary networks for module identification. While three teams achieved marginally higher scores than single-network module predictions (Fig. 3d), the difference is not significant when subsampling the GWASs (Bayes factor < 3, Supplementary Fig. 5). Moreover, the best-scoring team simply merged the two protein–protein interaction networks (the two most similar networks, Supplementary Fig. 6), discarding the other types of network. Since no significant improvement over single-network methods was achieved, the winning position of Sub-challenge 2 was declared vacant.

### Integration of challenge submissions leads to robust consensus modules

To derive consensus modules from team submissions, we integrated module predictions from different methods in a consensus matrix *C*, where each element *c*_*ij*_ is proportional to the number of methods that put gene *i* and *j* together in the same module. The consensus matrix was then clustered using the top-performing module identification method from the challenge (Methods).

We generated consensus modules for each challenge network by applying this approach to the top 21 (50%) of methods from the leaderboard round. The score of the consensus modules outperforms the top individual method predictions in both sub-challenges (Supplementary Figs. 1 and 5). However, when applied to fewer methods, the performance of the consensus drops (Supplementary Fig. 7). We conclude that the consensus approach is only suitable in a challenge context, since applying such a large number of methods is not practical for users. Indeed, we found that the total number of trait-associated modules when considering the modules from top-performing methods individually is higher than the number of modules resulting from their consensus (Supplementary Fig. 8).

### Network modules reveal trait-specific and shared pathways

**Fig. 4:**
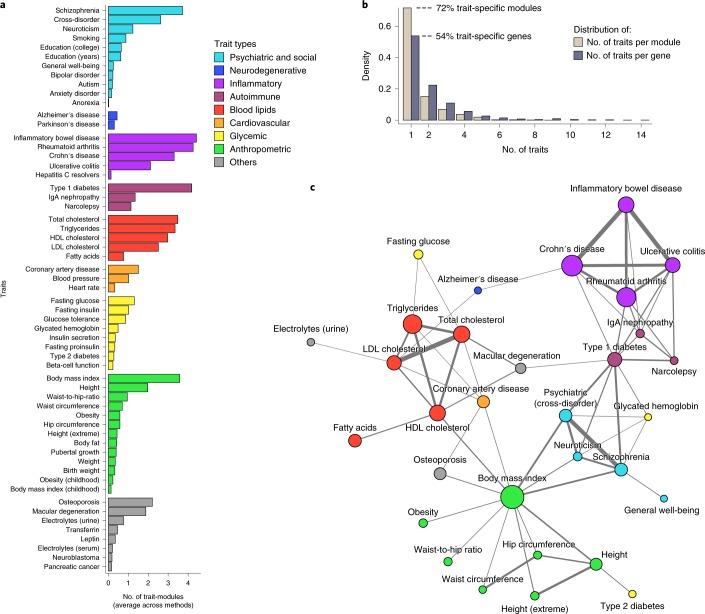
Overlap between modules associated with different traits and diseases. **a**, Average number of trait-associated modules identified by challenge methods for each trait in Sub-challenge 1. For traits where multiple GWASs were available, results for the best-powered study are shown. HDL, high-density lipoprotein; LDL, low-density lipoprotein. **b**, Histograms showing the number of distinct traits per trait-associated module (brown) and gene (gray). **c**, Trait network showing similarity between GWAS traits based on overlap of associated modules (force-directed graph layout). Node size corresponds to the number of genes in trait-associated modules and edge width corresponds to the degree of overlap (Jaccard index, only edges for which the overlap is significant are shown (Bonferroni-corrected hypergeometric *P* < 0.05, see Methods)). Traits without any edges are not shown.

We next sought to explore biological properties of predicted modules. The most trait-associated modules were found for immune-related, psychiatric, blood cholesterol and anthropometric traits, for which high-powered GWAS are available that are known to show strong pathway enrichment (Fig. 4a). Significant GWAS loci often show association to multiple traits. Across our GWAS compendium, we found that 46% of trait-associated genes but only 28% of trait-associated modules are associated with multiple traits (Fig. 4b). Thus, mapping genes onto network modules may help in disentangling trait-specific pathways at shared loci.

We next asked which traits are similar in terms of the implicated network components. To this end, we considered the union of all genes within network modules associated with a given trait (trait-module genes). We then evaluated the pairwise similarity of traits based on the significance of the overlap between the respective trait-module genes (Methods). Trait relationships thus inferred are consistent with known biology and comorbidities between the considered traits and diseases (Fig. 4c).

### Trait-associated modules implicate core disease genes and pathways

Due to linkage disequilibrium (LD), many genes that show association to a trait may not causally influence it. A key question is whether our trait modules, and the corresponding genes, are correctly predicted as being biologically or therapeutically relevant for that trait or disease. We thus sought to evaluate trait modules using additional independent datasets, including ExomeChip data, monogenic disease genes, functional annotations and known therapeutic targets.

**Fig. 5:**
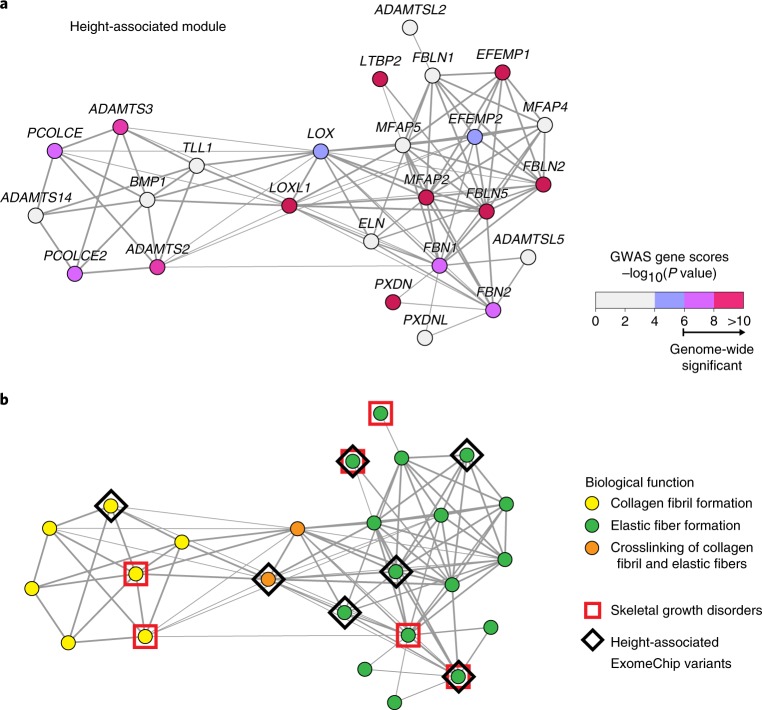
Support for trait-module genes in diverse datasets. **a**, Example module from the consensus analysis in the STRING protein–protein interaction network (force-directed graph layout). The module is associated to height (*n* = 25 genes, FDR-corrected Pascal *P* = 0.005, see Methods). Color indicates Pascal GWAS gene scores (Methods). The module includes genes that are genome-wide significant (magenta and pink) as well as genes that do not reach the genome-wide significance threshold, but are predicted to be involved in height due to their module membership (blue and gray). **b**, Member genes of the height-associated module are supported by independent datasets: 24% of module genes are implicated in monogenic skeletal growth disorders (red squares, enrichment *P* = 7.5 × 10^−4^ (one-sided Fisher’s exact test)) and 28% of module genes have coding variants associated to height in an ExomeChip study published after the challenge^32^ (black diamonds, enrichment *P* = 1.9 × 10^−6^). The form of this module follows its function: two submodules comprise proteins involved in collagen fibril (yellow) and elastic fiber formation (green), while the proteins that link these submodules (orange) indeed have the biological function of crosslinking collagen fibril and elastic fibers.

We first consider a module from the consensus analysis that shows association to height—a classic polygenic trait—as an example (Fig. 5a). Forty percent of genes in this module either comprise coding variants associated to height in an independent ExomeChip study^32^ or are known to be implicated in monogenic skeletal growth disorders, supporting their causal role in the phenotype (Fig. 5b). Gene Ontology (GO) annotations further show that this module consists of two submodules comprising extracellular matrix proteins responsible for, respectively, collagen fibril and elastic fiber formation—pathways that are essential for growth (Fig. 5b). Indeed, mutations of homologous genes in mouse lead to abnormal elastic fiber morphology (Supplementary Fig. 9). Some of the genes supported by these additional datasets did not show signal in the GWAS used to discover the module. For example, the module gene *BMP1* (*Bone Morphogenic Protein 1*) causes osteogenesis imperfecta, which is associated with short stature. Yet, *BMP1* does not show association to height in current GWAS and ExomeChip studies, demonstrating how network modules can implicate additional disease-relevant pathway genes (see Supplementary Figs. 10 and 11 for a comprehensive evaluation of prioritized trait-module genes).

**Fig. 6:**
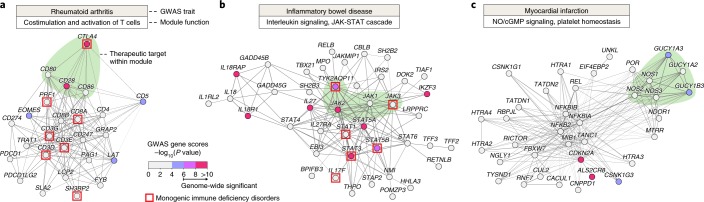
Example trait modules comprising therapeutically relevant pathways. **a**–**c**, The modules are from the STRING protein–protein interaction networks and were generated using the consensus method. Node colors correspond to Pascal gene scores in the respective GWAS (Methods). For the two inflammatory disorders (**a**,**b**), red squares indicate genes causing monogenic immunodeficiency disorders (enrichment *P* values of 4.1 × 10^−^^8^ and 1.2 × 10^−6^, respectively (one-sided Fisher’s exact test)). **a**, Module associated with rheumatoid arthritis (*n* = 25 genes, FDR-corrected Pascal *P* = 0.04) that is involved in T cell activation. A costimulatory pathway is highlighted green, T cell response is regulated by activating (*CD28*) and inhibitory (*CTLA4*) surface receptors, which bind B7 family ligands (*CD80* and *CD86*) expressed on the surface of activated antigen-presenting cells. The therapeutic agent CTLA4-Ig binds and blocks B7 ligands, thus inhibiting T cell response. **b**, Cytokine signaling module associated with inflammatory bowel disease (*n* = 42 genes, FDR-corrected Pascal *P* = 0.0006). The module includes the four known Janus kinases (*JAK1-3* and *TYK2*, highlighted green), which are engaged by cytokine receptors to mediate activation of specific transcription factors (*STATs*). Inhibitors of JAK–STAT signaling are being tested in clinical trials for both ulcerative colitis and Crohn’s disease^44^. **c**, Module associated with myocardial infarction (*n* = 36 genes, FDR-corrected Pascal *P* = 0.0001) comprising two main components of the NO/cGMP signaling pathway (endothelial nitric oxide synthases (*NOS1-3*) and soluble guanylate cyclases (*GUCY1A2*, *GUCY1A3* and *GUCY1B3*), highlighted green), a key therapeutic target for cardiovascular disease^45^.

To evaluate more generally whether trait-associated modules correspond to generic or disease-specific pathways, we systematically tested modules for functional enrichment of GO annotations, mouse mutant phenotypes and pathway databases. We further selected the most representative annotations for each module using a regression framework^33^ (Methods). We find that the majority of trait modules reflect core disease-specific pathways. For example, in the STRING protein–protein interaction network only 33% of trait modules from the consensus analysis have generic functions; the remaining 66% of trait modules correspond to core disease-specific pathways, some of which are therapeutic targets (Supplementary Fig. 12 and Supplementary Table 4). Examples include a module associated with rheumatoid arthritis that comprises the B7:CD28 costimulatory pathway required for T cell activation, which is blocked by an approved drug (Fig. 6a); a module associated with inflammatory bowel disease corresponding to cytokine signaling pathways mediated by Janus kinases (JAKs), which are therapeutic targets (Fig. 6b) and a module associated with myocardial infarction that includes the NO/cGMP signaling cascade, which plays a key role in cardiovascular pathophysiology and therapeutics (Fig. 6c). We further applied our pipeline to a GWAS on IgA nephropathy (IgAN) obtained after the challenge, an autoimmune disorder with poorly understood etiology^34^. We find two IgAN-associated modules, which prioritize novel candidate genes involved in NF-kB signaling, complement and coagulation cascades, demonstrating how our challenge resources can be used for network-based analysis of new GWAS datasets (Supplementary Fig. 13).

## Discussion

As large-scale network data become pervasive in many fields, robust tools for detection of network communities are of critical importance. With this challenge we have conducted an impartial and interpretable assessment of module identification methods on biological networks, providing much-needed guidance for users. While it is important to keep in mind that the exact ranking of methods is specific to the task and datasets considered, the resulting collection of top-performing module identification tools and methodological insights will be broadly useful for modular analysis of complex networks in biology and other domains.

Kernel clustering, modularity optimization, random-walk-based and local methods were all represented among the top performers, suggesting that no single type of approach is inherently superior. In contrast, the popular weighted gene co-expression network analysis (WGCNA) method^7^ did not perform competitively, likely because it relies on hierarchical clustering, which—unlike the top-performing approaches—was not specifically designed for network clustering. Moreover, while most published studies in network biology rely on a single clustering method, the results of this challenge demonstrate the value of applying multiple methods from different categories to detect complementary types of module.

The challenge further emphasized the importance of the resolution (size and number of modules). Biological networks typically have a hierarchical modular structure, which implies that disease-relevant pathways can be captured at different levels of granularity^35^. Indeed, we found that there is no intrinsic optimal resolution for a given network; rather, it depends on the type of method used (Supplementary Fig. 3). Top-performing challenge methods allowed the resolution to be tuned, enabling users to explore different module granularities.

Our analysis showed that signaling, protein–protein interaction and co-expression networks comprise complementary trait-relevant modules. Considering different types of network is thus clearly advantageous. However, multi-network module identification methods that attempted to reveal integrated modules across these networks failed to significantly improve predictions compared to methods that considered each network individually. These results are contrary to the common assumption that multi-network integration improves module predictions. However, this finding remains specific to the challenge networks, which may not have been sufficiently related. Indeed, the best result was obtained by the team merging only the two most related networks (the two protein–protein interaction networks), and the runner-up team confirmed in a post hoc analysis that focusing on networks with similar modular structures improved their results^36^. Multi-network methods may thus be better suited to networks that are more closely related, possibly from the same tissue- and disease-context^37^.

On the basis of these challenge findings, we make the following practical recommendations for module identification: (1) methods from diverse categories should be applied to identify complementary modules (for example, the top three challenge methods, which are available in a user-friendly tool^29^); (2) the resulting modules from different methods should be used as is, without forming a consensus (consensus modules were only competitive when integrating over 20 methods); (3) whenever possible, diverse networks should be leveraged (for example, co-expression, protein–protein interaction and signaling), as they comprise complementary types of module; (4) module identification methods should first be applied to each network individually, without merging the networks and (5) multi-network methods may be used to reveal modules in layered networks, but performance depends heavily on whether networks are sufficiently related.

There is continuing debate over the value of GWASs for revealing disease mechanisms and therapeutic targets. Indeed, the number of GWAS hits continues to grow as sample sizes increase, but the bulk of these hits does not correspond to core genes with specific roles in disease etiology^38^. While thousands of genes may show association to a given disease, we have demonstrated that much more specific disease modules comprising only dozens of genes can be identified within networks. These modules prioritize novel candidate genes, reveal pathway-level similarity between diseases and correspond to core disease pathways in the majority of cases. This is consistent with the robustness of biological networks: presumably, the many genes that influence disease indirectly are broadly distributed across network modules, while core disease genes cluster in specific pathways underlying pathophysiological processes^39,40^.

In this study we used generic networks, not context-specific networks, because the focus was on method assessment across diverse disorders. In the near future, we expect much more detailed maps of tissue- and disease-specific networks, along with diverse high-powered genetic datasets, to become available^2,41,42^. We hope that our challenge resources will provide a foundation to dissect these networks and reveal pathways implicated in human disease.

## Methods

### Network compendium

A collection of six gene and protein networks for human were provided by different groups for this challenge. The two protein–protein interaction and signaling networks are custom or new versions of existing interaction databases that were not publicly available at the time of the challenge. The remaining networks were yet unpublished at the time of the challenge. This was important to prevent participants from deanonymizing challenge networks by aligning them to the original networks.

Networks were released for the challenge in anonymized form. Anonymization consisted in replacing the gene symbols with randomly assigned ID numbers. In Sub-challenge 1, each network was anonymized individually; that is, node *k* of network A and node *k* of network B are generally not the same genes. In Sub-challenge 2, all networks were anonymized using the same mapping; that is, node *k* of network A and node *k* of network B are the same gene.

All networks are undirected and weighted, except for the signaling network, which is directed and weighted. Below we briefly summarize each of the six networks. Detailed descriptions of networks 4, 5 and 6 are available on GeNets^20^, a web platform for network-based analysis of genetic data (http://apps.broadinstitute.org/genets).

The first network was obtained from STRING, a database of known and predicted protein–protein interactions^14^. STRING includes aggregated interactions from primary databases as well as computationally predicted associations. Both physical protein interactions (direct) and functional associations (indirect) are included. The challenge network corresponds to the human protein–protein interactions of STRING v.10.0, where interactions derived from text mining were removed. Edge weights correspond to the STRING association score after removing evidence from text mining.

The second network is the InWeb protein–protein interaction network^15^. InWeb aggregates physical protein–protein interactions from primary databases and the literature. The challenge network corresponds to InWeb v.3. Edge weights correspond to a confidence score that integrates the evidence of the interaction from different sources.

The third network is the OmniPath signaling network^16^. OmniPath integrates literature-curated human signaling pathways from 27 different sources, of which 20 provide causal interaction and seven deliver undirected interactions. These data were integrated to form a directed weighted network. The edge weights correspond to a confidence score that summarizes the strength of evidence from the different sources.

The fourth network is a co-expression network based on Affymetrix HG-U133 Plus 2 arrays extracted from the GEO^46^. To adjust for non-biological variation, data were rescaled by fitting a loess-smoothed power law curve to a collection of 80 reference genes (ten sets of roughly eight genes each, representing different strata of expression) using non-linear least squares regression within each sample. All samples were then quantile normalized together as a cohort^17^. After filtering out samples that did not pass quality control, a gene expression matrix of 22,268 probesets by 19,019 samples was obtained. Probes were mapped to genes by averaging and the pairwise Spearman correlation of genes across samples was computed. The matrix was thresholded to include the top 1 million strongest positive correlations resulting in an undirected, weighted network. The edge weights correspond to the correlation coefficients.

The fifth network is a functional gene network derived from the Project Achilles dataset v.2.4.3 (ref. ^18^). Project Achilles performed genome-scale loss-of-function screens in 216 cancer cell lines using massively parallel pooled short-hairpin RNA screens. Cell lines were transduced with a library of 54,000 shRNAs, each targeting one of 11,000 genes for RNA interference knockdown (~5 shRNAs per gene). The proliferation effect of each shRNA in a given cell line could be assessed using Next Generation Sequencing. From these data, the dependency of a cell line on each gene (the gene essentiality) was estimated using the ATARiS method. This led to a gene essentiality matrix of 11,000 genes by 216 cell lines. Pairwise correlations between genes were computed and the resulting codependency network was thresholded to the top 1M strongest positive correlations, analogous to how the co-expression network was constructed.

The sixth network is a functional gene network based on phylogenetic relationships identified using the CLIME (clustering by inferred models of evolution) algorithm^21^. CLIME can be used to expand pathways (gene sets) with additional genes using an evolutionary model. Briefly, given a eukaryotic species tree and homology matrix, the input gene set is partitioned into evolutionarily conserved modules (ECMs), which are then expanded with new genes sharing the same evolutionary history. To this end, each gene is assigned a log-likelihood ratio (LLR) score based on the ECMs inferred model of evolution. CLIME was applied to 1,025 curated human gene sets from GO and the Kyoto Encyclopedia of Genes and Genomes using a 138 eukaryotic species tree, which resulted in 13,307 expanded ECMs. The network was constructed by adding an edge between every pair of genes that co-occurred in at least one ECM. Edge weights correspond to the mean LLR scores of the two genes.

### Challenge structure

Participants were challenged to apply network module identification methods to predict functional modules (gene sets) based on network topology. Valid modules had to be non-overlapping (a given gene could be part of either zero or one module, but not multiple modules) and comprise between 3 and 100 genes (modules with over 100 genes are typically less useful to gain specific biological insights). Modules did not have to cover all genes in a network. The number of modules per network was not fixed: teams could submit any number of modules for a given network (the maximum number was limited due to the fact that modules had to be non-overlapping). In Sub-challenge 1, teams were required to submit a separate set of modules for each of the six networks. In Sub-challenge 2, teams were required to submit a single set of modules by integrating information across multiple networks (it was permitted to use only a subset of the six networks).

The challenge consisted of a leaderboard phase and the final evaluation. The leaderboard phase was organized in four rounds, where participants could make repeated submissions and see their score for each network, along with the scores of other teams, on a real-time leaderboard. Due to the high computational cost of scoring the module predictions on a large number of GWAS datasets, a limit for the number of submissions per team was set in each round. The total number of submissions that any given team could make over the four leaderboard rounds was thus limited to only 25 and 41 for the two sub-challenges, respectively. For the final evaluation, a single submission including method descriptions and code was required per team, which was scored on a separate holdout set of GWASs after the challenge closed to determine the top performers.

With this challenge we assessed unsupervised clustering methods that define modules based solely on the topological structure of networks, unbiased by existing biological knowledge. Additional biological information, such as GWAS data and functional annotations, was integrated afterwards to assess and characterize the predicted modules. It is important to benchmark unsupervised methods in this blinded setting, because they are often relied on in regions of the network for which a paucity of biological information is currently available.

The submission format and rules are described in detail on the challenge website (https://www.synapse.org/modulechallenge).

### Gene and module scoring using Pascal

We have developed a framework to empirically assess module identification methods on molecular networks using GWAS data. Since we are employing a large collection of 180 GWAS datasets ranging over diverse disease-related human phenotypes, this approach covers a broad spectrum of molecular processes. In contrast to evaluation of module enrichment using existing gene and pathway annotations, where it is sometimes difficult to ascertain that annotations were not derived from similar data types as the networks (for example, gene expression, protein–protein interactions or homology), the GWAS-based approach provides an orthogonal means to assess disease-relevant modules.

SNP trait-association *P* values from a given GWAS were integrated across genes and modules using the Pascal (pathway scoring algorithm) tool^23^. Briefly, Pascal combines analytical and numerical solutions to efficiently compute gene and module scores from SNP *P* values, while properly correcting for LD correlation structure prevalent in GWAS data. To this end, LD information from a reference population is used (here, the European population of the 1,000 Genomes Project was employed as we only included GWASs with predominantly European cohorts). For gene scores we used the sum of chi-squared statistics of all SNPs within a window extending 50 kb up and downstream from the gene of interest. Since proximal SNPs are often in LD, under the null hypothesis this sum is not distributed like a sum of chi-squares of independent random variables. Yet, a change of basis to orthogonal ‘eigen-SNPs’, which diagonalize the genotypic correlation (LD) matrix, recovers independence with the effect that the sum of independent chi-squares is weighted (with the eigenvalues as weights)^23^.

The fast gene scoring is critical as it allows module genes that are in LD, and can thus not be treated independently, to be dynamically rescored. This amounts to fusing the genes of a given module that are in LD and computing a new score that takes the full LD structure of the corresponding locus into account. Pascal tests modules for enrichment in high-scoring (potentially fused) genes using a modified Fisher method, which avoids any *P* value cutoffs inherent to standard binary enrichment tests. The general approach can be summarized in three steps: (1) gene score *P* values of all genes in the background set (here, all genes in a given network) are transformed so that they follow a target distribution respecting their ranking; (2) a test statistic for a given module is computed by summing the transformed scores of module genes (and fusion-genes) and (3) it is evaluated whether the observed test statistic is higher than expected, that is, the module is enriched for trait-associated genes. Specifically, here we employed the ‘chi-squared method’ implemented in Pascal^23^, which transforms gene scores such that they follow a $\chi _1^2$-distribution (gene scores are first rank-transformed to obtain a uniform distribution and then transformed by the $\chi _1^2$-quantile function). $\chi _1^2$-gene scores of a given module of size *m* are then summed and tested against a $\chi _m^2$-distribution. Since gene scores are first rank-transformed, this is a ‘competitive’ enrichment test, which evaluates whether the module genes tend to have lower trait-association *P* values than the other genes that are part of the given network. Note that specifying the correct background set is critical for competitive enrichment tests, which here amounts to all genes that are part of the given network (that is, not all genes in the genome), as shown in Supplementary Fig. 4. Last, the resulting nominal module *P* values were adjusted to control the FDR via the Benjamini–Hochberg procedure.

### Scoring metric

In Sub-challenge 1, the score for a given network was defined as the number of modules with significant Pascal *P* values at a given FDR cutoff in at least one GWAS (called trait-associated modules, see previous section). Thus, modules that were hits for multiple GWAS traits were only counted once. The reason for this choice is that we do not want to ‘overcount’ modules that are hits for multiple related GWAS traits compared to modules that are hits for GWASs where few related traits are available (see Fig. 4c). The overall score was defined as the sum of the scores obtained on the six networks (that is, the total number of trait-associated modules across all networks). For the official challenge ranking a 5% FDR cutoff was defined, but performance was further reported at 10, 2.5 and 1% FDR.

Before the challenge, we performed an analysis to explore whether this scoring metric would favor a particular resolution for modules and thus bias results; for example, toward decomposing modules into a larger number of small submodules. To this end, we generated random modules of varying size. We found no systematic bias in the scores for a specific module granularity and this result was confirmed in the challenge (Supplementary Figs. 3 and 4). The key element of the scoring function that was designed to fairly assess module collections with different average module sizes was the higher multiple testing burden applied when a larger number of smaller modules was submitted.

Module predictions in Sub-challenge 2 were scored using the exact same methodology and FDR cutoffs. The only difference to Sub-challenge 1 was that submissions consisted of a single set of modules (instead of one for each network) and there was thus no need to define an overall score. As background gene set, the union of all genes across the six networks was used.

### Leaderboard and holdout GWAS datasets

We compiled a collection of 180 GWAS datasets (Supplementary Table 1), including all GWASs for which we could access genome-wide summary statistics (SNP *P* values). We deliberately included both high- and low-powered studies to evaluate whether disease-associated modules could be detected in datasets of varying signal strength. We manually assigned each GWAS dataset to either the leaderboard set used in the leaderboard round or the holdout set used for the final scoring. The assignment was made such that GWASs of closely related traits (for example, height, male height and female height) were either all in the leaderboard set or all in the holdout set, thus avoiding a situation where two very similar GWASs would be found both in the leaderboard and holdout set. This resulted in a leaderboard set of 76 GWASs and a holdout set of 104 GWASs (Supplementary Table 1). Compared to random assignment of GWASs to the leaderboard and holdout set, this setup better tests the robustness of parameters tuned by participants during the leaderboard round.

### Robustness analysis of challenge ranking

To gain a sense of the robustness of the ranking with respect to the GWAS data, we subsampled the set of 104 GWASs used for the final evaluation (the holdout set) by drawing *N* < 104 GWASs. Here we used *N* = 76 GWASs (73% of the holdout set) as this is the same size as the leaderboard set, but this choice does not critically affect results. Note that we have to do subsampling rather than resampling of GWASs because the scoring counts the number of modules that are associated to at least one GWAS; that is, including the same GWASs multiple times does not affect the score. We applied this approach to create 1,000 subsamples of the holdout set. The methods were then scored on each subsample.

The performance of every method *m* was compared to the highest-scoring method across the subsamples by the paired Bayes factor *K*_*m*_. That is, the method with the highest overall score in the holdout set (all 104 GWASs) was defined as reference (that is, method K1 in Sub-challenge 1). The score *S(m, k)* of method *m* in subsample *k* was thus compared with the score *S(ref, k)* of the reference method in the same subsample *k*. The Bayes factor *K*_*m*_ is defined as the number of times the reference method outperforms method *m*, divided by the number of times method *m* outperforms or ties the reference method over all subsamples. Methods with *K*_*m*_ < 3 were considered a tie with the reference method (that is, method *m* outperforms the reference in more than one out of four subsamples).

### Overview of module identification methods in Sub-challenge 1

Based on descriptions provided by participants, module identification methods were classified into different categories (Fig. 2a). Categories and corresponding module identification methods are summarized in Supplementary Table 2. In the following, we first give an overview of the different categories and top-performing methods, and then describe common pre- and postprocessing steps used by these methods:

- Kernel clustering: instead of working directly on the networks themselves, these methods cluster a kernel matrix, where each entry (*i*, *j*) of that matrix represents the closeness of nodes *i* and *j* in the network according to the particular similarity function, or kernel that was applied. Some of the kernels that were applied are well-known for community detection, such as the exponential diffusion kernel based on the graph Laplacian^47^ employed by method K6. Others, such as the LINE embedding algorithm^48^ employed by method K3 and the kernel based on the inverse of the weighted diffusion state distance^24,25^ employed by method K1, were newer. Method K1 was the best-performing method of the challenge and is described in detail below.
- Modularity optimization: this method category was, along with random-walk-based methods, the most popular type of method contributed by the community. Modularity optimization methods use search algorithms to find a partition of the network that maximizes the modularity *Q* (commonly defined as the fraction of within-module edges minus the expected fraction of such edges in a random network with the same node degrees)^43^. The most popular algorithm was Louvain community detection^49^. At least eight teams employed this algorithm in some form as either their main method or one of several methods, including the fourth ranking team. The best-performing modularity optimization method (M1), which ranked second overall, is described in detail below.
- Random-walk-based methods: these methods take inspiration from random walks or diffusion processes over the network. Several teams used the established Walktrap^50^, Infomap^51^ and Markov clustering algorithms. The top team of this category (method R1, third rank overall) used a sophisticated random-walk method based on multi-level Markov clustering^28^, which is described in detail below. While we did not include kernel methods in the ‘random walk’ category, several of the successful kernel clustering methods used random-walk-based measures within their kernel functions.
- Local methods: only three teams used local community detection methods, including agglomerative clustering and seed set expansion approaches. The top team of this category (method L1, fifth rank overall) first converted the adjacency matrix into a topology overlap matrix^35^, which measures the similarity of nodes based on the number of neighbors that they have in common. The team then used the SPICi algorithm^52^, which iteratively adds adjacent genes to cluster seeds such as to improve their local density.
- Hybrid methods: seven teams employed hybrid methods that leveraged clusterings produced by several of the different main approaches listed above. These teams applied more than one community detection method to each network to get larger and more diverse sets of predicted modules. The most common methods applied were Louvain^49^, hierarchical clustering and Infomap^51^. Two different strategies were used to select a final set of modules for submission: (1) choose a single method for each network according to performance in the leaderboard round, and (2) select modules from all applied methods according to a topological quality score such as the modularity or conductance^10^.
- Ensemble methods: much like hybrid methods, ensemble methods leverage clusterings obtained from multiple community detection methods (or multiple stochastic runs of a single method). However, instead of selecting individual modules according to a quality score, ensemble methods merge alternative clusterings to obtain potentially more robust consensus predictions^53^. Our method to derive consensus module predictions from team submissions is an example of an ensemble approach (see below).

Besides the choice of the community detection algorithm, there are other steps that critically affected performance, including preprocessing of the network data, setting of method parameters and postprocessing of predicted modules (Supplementary Table 2):

- Preprocessing: most networks in the challenge were densely connected, including many edges of low weight that are likely noisy. Some of the top teams (for example, M1, R1, L1) benefited from sparsifying these networks by discarding weak edges before applying their community detection methods. An added benefit of sparsification is that it typically reduces computation time. Few teams also normalized the edge weights of a given network to make them either normally distributed or fall in the range between zero and one. Not all methods required preprocessing of networks; for example, the top-performing method (K1) was applied to the original networks without any sparsification or normalization steps.
- Parameter setting: community detection methods often have parameters that need to be specified, typically to control the resolution of the clustering (the number and size of modules). While some methods have parameters that explicitly set the number of modules (for example, the top-performing method K1), other methods have parameters that indirectly control the resolution (for example, the resistance parameter of the runner-up method M1). While there were also methods that had no parameters to set (for example, the classic Louvain algorithm), these methods have an intrinsic resolution that may not always be optimal for a given network and target application.
- Postprocessing: modularization of biological networks often results in highly imbalanced module sizes. That is, some modules may be very small (for example, just one or two genes), while others are extremely large (for example, thousands of genes). Both extremes are generally not useful to gain biological insights at the pathway level. Since current community detection methods generally do not allow constraints on module size to be specified, teams used different postprocessing steps to deal with modules outside the allowed range in the challenge (between 3 and 100 genes). A successful strategy to break down large modules was to recursively apply community detection methods to each of these modules. Alternatively, all modules of invalid size were merged and the method was reapplied to the corresponding subnetwork. Finally, modules with fewer than three genes were often discarded. Some teams also discarded larger modules that were deemed low quality according to a topological metric, although this strategy was generally not beneficial.

### Method K1 (first rank in Sub-challenge 1)

The top-performing team developed a kernel clustering approach (method K1) based on a distance measure called diffusion state distance (DSD)^24,25^, which they further improved for this challenge. DSD produces a more informative notion of proximity than the typical shortest path metric, which measures distance between pairs of nodes by the number of hops on the shortest path that joins them in the network. More formally, consider the undirected network *G*(*V*, *E*) on the node set *V* = {*v*_1_, *v*_2_, *v*_3_,...,*v*_*n*_} with |*V*| = *n*. **He**^*t*^(*v*_*x*_, *v*_*y*_) is defined as the expected number of times that a random walk (visiting neighboring nodes in proportion to their edge weights) starting at node *v*_*x*_ and proceeding for some fixed *t* steps will visit node *v*_*y*_ (the walk includes the starting point, that is, 0th step). Taking a global view, we define the *n*-dimensional vector **He**^*t*^(*v*_*x*_) whose *i*th entry is the **He**^*t*^(*v*_*x*_,*v*_*i*_) value to network node *v*_*i*_. Then the DSD^*t*^ distance between two nodes *v*_*x*_ and *v*_*y*_ is defined as the *L1* norm of the difference of their **He**^*t*^ vectors, that is

$${\mathrm{DSD}}^t(v_{x},v_{y}) = ||{\mathbf{He}}^{t}(v_{x}) - {\mathbf{He}}^{t}(v_{y})||_{1}$$

It can be shown that DSD is a metric and converges as *t*→∞, allowing DSD to be defined independently from the value *t* (ref. ^24^.) The converged DSD matrix can be computed tractably, with an eigenvalue computation, as

$${\mathrm{DSD}}(v_x,v_y) = ||({\mathbf{1}}_{x} - {\mathbf{1}}_{y})(I - D^{ - 1}A + W)^{ - 1}||_1$$

where *D* is the diagonal degree matrix, *A* is the adjacency matrix and *W* is the matrix where each row is a copy of *π*, the degrees of each of the nodes, normalized by the sum of all the vertex degrees (in the unweighted case, weighted edges can be normalized proportional to their weight) and **1**_**x**_ and **1**_**y**_ are the vectors that are zero everywhere except at position *x* and *y*, respectively. The converged DSD matrix was approximated using algebraic multigrid techniques. Note that for the signaling network, edge directions were kept and low-weight back edges were added so that the network was strongly connected; that is, if there was a directed edge from *v*_*x*_ to *v*_*y*_, an edge from *v*_*y*_ to *v*_*x*_ of weight equal to 1/100 of the lowest edge weight in the network was added.

A spectral clustering algorithm^26^ was used to cluster the DSD matrix of a given network. Note that the spectral clustering algorithm operates on a similarity matrix (that is, entries that are most alike have higher values in the matrix). However, the DSD matrix is a distance matrix (that is, similar entries have low DSD values). The radial basis function kernel presents a standard way to convert the DSD matrix to a similarity matrix; it maps low distances to high similarity scores and vice versa. Since the spectral clustering algorithm employed uses *k*-means as the underlying clustering mechanism, it takes a parameter *k* specifying the number of cluster centers. The leaderboard rounds were used to measure the performance of different *k* values. Clusters with fewer than three nodes were discarded. Clusters with over 100 nodes were recursively split into two subclusters using spectral clustering (that is, *k* = 2) until all clusters had fewer than 100 nodes.

The top-performing team also used a different algorithm to search for dense bipartite subgraph module structure in half of the challenge networks and merged these modules (which were rare) with the clusters generated by their main method^54^. However, a post facto analysis of their results showed that this step contributed few modules and the score would have been similar with this additional procedure omitted.

### Method M1 (second rank in Sub-challenge 1)

The runner-up team developed a multi-resolution modularity optimization method^27^. The rationale is that in the absence of information on the cluster sizes of the graph, a method should be able to explore all possible topological scales at which clusters may satisfy the definition of module. The multi-resolution method developed by the team works by adding a resistance parameter *r* to the community detection algorithms. This resistance controls the aversion of nodes to form communities; the larger the resistance, the smaller the size of the modules. For community detection algorithms based on the optimization of the well-known modularity function^43^, this resistance takes the form of a self-loop (with a weight equal to *r*) which is added to all nodes of the network. In this way, all nodes contribute to the internal strength of their modules with a constant amount *r*. When the resistance is zero, the standard (and implicit) scale of resolution is recovered.

The team first sparsified networks by removing low confidence edges and then applied several well-known modularity optimization algorithms, including: (1) extremal optimization, (2) spectral optimization, (3) Newman’s fast algorithm and (4) fine-tuning by iterative repositioning of nodes. The idea is that a combination of several algorithms has fewer chances to get stacked in a suboptimal partition. The resistance parameter *r* was optimized so as to maximize the proportion of nodes inside communities of the desired sizes defined by the challenge rules, that is, between 3 and 100 nodes (only a handful of values were evaluated due to computational cost, but resulted in much better resolutions than the default of *r* = 0). Communities above the size limit (100 nodes) were subdivided recursively.

### Method R1 (third rank in Sub-challenge 1)

This team used balanced multi-layer regularized Markov clustering (bMLRMCL)^28^, an extension of the Markov cluster algorithm (MCL). The algorithm improves three common issues with MCL: (1) scalability for large graphs; (2) fragmented clusters due to the existence of hub nodes and (3) modules of imbalanced size.

Regularized MCL (RMCL) changes the MCL expansion step by introducing a canonical flow matrix, which ensures that the original topology of the graph still influences the graph clustering process beyond the first iteration. Multi-layer RMCL further improves the runtime by first coarsening the graph into multiple layers of smaller graphs to run RMCL on. Last, the balanced version of the algorithm computes a new regularization matrix at each iteration that penalizes big cluster sizes, where the penalty can be adjusted using a balance parameter^28^. Altogether, the method has three parameters: the inflation parameter *i*, coarsening size *c* and size balance parameter *b*. As preprocessing steps, the team first discarded weak edges and then transformed edge weights to integers. Communities with more than 100 nodes were recursively reclustered.

### Overview of module identification methods in Sub-challenge 2

There are broadly three different approaches to identify integrated modules across multiple networks: (1) the networks are first merged and then single-network module identification methods are applied on the integrated network, (2) single-network module identification methods are first applied on each individual network and then the resulting modules are merged across networks and (3) dedicated multi-network community detection methods are employed (also called multi-layer or multiplex methods), which are specifically designed to identify modules in layered networks^30,31^.

In Sub-challenge 2, the majority of teams employed the first approach. These teams built an integrated network by merging either all six or a subset of the challenge networks, and then applied single-network methods (typically the same method as in Sub-challenge 1) to modularize the integrated network. For example, the team with highest score in Sub-challenge 2 merged the two protein–protein interaction networks and then applied the Louvain algorithm to identify modules in the integrated network. The top-performing team from Sub-challenge 1 also performed competitively in Sub-challenge 2. They applied their single-network method (K1) to an integrated network consisting of the union of all edges from the two protein–protein interaction networks and the co-expression network.

Dedicated multi-network community detection methods were also employed by several teams^30,31^. For example, the runner-up team in Sub-challenge 2 previously extended the modularity measure to multiplex networks and adapted the Louvain algorithm to optimize this multiplex-modularity^31^. For this challenge, the team further improved their method with a randomization procedure, the consideration of edge and layer weights and a recursive clustering of the communities larger than a given size^36^.

Similar to Sub-challenge 1, teams used the leaderboard phase to set parameters of their methods. However, besides the parameters of the community detection method, there were additional choices to be made: whether to use all or only a subset of the six networks and how to integrate them.

None of the teams employed the second approach mentioned above, that is, to merge modules obtained from different networks. This approach was only used by the organizers, to form consensus modules in Sub-challenge 2 (see next section). In recent work, Sims et al. intersected brain-specific co-expression modules with generic protein–protein interaction networks, leading to a refined network module enriched for both common and rare variants associated with Alzheimer’s disease^55^. Exploring this type of approach with our challenge resources and potentially additional context-specific networks is thus an interesting avenue for future work.

### Consensus module predictions

We developed an ensemble approach to derive consensus modules from a given set of team submissions (see Supplementary Fig. 7 for a schematic overview). In Sub-challenge 1, a consensus matrix *C*^*n*^ was defined for each network *n*, where each element *c*_*ij*_ corresponds to the fraction of teams that put gene *i* and *j* together in the same module in this network. That is, *c*_*ij*_ equals one if all teams clustered gene *i* and *j* together, and *c*_*ij*_ equals zero if none of the teams clustered the two genes together. The top-performing module identification method (K1) was used to cluster the consensus matrix (that is, the consensus matrix was considered a weighted adjacency matrix defining a functional gene network, which was clustered using the top module identification method of the challenge). Method K1 has only one parameter to set, which is the number of cluster centers used by the spectral clustering algorithm. This parameter was set to the median number of modules submitted by the considered teams for the given network. The consensus module predictions described in the main text were derived from the submissions of the top 50% teams (that is, 21 teams) with the highest overall score on the leaderboard GWAS set.

Multi-network consensus modules were obtained by integrating team submissions from Sub-challenge 1 across all six networks using the same approach (Supplementary Fig. 7). The same set of teams was considered (that is, top 50% on the leaderboard GWAS set). First, a multi-network consensus matrix was obtained by taking the mean of the six network-specific consensus matrices *C*^*n*^. The multi-network consensus matrix was then clustered using method K1 as described above, where the number of cluster centers was set to the median number of modules submitted by the considered teams across all networks.

Two additional, more sophisticated approaches to construct consensus matrices *C*^*n*^ were tested: (1) normalization of the contribution of each module by the module size led to similar results as the basic approach described above, and (2) unsupervised estimation of module prediction accuracy using the Spectral Meta Learner ensemble method^56^. These methods did not perform well in this context (Supplementary Fig. 7).

### Similarity of module predictions

To define a similarity metric between module predictions from different methods, we represented module predictions as vectors. Namely, the set of modules predicted by method *m* in network *k* was represented as a prediction vector **P**_*mk*_ of length *N*_*k*_(*N*_*k*_ − 1)/2, where *N*_*k*_ is the number of genes in the network. Each element of this vector corresponds to a pair of genes and equals one if the two genes are in the same module and zero otherwise. Accordingly, for any two module predictions (method *m*_1_ applied to network *k*_1_, and method *m*_2_ applied to network *k*_2_), we calculated the distance as follows:

$$D(m_1k_1,m_2k_2) = 1 - {\frac{{{\mathbf{P}}_{m_1k_1},{\mathbf{P}}_{m_2k_2} > }}{{||{\mathbf{P}}_{m_1k_1}||_2||{\mathbf{P}}_{m_2k_2}||_2}}}$$

where <.,.> is the Euclidean inner product, ||.||_2_ is the Euclidean norm and *D* is the (symmetric) distance matrix between the 252 module predictions submitted in Sub-challenge 1 (that is, 42 methods applied to each of six networks). The distance matrix *D* was used as input to multidimensional scaling analysis for dimensionality reduction in Fig. 3a.

### Overlap between trait-associated modules

Three different metrics were considered to quantify the overlap between trait-associated modules from different methods and networks. The first metric was the Jaccard index, which is defined as the size of the intersection divided by the size of the union of two modules (gene sets) *A* and *B*:

$$J(A,B) = \frac{{\left| {A \cap B} \right|}}{{\left| {A \cup B} \right|}}$$

The Jaccard index measures how similar two modules are, but does allow the detection of submodules. For example, consider a module *A* of size ten that is a submodule of a module *B* of size 100. In this case, even though 100% of genes of the first module are comprised in the second module, the Jaccard index is rather low (0.1). To capture submodules, we thus considered in addition the percentage of genes of the first module that are comprised in the second module:

$$S(A,B) = \frac{{A \cap B}}{{\left| A \right|}}$$

Last, we also evaluated the significance of the overlap. To this end, we computed the *P* value *p*_*AB*_ for the overlap between the two modules using the hypergeometric distribution. *P* values were adjusted using Bonferroni correction given the number of module pairs tested.

Based on these three metrics, we categorized the type of overlap that a given trait-module *A* had with another trait-module *B* as:

1. strong overlap if *J*(*A*,*B*) ≥ 0.5 and *p*_*AB*_ < 0.05;
2. submodule if *J*(*A*,*B*) <0 .5 and *S*(*A*,*B*) −*J* (*A*,*B*) ≥ 0.5 and *p*_*AB*_< 0.05;
3. partial overlap if *J*(*A*,*B*) < 0.5 and *S*(*A*,*B*) − *J*(*A*,*B*) < 0.5 and *p*_*AB*_ < 0.05;
4. insignificant overlap if *p*_*AB*_ ≥ 0.05.

An additional category, strong overlap and submodule, was defined for trait module *A* that satisfy both conditions (1) and (2) with two different trait modules *B* and *C*. This categorization was used to get a sense of the type of overlap between trait modules from all methods (see Supplementary Fig. 2).

### Trait similarity network

We defined a network level similarity between GWAS traits based on overlap between trait-associated modules. To this end, we only considered the most relevant networks for our collection of GWAS traits, that is, the two protein–protein interaction, the signaling and the co-expression network (see Fig. 2d). For a given network, the set of ‘trait-module genes’ *G*_*T*_ was obtained for every trait *T* by taking the union of the modules associated with that trait across all challenge methods. If different GWASs were available for the same trait type (see Supplementary Table 1), the union of all corresponding trait-associated modules was taken. The overlap between every pair of trait-module gene sets $G_{T_1}$ and $G_{T_2}$ was evaluated using the Jaccard index $J(G_{T_1}\! ,G_{T_2})$ and the hypergeometric *P* value $P_{T_1T_2}$ as described in the previous section. *P* values were adjusted using Bonferroni correction. For the visualization as a trait–trait network in Fig. 4c, an edge between traits *T*_1_ and *T*_2_ was added if the overlap was significant ($P_{T_1T_2} < 0.05$) in at least three out of the four considered networks, and node sizes and edge weights were set to be proportional to the average number of trait-module genes and the average Jaccard index across the four networks, respectively.

### Functional enrichment analysis

To test network modules for enrichment in known gene functions and pathways, we considered diverse annotation and pathway databases. GO annotations for biological process, cellular component and molecular functions were downloaded from the GO website (http://geneontology.org, accessed on 20 January 2017). Curated pathways (KEGG, Reactome and BioCarta) were obtained from MSigDB v.5.2 (http://software.broadinstitute.org/gsea). We also created a collection of gene sets reflecting mouse mutant phenotypes, as defined by the Mammalian Phenotype Ontology^57^. We started with data files HMD_HumanPhenotype.rpt and MGI_GenePheno.rpt, downloaded from the Mouse Genome Informatics database (http://www.informatics.jax.org) on 21 February 2016. The first file contains human–mouse orthology data and some phenotypic information; we then integrated more phenotypic data from the second file, removing the two normal phenotypes MP:0002169 (no abnormal phenotype detected) and MP:0002873 (normal phenotype). For each remaining phenotype, we then built a list of all genes having at least one mutant strain exhibiting that phenotype, which we considered as a functional gene set.

Annotations from curated databases are known to be biased toward certain classes of genes. For example, some genes have been much more heavily studied than others and thus tend to have more annotations assigned to them. This and other biases lead to an uneven distribution of the number of annotations per genes (annotation bias). On the other hand, the gene sets (modules) tested for enrichment in these databases typically also exhibit bias for certain classes of genes (selection bias)^58,59^. Standard methods for GO enrichment analysis use the hypergeometric distribution (that is, Fisher’s exact test), the underlying assumption being that, under the null hypothesis, each gene is equally likely to be included in the gene set (module). Due to selection bias, this is typically not the case in practice, leading to inflation of *P* values^58,59^. Following Young et al.^59^, we thus used the Wallenius non-central hypergeometric distribution to account for biased sampling. Corresponding enrichment *P* values were computed for all network modules and annotation terms (pathways). The genes of the given network were used as a background gene set. For each network, module identification method and annotation database, the *M* × *T* nominal *P* values of the *M* modules and *T* annotation terms were adjusted using the Bonferroni correction.

### Selection of representative module annotations

Gene-set enrichment analysis methods often identify multiple significantly enriched gene sets with very similar compositions. To annotate our modules with few, but informative, gene sets, we formulate the gene-set enrichment problem within a regression framework^33^. Thus, the problem of gene-set enrichment is transformed into a feature selection problem; that is, the aim is to select the gene sets that best predict the membership of genes in a given module.

We constructed gene sets from the latest version of GO (format v.1.2; data version, releases/15 July 2018) and the Reactome database (download time, 16 July 2018). Genes belonging to a GO term or a Reactome pathway are considered as one gene set, independent of positions of either the term or the pathway in the respective hierarchies. Next, we used the gene sets to construct a gene-by-gene-set binary matrix *G*, whose rows are genes and columns are gene sets. *G*_*ij*_ equals 1 if and only if gene *i* belongs to gene set *j*; otherwise *G*_*ij*_ equals zero.

Given a module *M*, as well as the background genes *B* (the union of genes within GO and Reactome), we construct a vector **y** representing all genes in *B*. We assign *y*_*i*_ = 1 if and only if gene *g*_*i*_ belongs to the module *M*. Next, we train the regression model: **y** = *G***β** + **ε** using elastic net with *α* = 0.5 (the hyperparameter *α* controls the number of selected gene sets). Gene sets with coefficients larger than zero were selected as representative annotations for the given module^33^.

### Reporting Summary

Further information on research design is available in the Nature Research Reporting Summary linked to this article.

## Online content

Any methods, additional references, Nature Research reporting summaries, source data, statements of code and data availability and associated accession codes are available at 10.1038/s41592-019-0509-5.

## Supplementary Information

### Integrated supplementary information

Supplementary Figure 1 Scores in Sub-challenge 1. **(a)** Overall scores of the 42 module identification methods applied in Sub-challenge 1 at four different FDR cutoffs (10%, 5%, 2.5%, and 1% FDR). For explanation see legend of Fig. 2b, which shows the scores at 5% FDR (the predefined cutoff used for the challenge ranking). The top-performing method (K1) ranks first at all four cutoffs. The consensus prediction achieves the top score at 10% and 5% FDR, but not at the more stringent cutoffs. **(b)** Average number of trait-associated modules across the 42 methods for each of the six networks. The most trait modules are found in the two protein-protein interaction (PPI) and the co-expression networks. Related to Fig. 2d, which shows the average number of trait modules relative to network size.

Supplementary Figure 2 Pairwise similarity of module predictions from different methods. **(a)** Pairwise similarity of module predictions from different methods in Sub-challenge 1, averaged over all networks. Similarity was computed based on whether the same genes were clustered together by the two methods. Specifically, a prediction vector ***P***_*mk*_ was defined for every method *m* and network *k*, specifying for every pair of genes whether they were co-clustered in the same module (Methods). The prediction vectors ***P***_*mk*_ of method *m* for the six networks (*k* = 1,2,...,6) were then concatenated, forming a single vector ***P***_*m*_ representing the module predictions of that method for all six networks. A corresponding distance matrix between the 42 methods was computed as described in Methods (Equation 1) and hierarchically clustered using Ward’s method. The annotation row and column show the method type. The top five methods (1-5) and the consensus (C) are highlighted. The top methods did not converge to similar module predictions (they are not all grouped together in the hierarchical clustering). Related to Fig. 3, which shows similarity of module predictions from individual networks. **(b)** Comparison of trait-associated modules identified by all challenge methods. Pie-charts show the percentage of trait modules that show overlap with at least one trait module from a different method in the same network (top) and in different networks (bottom). We distinguish between strong overlap, sub-modules, weak but statistically significant overlap, and insignificant overlap (Methods).

Supplementary Figure 3 Optimal module granularity is method- and network-specific. All panels show results for single-network module identification methods (Sub-challenge 1). **(a)** Average module size versus score for each of the 42 methods. The x-axis shows the average module size of a given method across the six networks. The y-axis shows the overall score of the method. Top teams (highlighted) produced modules of varying size, i.e., they did not converge to a similar module size during the leaderboard round. There is no significant correlation between module size and score (p-value = 0.13 using two-sided Pearson’s correlation test), i.e., the scoring metric did not generally favor either small or large modules. Rather, when optimizing parameters during the leaderboard round, teams converged to very different granularities that led to the best performance for their specific methods. **(b)** Average number of modules versus score for each method. The x-axis shows the average number of submitted modules across networks for a given method, and the y-axis shows the corresponding score. The top five teams (highlighted) submitted a variable number of modules (between 103 and 470 modules, on average, per network). There is no significant correlation between the number of submitted modules and the obtained score (p-value = 0.99 using two-sided Pearson’s correlation test), i.e., the scoring metric was not biased to generally favor either a small or high number of submitted modules. **(c)** Comparison of module sizes between networks and method types. For each network, boxplots show the distribution of average module sizes for kernel clustering (n = 6 methods), modularity optimization (n = 10 methods), random-walk-based (n = 10 methods), and hybrid methods (n = 7 methods; the remaining categories are not shown because they comprise only three methods each). Note that teams tuned the resolution (average module size) of their method during the leaderboard round. The variation in module size between different method categories and networks suggests that the optimal resolution is method- and network-specific. For example, teams using random-walk-based methods tended to choose a higher resolution (smaller average module size) than teams using kernel clustering or modularity optimization methods. On average, modules were smallest in the signaling network and largest in the co-expression network. **(d)** Module size versus trait-association p-value for individual modules from all methods and networks. For all n = 84,798 modules, the module size (x-axis) is plotted against the -log10 of the minimum Pascal p-value across all GWASs (y-axis). Color shows the density of points. By design, Pascal p-values are not confounded by module size^23^, which is confirmed here (the regression line, shown in red, is flat; see also Supplementary Fig. 4).

Supplementary Figure 4 Module granularity of random predictions does not correlate with score. The panels show the average number of trait-associated modules for 17 random modularizations of the networks (i.e., networks were decomposed into random modules of the given sizes). Results are shown both for Bonferroni (orange) and Benjamini-Hochberg (blue) corrected p-values at a significance level of 0.05. The difference between the two panels is the background gene set used for the Pascal module enrichment test (see Methods). **(a)** The complete set of all annotated genes is used as background to compute module enrichment (the UCSC known genes). This is an incorrect choice for the background because module genes are drawn from the network genes, which is a subset of all known genes. As expected, this incorrect choice of a background set leads to a higher number of trait-associated random modules than in Panel **b**, in particular for large modules. **(b)** The set of all genes in a given network is used as background to compute module enrichment. This is the approach that was employed for the challenge scoring. Besides from very small modules of size 3, the module size does not affect the number of trait-associated random modules, i.e., our scoring methodology is not biased towards a specific module size (see also Supplementary Fig. 3d).

Supplementary Figure 5 Scores in Sub-challenge 2. **(a)** Final scores of multi-network module identification methods in Sub-challenge 2 at four different FDR cutoffs (10%, 5%, 2.5%, and 1% FDR). For explanation see legend of Fig. 3e, which shows the scores at 5% FDR (the predefined cutoff used for the challenge ranking). Ranks are indicated for the top five teams (ties are broken according to robustness analysis described in **Panel b**). The multi-network consensus prediction (red) achieves the top score at each FDR cutoff. Interestingly, the performance of methods integrating all five networks (dark blue) seems to drop substantially at the more stringent FDR thresholds. For example, the second and third ranking methods at both 5% and 10% FDR, which integrated all five networks, performed poorly at the 2.5% and 1% FDR thresholds (see second and third row from the top). This suggests that not only the absolute number of trait-associated modules, but also their quality in terms of association strength could not be improved by considering multiple networks. As mentioned in the Discussion, the challenge networks may not have been sufficiently related for multi-network methods to reveal meaningful modules spanning several networks. Indeed, the similarity between our networks in terms of edge overlap was small (Supplementary Fig. 6). Of note, there is an important conceptual difference between the multi-network methods that teams applied (blue) and the multi-network consensus prediction (red). While the former performed modularization on blended or multi-layer networks, the latter integrated the single-network module predictions obtained from each individual network (see Supplementary Fig. 7). Results thus suggest that our multi-network consensus approach is better suited than multi-layer module identification methods when network similarity is low. Exploring the performance of these different approaches when applied to networks of varying similarity is a promising avenue for future work. **(b)** Robustness of the overall ranking in Sub-challenge 2 was evaluated by subsampling the GWAS set used for evaluation 1,000 times. For each method, the resulting distribution of ranks is shown as a boxplot (using the 5% FDR cutoff for scoring). Related to Fig. 2c, which shows the same analysis for Sub-challenge 1. The difference between the top single-network module prediction and the top multi-network module predictions is not significant when sub-sampling the GWASs (Bayes factor < 3, see Methods section “Robustness analysis of challenge ranking”).

Supplementary Figure 6 Pairwise similarity of challenge networks. Pairwise similarity of challenge networks. The upper triangle of the matrix shows the percent of shared links (the Jaccard index multiplied by 100) and the lower triangle shows the fold-enrichment of shared links compared to the expected number of shared links at random. The two protein-protein interaction networks are the two most similar networks, yet they have only 8% shared edges. Of note, a recent study has found similarly low overlap between protein-protein interaction networks from different sources, suggesting that these molecular maps are still far from complete^60^. 60. Huang, J. K. *et al*. Systematic Evaluation of Molecular Networks for Discovery of Disease Genes. *Cell Syst*. **6**, 484-495.e5 (2018)

Supplementary Figure 7Consensus Module Predictions.**(a)** Schematic of the approach used to generate single-network consensus module predictions for Sub-challenge 1. For each network, module predictions from the top 50% of teams were integrated in a consensus matrix *C*, where each element *c*_*ij*_ gives the fraction of teams that clustered gene *i* and *j* together in the same module in the given network (performance as the percentage of considered teams is varied is shown in (**c**)). The overall score from the leaderboard round was used to select the top 50% of teams, i.e., the same set of teams was used for each network. The consensus matrix of each network was then clustered using the top-performing module identification method of the challenge (method K1; see Methods). **(b)** The approach used to generate multi-network consensus module predictions for Sub-challenge 2 was exactly the same as for single-network predictions, except that team submissions from all networks were integrated in the consensus matrix *C*. In other words, as input we still used the single-network predictions of the top 50% of teams from Sub-challenge 1, but instead of forming a consensus matrix for each network, a single cross-network consensus matrix was formed. This cross-network consensus matrix is then clustered using method K1 as described above (see Methods). **(c)** Scores of the single-network consensus predictions as the percentage of integrated teams is varied. We considered the top 25%, 50%, 75% and 100% of teams, as well as the top eight (19%) teams (these are the teams that ranked 2nd, or tied with the team that ranked 2nd, at any of the considered FDR cutoffs). **(d)** Performance of different methods to construct the consensus matrix *C*. In addition to the basic approach described above (*Standard*), two more sophisticated approaches to construct the consensus matrix were evaluated (*Normalized* and *SML*). In each case, the same set of team submissions were integrated (top 50%) and method K1 was applied to cluster the resulting consensus matrix. The first alternative (*Normalized*) is similar to the basic method but further assumes that appearing together in a smaller cluster is stronger evidence that a pair of genes is associated than appearing together in a larger cluster. Thus, each cluster’s contribution to the consensus matrix was normalized by the size of the cluster. Furthermore, we normalized the *ij*-entry of the consensus matrix by the number of methods that assigned gene *i* to a cluster, thus taking the presence of background genes into account. We found that the consensus still achieved the top score with these normalizations, but there was no improvement compared to the basic approach. The second method is a very different approach called Spectral Meta Learner (SML)^56^. SML is an unsupervised ensemble method designed for two-class classification problems. Briefly, it takes a matrix of predictions *P*, where each row corresponds to different samples being classified and the columns correspond to different methods. Accordingly, each matrix element *P*_*ij*_ is the class (0 or 1) assigned to sample *i* by method *j*. Under the assumption of conditional independence of methods given class labels, SML can estimate the balanced accuracy of each classifier in a totally unsupervised manner using only the prediction matrix *P*. The algorithm then uses this information to construct an ensemble classifier in which the contribution of each classifier is proportional to its estimated performance (balanced accuracy). The module identification problem is an unsupervised problem by its nature and we applied the SML algorithm as a new way for constructing consensus modules. For each method *m* and network *k*, we created a vector of prediction *P*_*mk*_, of size $N_{G_k}$by $N_{G_k}$, where $N_{G_k}$is the number genes in network as follows: *P*_*mk*_(*i*, *j*) = 1, *if method m puts genes I and j in the same module* (1) *P*_*mk*_(*i*,*j*) = 0, *otherwise*. For each network, we constructed the prediction matrix *P* with each column *P*_*m*_ defined as above. We then provided this matrix as input to the SML algorithm. The SML algorithm outputs a consensus matrix, which assigns a weight between each pair of genes. We found that SML did not perform well in the context of this challenge, likely because the underlying assumption of SML is that top-performing methods converge to similar predictions, which was not the case here (see Fig. 3 and Supplementary Fig. 2).

Supplementary Figure 8 Number of distinct trait-associated modules recovered by top methods. Number of distinct trait-associated modules recovered by the top *K* methods. Here, we did not form consensus modules. Instead, given the top *K* methods, we considered the set including all individual modules predicted by these methods and scored them with the same pipeline as used for the challenge submissions. We then evaluated how many “distinct” trait-associated modules were recovered by these methods. Distinct modules were defined as modules that do not show any significant overlap among each other. Overlap between pairs of modules was evaluated using the hypergeometric distribution and called significant at 5% FDR (Benjamini-Hochberg adjusted p-value < 0.05). From the set of trait-associated modules discovered by the top *K* methods, we thus derived the subset of distinct trait-associated modules (when several modules overlapped significantly, only the module with the most significant GWAS p-value was retained). Although the resulting scores (number of distinct trait-associated modules) cannot be directly compared with the challenge scores (because module predictions had to be strictly non-overlapping in the challenge), it is instructive to see how many distinct trait modules can be recovered when applying multiple methods. The stacked bars (colors) further show how many of the distinct trait modules are contributed by each method category. The number of distinct trait modules is not monotonically increasing as more methods are added because the larger sets of modules also increase the multiple testing burden of the GWAS scoring. The top four methods together discover 78 distinct trait-associated modules. Relatively little is gained by adding a higher number of methods.

Supplementary Figure 9 Functional Enrichment for Example Modules. Enrichment p-values for mouse mutant phenotypes, Reactome pathways and GO biological processes are shown for four example modules discussed in the main text. P-values were computed using the non-central hypergeometric distribution and adjusted using the Bonferroni method (Methods). Results for the remaining trait-associated modules from the consensus analysis in the STRING protein-protein interaction network are shown in Supplementary Fig. 12 and Supplementary Table 4. Functional enrichment analysis for additional pathway databases and modules from all methods and networks are available on the challenge website. **(a)** Module associated with height described in Fig. 5 (n = 25 genes). **(b)** Module associated with rheumatoid arthritis described in Fig. 6a (n = 25 genes). **(c)** Module associated with inflammatory bowel disease described in Fig. 6b (n = 42 genes). **(d)** Module associated with myocardial infarction described in Fig. 6c (n = 36 genes).

Supplementary Figure 10 Enrichment of trait-associated modules in curated gene sets from recent studies . Enrichment of trait-associated modules in six curated gene sets from three recent studies. The first two gene sets were taken from Marouli et al.^32^ and correspond to genes comprising height-associated ExomeChip variants (n = 475 genes) and genes known to be involved in skeletal growth disorders (n = 266 genes), respectively. The third gene set was taken from de Lange et al.^61^ and corresponds to genes causing monogenic immunodeficiency disorders (n = 316 genes). Lastly, three gene sets relevant for type 2 diabetes (T2D) were taken from Fuchsberger et al.^62^ and correspond to genes in literature-curated pathways that are believed to be linked to T2D (we distinguished between genes in cytokine signalling pathways [n = 384 genes] and other pathways [n = 390 genes]) and genes causing monogenic diabetes (n = 81 genes). We then considered corresponding GWAS traits in our hold-out set, namely height, all immune-related disorders, and T2D. We tested all modules associated with these GWAS traits for enrichment in these six external gene sets. Enrichment was tested using the hypergeometric distribution and p-values were adjusted to control FDR using the Benjamini-Hochberg method. The heatmap shows for each GWAS (row) the fraction of trait-associated modules that significantly overlap with a given gene set (column). It can be seen that modules associated with a given trait predominantly overlap the external gene sets that are expected to be relevant for that trait. 61. de Lange, K. M. *et al*. Genome-wide association study implicates immune activation of multiple integrin genes in inflammatory bowel disease. *Nat. Genet*. **49**, 256–261 (2017). 62. Fuchsberger, C. et al. The genetic architecture of type 2 diabetes. *Nature* **536**, 41–47 (2016).

Supplementary Figure 11 Support of trait-module genes in higher-powered GWASs. Trait-associated modules comprise many genes that show only borderline or no signal in the corresponding GWAS (called “candidate trait genes”). To assess whether modules correctly prioritized candidate trait genes, we considered eight traits for which older (lower-powered) and more recent (higher-powered) GWAS datasets were available in our holdout set. This allowed us to evaluate how well trait-associated modules and candidate trait genes predicted using the lower-powered GWAS datasets were supported in the higher-powered GWAS datasets. **(a)** Pairs of older (lower-powered) and more recent (higher-powered) GWASs used for the evaluation of module-based gene prioritization. The first column gives the trait and the second and third columns the corresponding GWASs. The bar plot shows the percentage of trait-associated modules from the first GWAS that are also trait-associated modules in the second GWAS. At the bottom, the expected percentage of confirmed modules at random is shown (i.e., assuming the trait-associated modules in the second GWAS were randomly selected from the set of predicted modules). **(b)** Height-associated module from Fig. 5 as an illustrative example (n = 25 genes). The module shows modest association to height in the lower-powered GWAS. Color indicates GWAS gene scores (FDR-corrected Pascal p-value = 0.04, see Methods). The signal is driven by three genes from different loci with significant scores (pink), while the remaining genes (grey) are predicted to be involved in height because of their module membership. **(c)** The module from (**b**) is supported in the higher-powered GWAS (*q*-value = 0.005). 45% of candidate trait genes (grey in (**b**)) are confirmed (pink). **(d)** Since high-powered GWASs typically result in many trait-associated genes, even random modules would have some genes “confirmed”. It is thus important to evaluate whether more candidate trait genes are confirmed than expected. Here we show support of candidate trait genes across the eight traits listed in (**a**). The lower-powered GWASs were used to predict candidate trait genes, defined as genes that: (*i*) are within a trait-associated module in the lower-powered GWAS; (*ii*) have a high gene p-value (*p* > 5E-4, i.e., two orders of magnitude above the genome-wide significance threshold of 5E-6 (cf. grey genes in (**a**)) and (*iii*) are located more than one megabase away from the nearest significant locus of the corresponding GWAS. Gene p-values were computed using Pascal as described above. Finally, the Pascal p-value of all candidate trait genes was evaluated for the higher-powered GWAS (n = 2,254 genes considering trait-modules from all methods). Since there is a genome-wide tendency for p-values to become more significant in higher-powered GWAS data^38^, Pascal p-values were also evaluated for a background gene set (all genes that meet the two conditions (*ii*, *iii*), but do not belong to trait-associated modules of the lower-powered GWAS). The plot shows the cumulative distribution of gene scores in the higher-powered GWASs for candidate trait genes (red line) and genes in the background set (grey line). a substantial fraction of module genes that do not show any signal and are located far from any significant locus in the lower-powered GWAS are subsequently confirmed by the higher-powered GWAS. **(e)** Since candidate trait genes (i.e., genes satisfying the three conditions (*i-iii*) described above) could still have lower p-values than genes in the background set (i.e., genes satisfying the two conditions (*ii*, *iii*)), we repeated the same analysis with higher gene p-value thresholds for condition (*ii*): p-value > 5E-3 (n = 2,185 genes) (**e**) and p-value > 5E-2 (n = 1,969 genes) (**f**). For this range the “discovery” gene score p-values in the candidate set and the background set are much more similar. Although there may remain some confounding, the same trend as in (**d**) is observed, indicating that the result is robust. This suggests that modules are predictive for trait-associated genes and could potentially be used to prioritize candidate genes for follow-up studies, for instance.

Supplementary Figure 12 Overview of Consensus Trait-modules in the STRING Network. Overview of all 21 trait-associated consensus modules in the STRING protein-protein interaction network. The first three columns give the module ID, the trait type, and the specific GWAS trait that the module is associated to. We tested all modules for enrichment in GO annotation, mouse mutant phenotypes, and other pathway databases using the non-central hypergeometric test (Methods). The putative function of each module based on this enrichment analysis is summarized in the fourth column (see Figs. 5 and 6, Supplementary Fig. 9, and Supplementary Table 4 for details). Two thirds of the modules have functions that correspond to core pathways underlying the respective traits, while the remaining modules correspond either to generic pathways that play a role in diverse traits or to pathways without an established connection to the considered trait or disease. Only pathways with a well-established link to the trait were considered core pathways. Generic pathways, such as cell-cycle-related or epigenetic pathways, were not considered core pathways because they are relevant for many traits and tissues, making them more difficult to target therapeutically. For example, modules 77 and 109 are both associated with schizophrenia and comprise pathways related to epigenetic gene silencing and nucleosome organization, respectively. Although there is evidence that epigenetic mechanisms may play a role in schizophrenia, we considered this to be a generic pathway.

Supplementary Figure 13 Modules Associated with IgA Nephropathy. The top ten enriched GO biological processes, Reactome pathways and mouse mutant phenotypes are shown for two IgA nephropathy (IgAN) associated modules. P-values were computed using the non-central hypergeometric distribution (Methods). **(a)** IgAN-associated module identified using the consensus analysis in the InWeb protein-protein interaction network (n = 19 genes). The module comprises immune-related NF-κB signaling pathways. Enriched mouse mutant phenotypes for module gene homologs include perturbed immunoglobulin levels (IgM and IgG1). The module implicates in particular the NF-κB subunit *REL* as a candidate gene. The *REL* locus does not reach genome-wide significance in current GWASs for IgAN but is known to be associated with other immune disorders such as rheumatoid arthritis. **(b)** IgAN-associated module identified by the best-performing method (K1) in the InWeb protein-protein interaction network (n = 12 genes). Besides finding complement factors that are known to play a role in the disease (*CFB* and *C4A*), the module implicates novel candidate genes such as the chemokine *Platelet Factor 4 Variant 1* (*PF4V1*) from a sub-threshold locus, and is enriched for coagulation cascade, a process known to be involved in kidney disease^62^. The top two enriched mouse mutant phenotypes are precisely “abnormal blood coagulation” and “glomerulonephritis”. 62. Madhusudhan, T., Kerlin, B. A. & Isermann, B. The emerging role of coagulation proteases in kidney disease. *Nat. Rev. Nephrol*. **12**, 94–109 (2016).

### Supplementary information

Supplementary Information Supplementary Figs. 1–13, and Supplementary Table 2.

Reporting Summary

Supplementary Table 1 Collection of GWAS Datasets used for the challenge. The table lists the GWAS datasets used for the module scoring. The first column indicates whether the GWAS was used during the leaderboard or final evaluation phase. The five GWAS listed in the end (extra) were not used for the scoring as they were added to the collection after the challenge. The Pascal gene scores for all GWAS are available for download from the challenge website (file names are given in the last column). The original GWAS SNP summary statistics can be downloaded individually from the indicated sources or we can share the complete collection upon request.

Supplementary Table 3 Challenge scores of methods in the leaderboard and final round. The table shows the challenge scores of all methods both for the leaderboard and final rounds.

Supplementary Table 4 Functional enrichment of consensus trait modules. For each of the 21 consensus trait-modules shown in Supplementary Fig. 12, all categories with a Bonferroni-corrected *P* *>* 0.05 are listed (Methods). Only results for mouse mutant phenotypes, Reactome pathways and GO biological process annotations are included for brevity. Full results including all tested pathway databases and all challenge modules are available on the challenge website.

## Data Availability

Challenge data and results are available from the challenge website (https://synapse.org/modulechallenge). This includes: official challenge rules; gene scores for the compendium of 180 GWASs used in the challenge plus five additional GWASs obtained after the challenge (GWAS SNP *P* values are available on request); official challenge rules; gene scores for the compendium of 180 GWASs used in the challenge plus five additional GWASs obtained after the challenge (GWAS SNP *P* values are available on request); the six challenge networks (anonymized and deanonymized versions); the final module predictions of all teams for both sub-challenges; consensus module predictions for both sub-challenges; individual module scores for all GWASs and enriched functional annotations for all modules.

## Code availability

Code is available on GitHub for: user-friendly, dockerized versions of the top three methods (https://github.com/BergmannLab/MONET); the latest Pascal version (https://www2.unil.ch/cbg/index.php?title=Pascal, https://github.com/dlampart/Pascal); the regression-based gene-set enrichment analysis (https://github.com/TaoDFang/GeneModuleAnnotation) and, in addition, the scoring scripts, a snapshot of the Pascal version used for the challenge, and module identification method descriptions and code provided by teams are available on the challenge website (https://synapse.org/modulechallenge).
