# Supplementary figures and images for: Assessment of network module identification across complex diseases
Source: Nat Methods. 2019 Aug 30;16(9):843–52. doi: 10.1038/s41592-019-0509-5 (PMC6719725; doi:10.1038/s41592-019-0509-5)

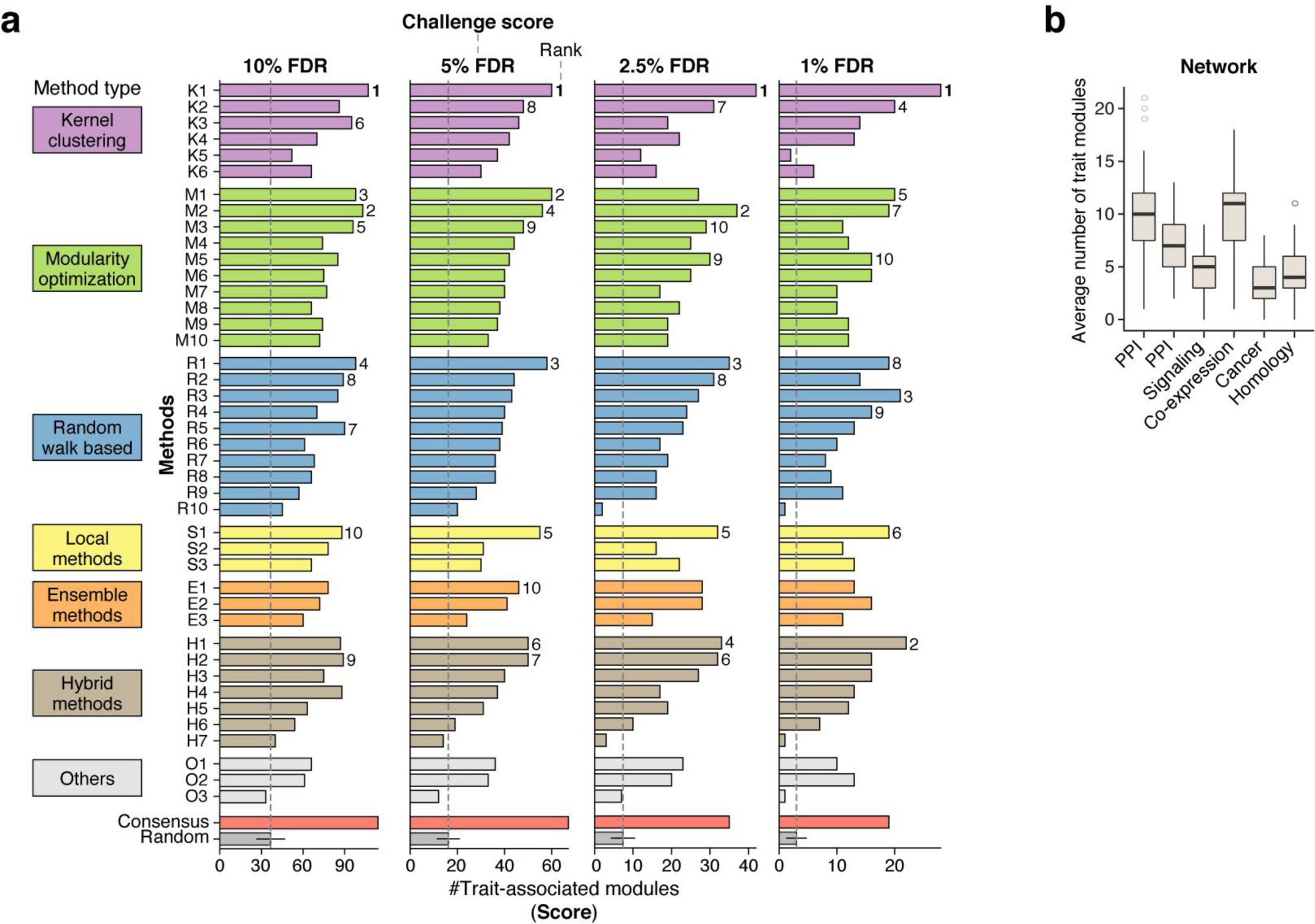

Supplement: Scores in Sub-challenge 1. — (a) Overall scores of the 42 module identification methods applied in Sub-challenge 1 at four different FDR cutoffs (10%, 5%, 2.5%, and 1% FDR). For explanation see legend of Fig. 2b, which shows the scores at 5% FDR (the predefined cutoff used for the challenge ranking). The top-performing method (K1) ranks first at all four cutoffs. The consensus prediction achieves the top score at 10% and 5% FDR, but not at the more stringent cutoffs. (b) Average number of trait-associated modules across the 42 methods for each of the six networks. The most trait modules are found in the two protein-protein interaction (PPI) and the co-expression networks. Related to Fig. 2d, which shows the average number of trait modules relative to network size. [file 41592_2019_509_Fig7_ESM.jpg]

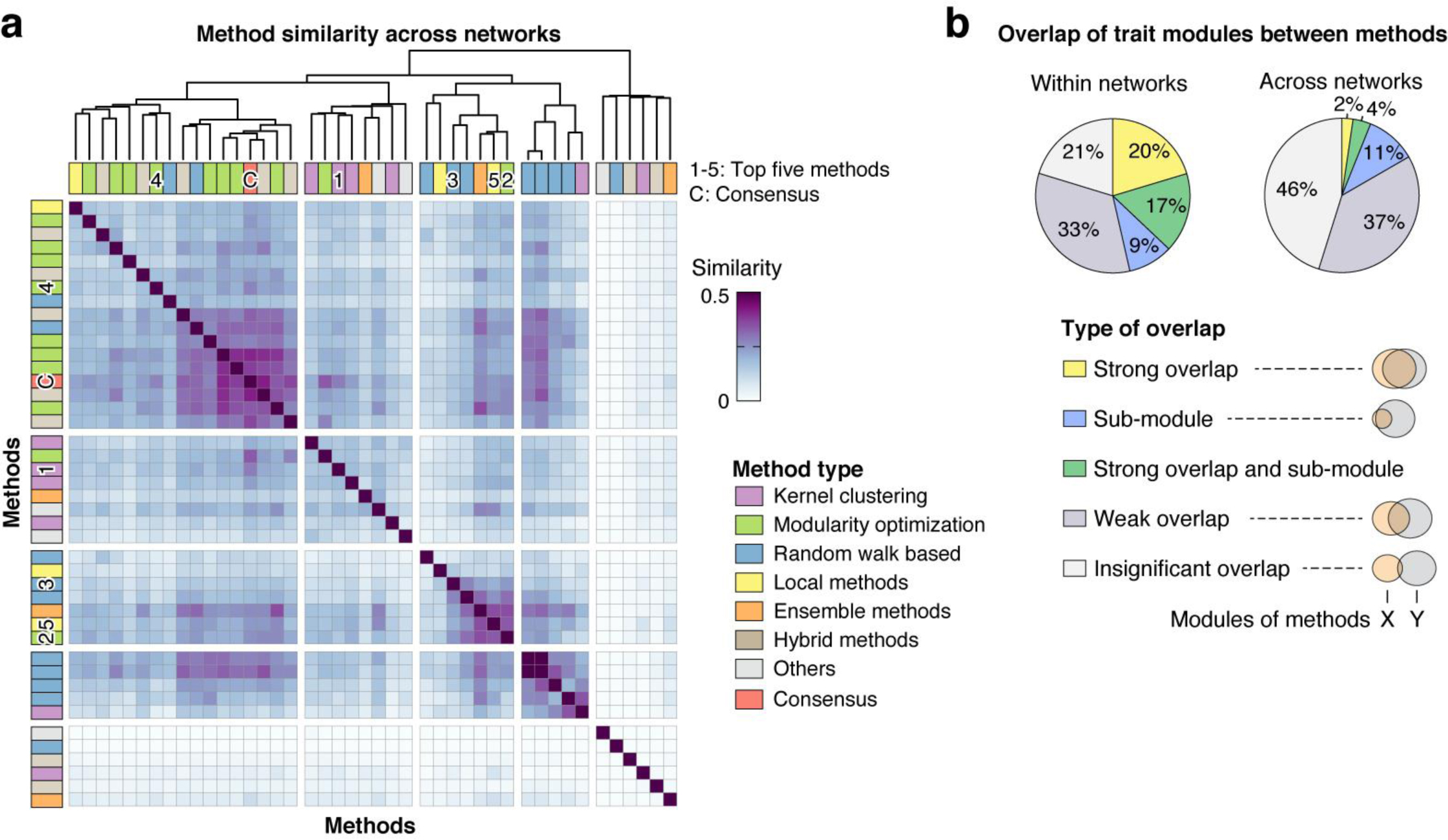

Supplement: Pairwise similarity of module predictions from different methods. — (a) Pairwise similarity of module predictions from different methods in Sub-challenge 1, averaged over all networks. Similarity was computed based on whether the same genes were clustered together by the two methods. Specifically, a prediction vector Pmk was defined for every method m and network k, specifying for every pair of genes whether they were co-clustered in the same module (Methods). The prediction vectors Pmk of method m for the six networks (k = 1,2,...,6) were then concatenated, forming a single vector Pm representing the module predictions of that method for all six networks. A corresponding distance matrix between the 42 methods was computed as described in Methods (Equation 1) and hierarchically clustered using Ward’s method. The annotation row and column show the method type. The top five methods (1-5) and the consensus (C) are highlighted. The top methods did not converge to similar module predictions (they are not all grouped together in the hierarchical clustering). Related to Fig. 3, which shows similarity of module predictions from individual networks. (b) Comparison of trait-associated modules identified by all challenge methods. Pie-charts show the percentage of trait modules that show overlap with at least one trait module from a different method in the same network (top) and in different networks (bottom). We distinguish between strong overlap, sub-modules, weak but statistically significant overlap, and insignificant overlap (Methods). [file 41592_2019_509_Fig8_ESM.jpg]

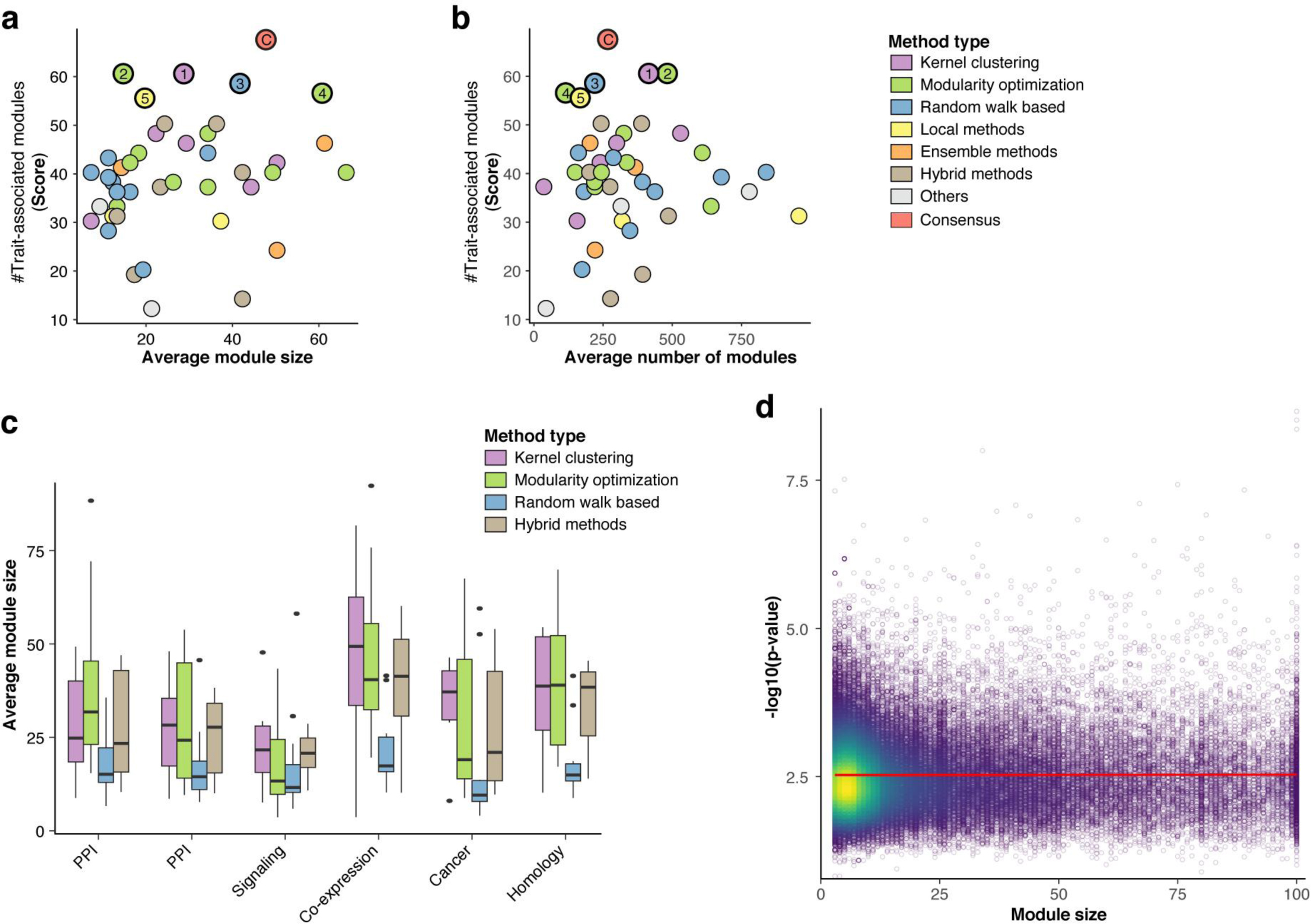

Supplement: Optimal module granularity is method- and network-specific. — All panels show results for single-network module identification methods (Sub-challenge 1). (a) Average module size versus score for each of the 42 methods. The x-axis shows the average module size of a given method across the six networks. The y-axis shows the overall score of the method. Top teams (highlighted) produced modules of varying size, i.e., they did not converge to a similar module size during the leaderboard round. There is no significant correlation between module size and score (p-value = 0.13 using two-sided Pearson’s correlation test), i.e., the scoring metric did not generally favor either small or large modules. Rather, when optimizing parameters during the leaderboard round, teams converged to very different granularities that led to the best performance for their specific methods. (b) Average number of modules versus score for each method. The x-axis shows the average number of submitted modules across networks for a given method, and the y-axis shows the corresponding score. The top five teams (highlighted) submitted a variable number of modules (between 103 and 470 modules, on average, per network). There is no significant correlation between the number of submitted modules and the obtained score (p-value = 0.99 using two-sided Pearson’s correlation test), i.e., the scoring metric was not biased to generally favor either a small or high number of submitted modules. (c) Comparison of module sizes between networks and method types. For each network, boxplots show the distribution of average module sizes for kernel clustering (n = 6 methods), modularity optimization (n = 10 methods), random-walk-based (n = 10 methods), and hybrid methods (n = 7 methods; the remaining categories are not shown because they comprise only three methods each). Note that teams tuned the resolution (average module size) of their method during the leaderboard round. The variation in module size between different method categories and networks suggests that the optimal re [file 41592_2019_509_Fig9_ESM.jpg]

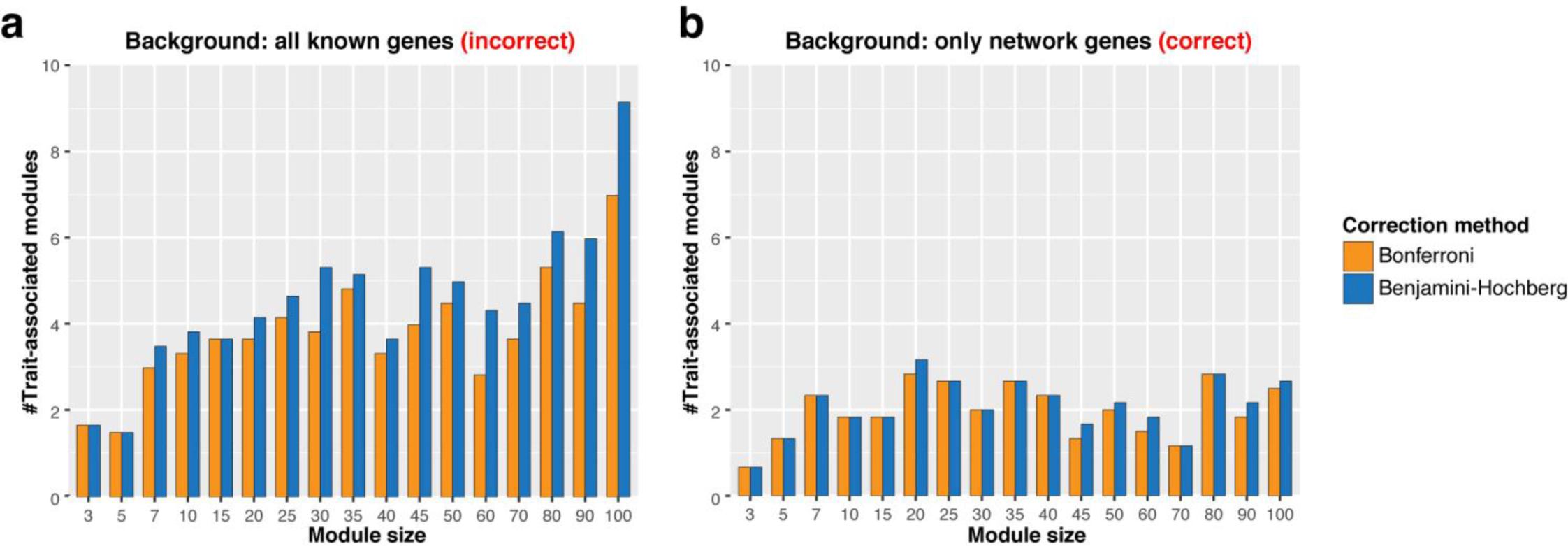

Supplement: Module granularity of random predictions does not correlate with score. — The panels show the average number of trait-associated modules for 17 random modularizations of the networks (i.e., networks were decomposed into random modules of the given sizes). Results are shown both for Bonferroni (orange) and Benjamini-Hochberg (blue) corrected p-values at a significance level of 0.05. The difference between the two panels is the background gene set used for the Pascal module enrichment test (see Methods). (a) The complete set of all annotated genes is used as background to compute module enrichment (the UCSC known genes). This is an incorrect choice for the background because module genes are drawn from the network genes, which is a subset of all known genes. As expected, this incorrect choice of a background set leads to a higher number of trait-associated random modules than in Panel b, in particular for large modules. (b) The set of all genes in a given network is used as background to compute module enrichment. This is the approach that was employed for the challenge scoring. Besides from very small modules of size 3, the module size does not affect the number of trait-associated random modules, i.e., our scoring methodology is not biased towards a specific module size (see also Supplementary Fig. 3d). [file 41592_2019_509_Fig10_ESM.jpg]

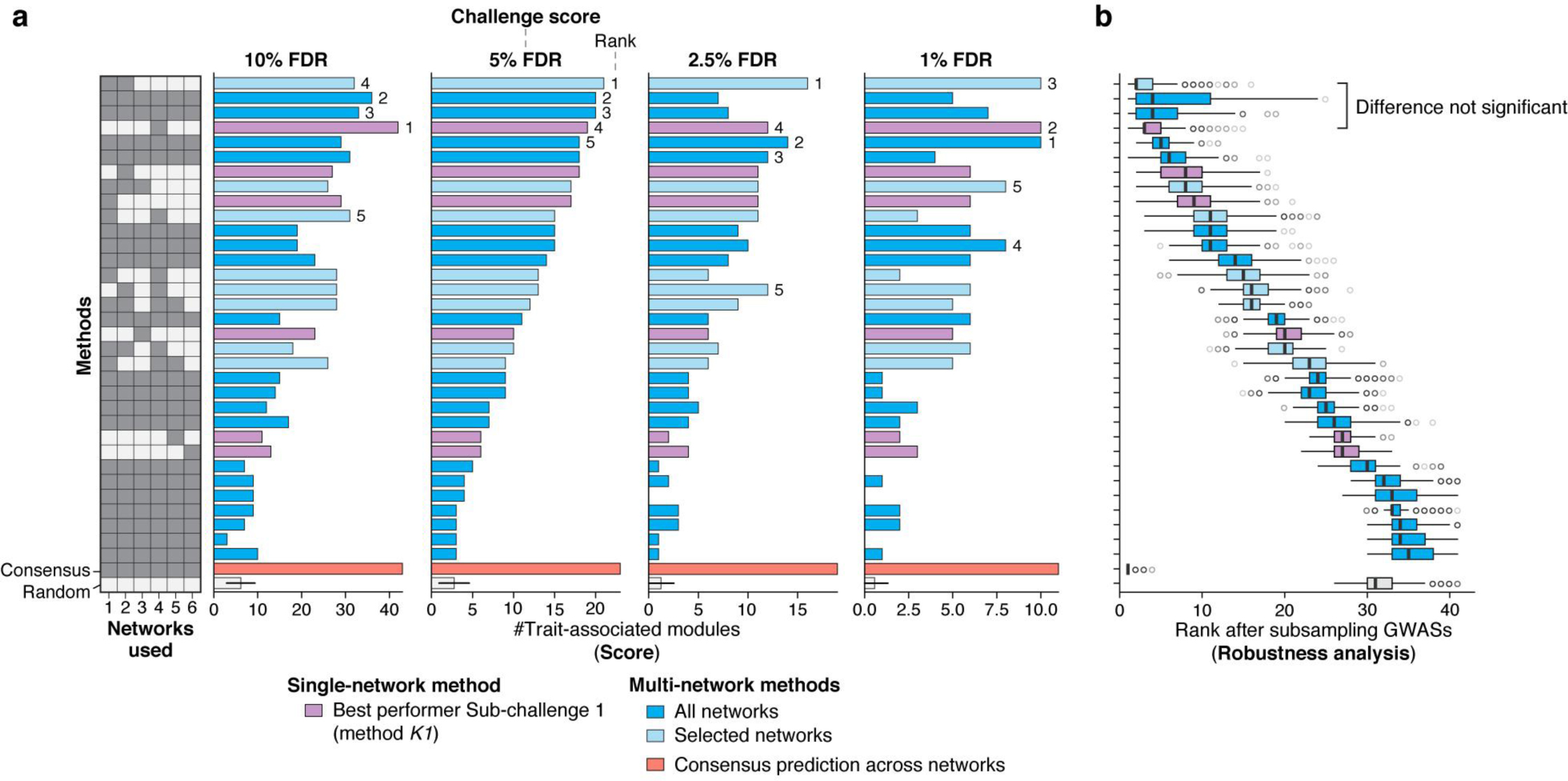

Supplement: Scores in Sub-challenge 2. — (a) Final scores of multi-network module identification methods in Sub-challenge 2 at four different FDR cutoffs (10%, 5%, 2.5%, and 1% FDR). For explanation see legend of Fig. 3e, which shows the scores at 5% FDR (the predefined cutoff used for the challenge ranking). Ranks are indicated for the top five teams (ties are broken according to robustness analysis described in Panel b). The multi-network consensus prediction (red) achieves the top score at each FDR cutoff. Interestingly, the performance of methods integrating all five networks (dark blue) seems to drop substantially at the more stringent FDR thresholds. For example, the second and third ranking methods at both 5% and 10% FDR, which integrated all five networks, performed poorly at the 2.5% and 1% FDR thresholds (see second and third row from the top). This suggests that not only the absolute number of trait-associated modules, but also their quality in terms of association strength could not be improved by considering multiple networks. As mentioned in the Discussion, the challenge networks may not have been sufficiently related for multi-network methods to reveal meaningful modules spanning several networks. Indeed, the similarity between our networks in terms of edge overlap was small (Supplementary Fig. 6). Of note, there is an important conceptual difference between the multi-network methods that teams applied (blue) and the multi-network consensus prediction (red). While the former performed modularization on blended or multi-layer networks, the latter integrated the single-network module predictions obtained from each individual network (see Supplementary Fig. 7). Results thus suggest that our multi-network consensus approach is better suited than multi-layer module identification methods when network similarity is low. Exploring the performance of these different approaches when applied to networks of varying similarity is a promising avenue for future work. (b) Robustness of the overall ranking [file 41592_2019_509_Fig11_ESM.jpg]

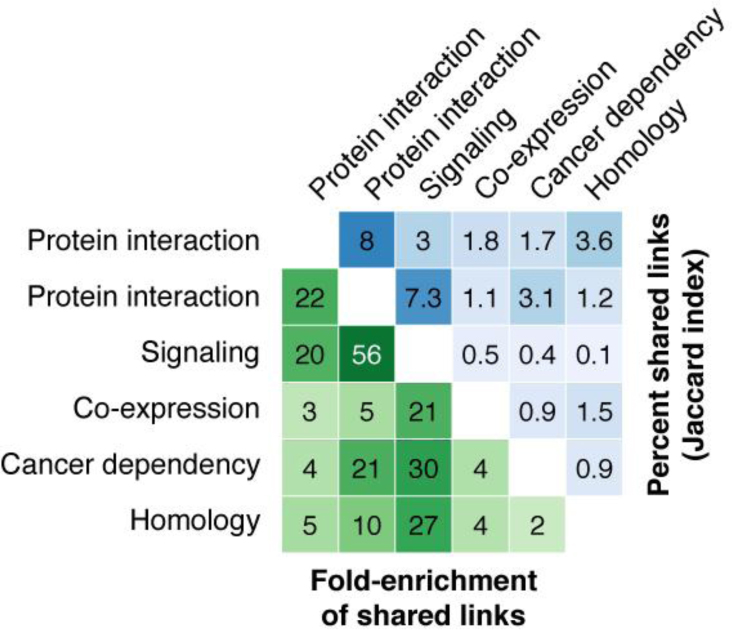

Supplement: Pairwise similarity of challenge networks. — The upper triangle of the matrix shows the percent of shared links (the Jaccard index multiplied by 100) and the lower triangle shows the fold-enrichment of shared links compared to the expected number of shared links at random. The two protein-protein interaction networks are the two most similar networks, yet they have only 8% shared edges. Of note, a recent study has found similarly low overlap between protein-protein interaction networks from different sources, suggesting that these molecular maps are still far from complete60. 60. Huang, J. K. et al. Systematic Evaluation of Molecular Networks for Discovery of Disease Genes. Cell Syst. 6, 484-495.e5 (2018) [file 41592_2019_509_Fig12_ESM.jpg]

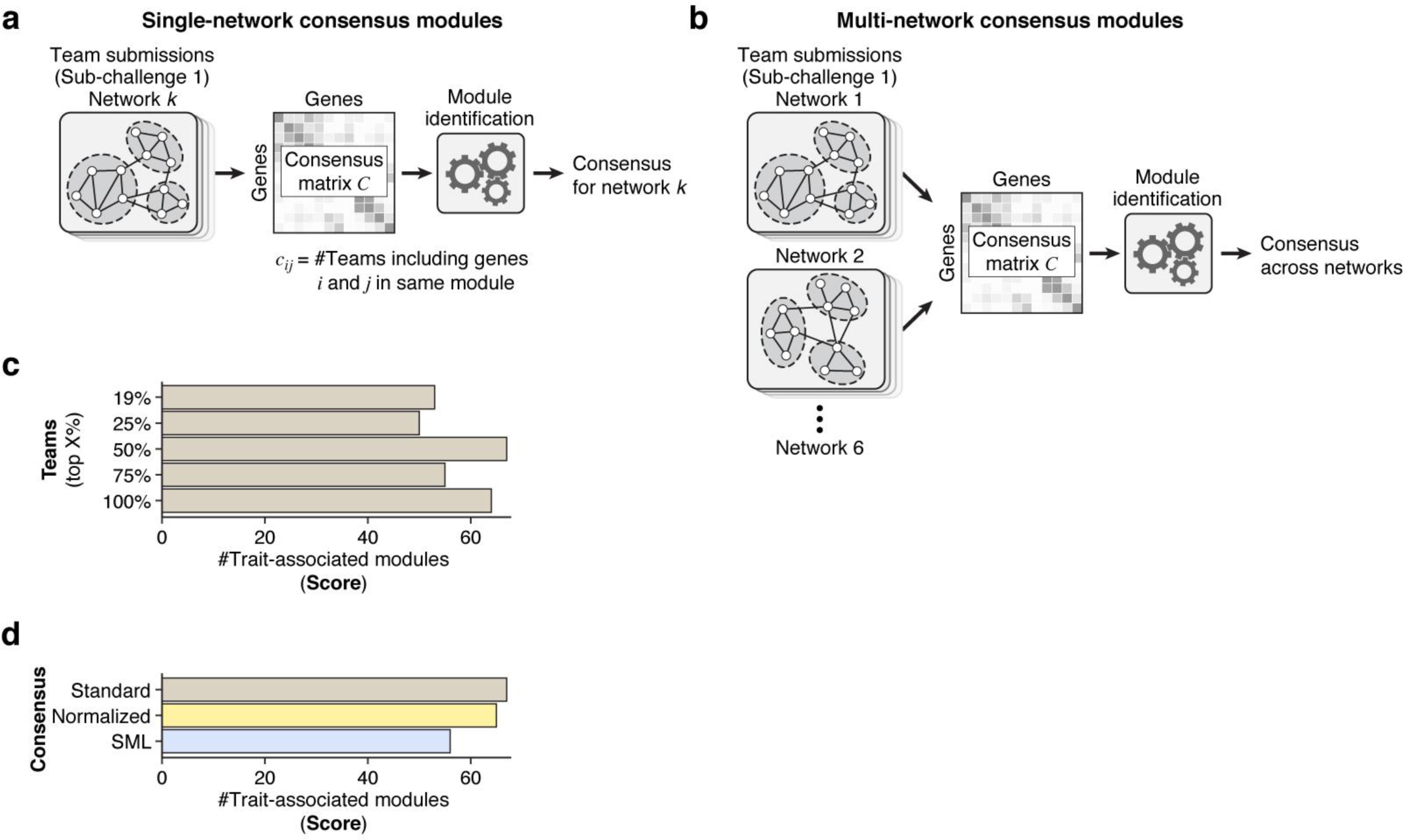

Supplement: Consensus Module Predictions. — (a) Schematic of the approach used to generate single-network consensus module predictions for Sub-challenge 1. For each network, module predictions from the top 50% of teams were integrated in a consensus matrix C, where each element cij gives the fraction of teams that clustered gene i and j together in the same module in the given network (performance as the percentage of considered teams is varied is shown in (c)). The overall score from the leaderboard round was used to select the top 50% of teams, i.e., the same set of teams was used for each network. The consensus matrix of each network was then clustered using the top-performing module identification method of the challenge (method K1; see Methods). (b) The approach used to generate multi-network consensus module predictions for Sub-challenge 2 was exactly the same as for single-network predictions, except that team submissions from all networks were integrated in the consensus matrix C. In other words, as input we still used the single-network predictions of the top 50% of teams from Sub-challenge 1, but instead of forming a consensus matrix for each network, a single cross-network consensus matrix was formed. This cross-network consensus matrix is then clustered using method K1 as described above (see Methods). (c) Scores of the single-network consensus predictions as the percentage of integrated teams is varied. We considered the top 25%, 50%, 75% and 100% of teams, as well as the top eight (19%) teams (these are the teams that ranked 2nd, or tied with the team that ranked 2nd, at any of the considered FDR cutoffs). (d) Performance of different methods to construct the consensus matrix C. In addition to the basic approach described above (Standard), two more sophisticated approaches to construct the consensus matrix were evaluated (Normalized and SML). In each case, the same set of team submissions were integrated (top 50%) and method K1 was applied to cluster the resulting consensus matrix. The first alt [file 41592_2019_509_Fig13_ESM.jpg]

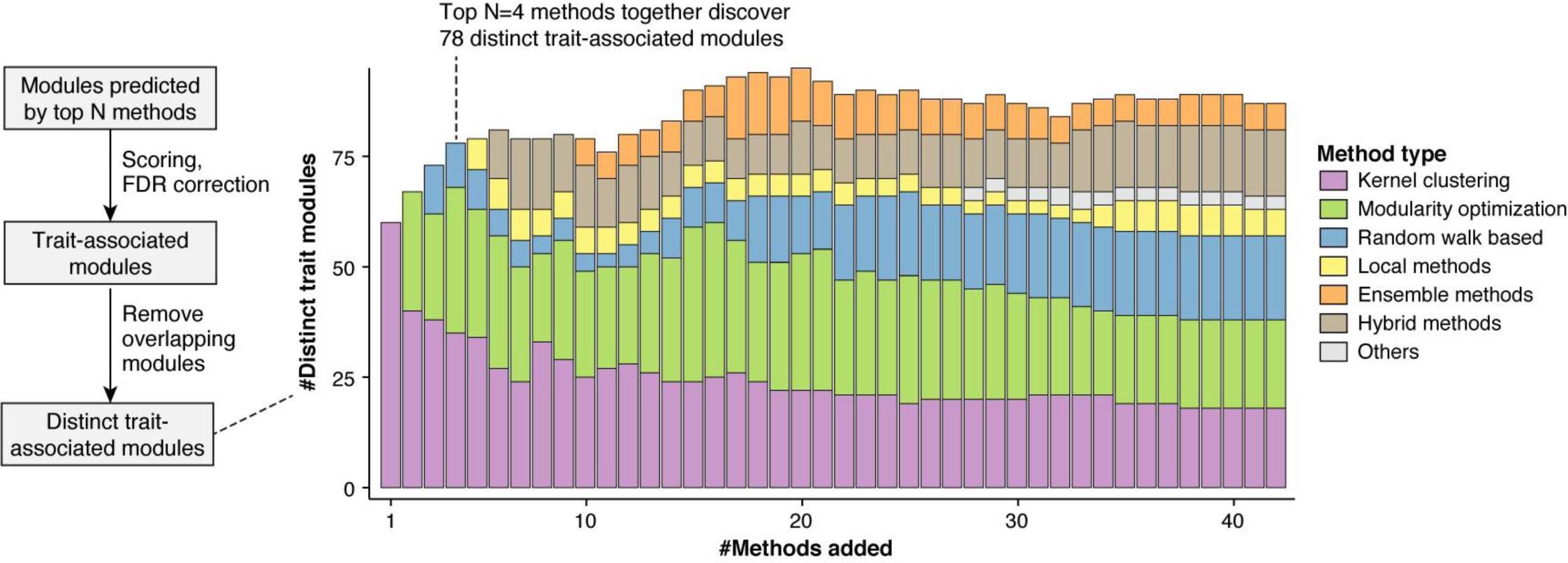

Supplement: Number of distinct trait-associated modules recovered by top methods. — Number of distinct trait-associated modules recovered by the top K methods. Here, we did not form consensus modules. Instead, given the top K methods, we considered the set including all individual modules predicted by these methods and scored them with the same pipeline as used for the challenge submissions. We then evaluated how many “distinct” trait-associated modules were recovered by these methods. Distinct modules were defined as modules that do not show any significant overlap among each other. Overlap between pairs of modules was evaluated using the hypergeometric distribution and called significant at 5% FDR (Benjamini-Hochberg adjusted p-value < 0.05). From the set of trait-associated modules discovered by the top K methods, we thus derived the subset of distinct trait-associated modules (when several modules overlapped significantly, only the module with the most significant GWAS p-value was retained). Although the resulting scores (number of distinct trait-associated modules) cannot be directly compared with the challenge scores (because module predictions had to be strictly non-overlapping in the challenge), it is instructive to see how many distinct trait modules can be recovered when applying multiple methods. The stacked bars (colors) further show how many of the distinct trait modules are contributed by each method category. The number of distinct trait modules is not monotonically increasing as more methods are added because the larger sets of modules also increase the multiple testing burden of the GWAS scoring. The top four methods together discover 78 distinct trait-associated modules. Relatively little is gained by adding a higher number of methods. [file 41592_2019_509_Fig14_ESM.jpg]

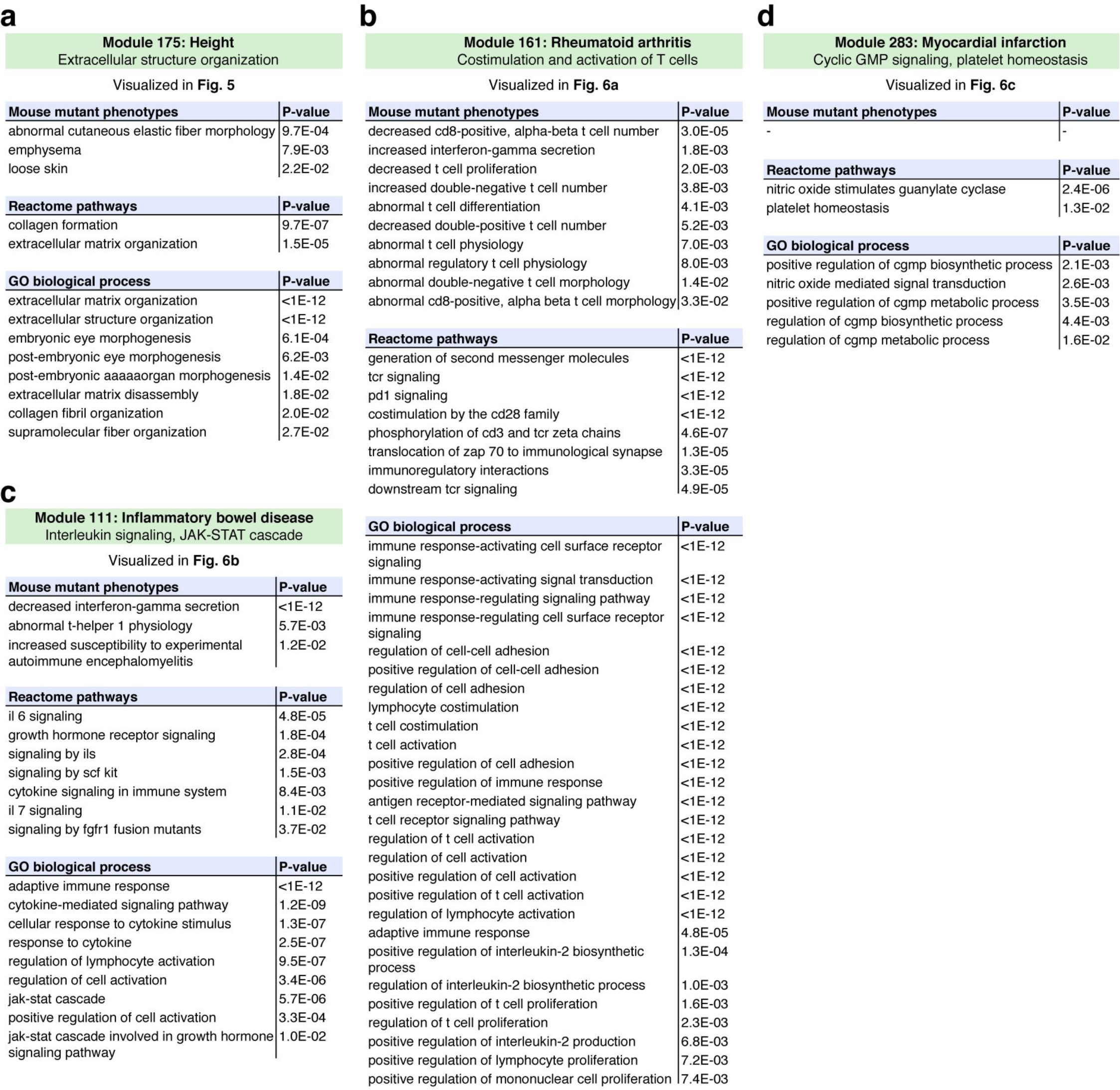

Supplement: Functional Enrichment for Example Modules. — Enrichment p-values for mouse mutant phenotypes, Reactome pathways and GO biological processes are shown for four example modules discussed in the main text. P-values were computed using the non-central hypergeometric distribution and adjusted using the Bonferroni method (Methods). Results for the remaining trait-associated modules from the consensus analysis in the STRING protein-protein interaction network are shown in Supplementary Fig. 12 and Supplementary Table 4. Functional enrichment analysis for additional pathway databases and modules from all methods and networks are available on the challenge website. (a) Module associated with height described in Fig. 5 (n = 25 genes). (b) Module associated with rheumatoid arthritis described in Fig. 6a (n = 25 genes). (c) Module associated with inflammatory bowel disease described in Fig. 6b (n = 42 genes). (d) Module associated with myocardial infarction described in Fig. 6c (n = 36 genes). [file 41592_2019_509_Fig15_ESM.jpg]

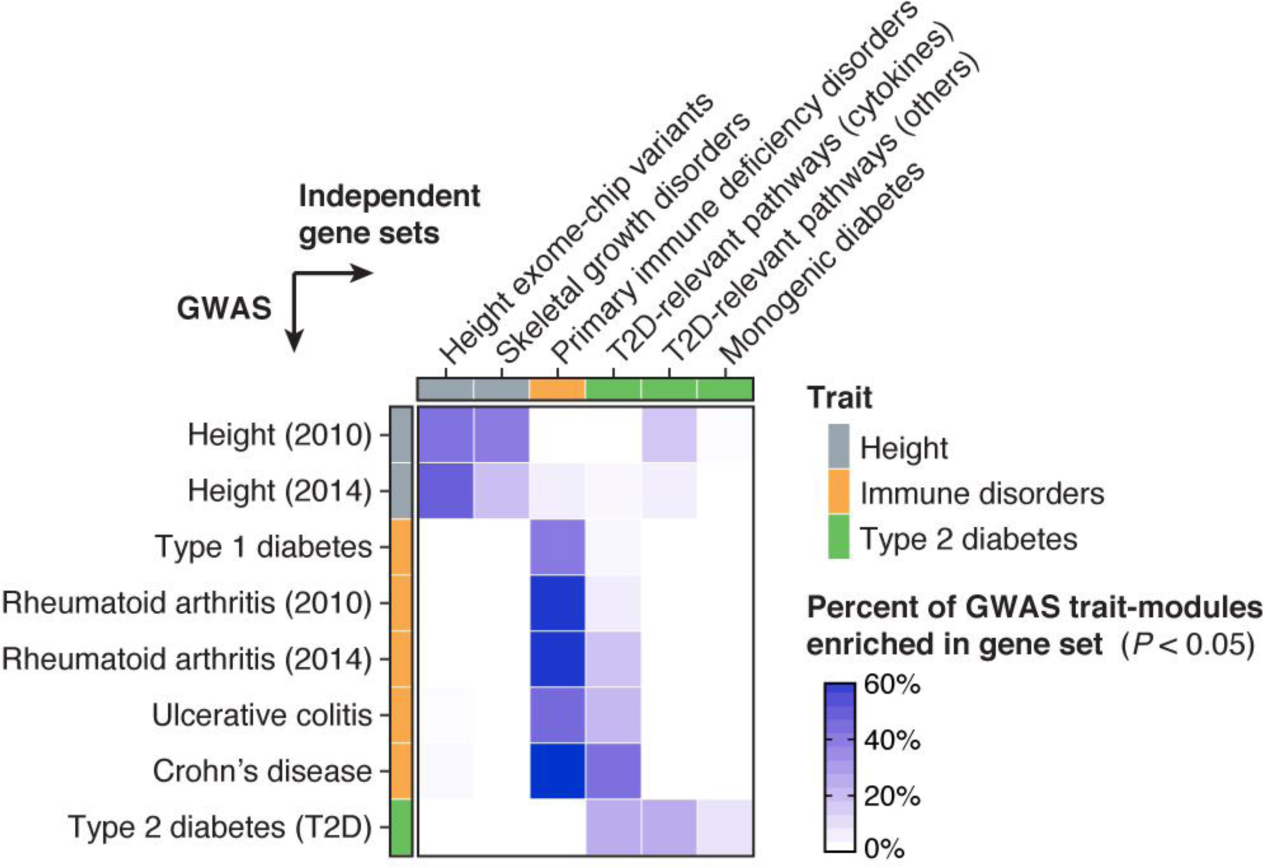

Supplement: Enrichment of trait-associated modules in curated gene sets from recent studies — . Enrichment of trait-associated modules in six curated gene sets from three recent studies. The first two gene sets were taken from Marouli et al.32 and correspond to genes comprising height-associated ExomeChip variants (n = 475 genes) and genes known to be involved in skeletal growth disorders (n = 266 genes), respectively. The third gene set was taken from de Lange et al.61 and corresponds to genes causing monogenic immunodeficiency disorders (n = 316 genes). Lastly, three gene sets relevant for type 2 diabetes (T2D) were taken from Fuchsberger et al.62 and correspond to genes in literature-curated pathways that are believed to be linked to T2D (we distinguished between genes in cytokine signalling pathways [n = 384 genes] and other pathways [n = 390 genes]) and genes causing monogenic diabetes (n = 81 genes). We then considered corresponding GWAS traits in our hold-out set, namely height, all immune-related disorders, and T2D. We tested all modules associated with these GWAS traits for enrichment in these six external gene sets. Enrichment was tested using the hypergeometric distribution and p-values were adjusted to control FDR using the Benjamini-Hochberg method. The heatmap shows for each GWAS (row) the fraction of trait-associated modules that significantly overlap with a given gene set (column). It can be seen that modules associated with a given trait predominantly overlap the external gene sets that are expected to be relevant for that trait. 61. de Lange, K. M. et al. Genome-wide association study implicates immune activation of multiple integrin genes in inflammatory bowel disease. Nat. Genet. 49, 256–261 (2017). 62. Fuchsberger, C. et al. The genetic architecture of type 2 diabetes. Nature 536, 41–47 (2016). [file 41592_2019_509_Fig16_ESM.jpg]

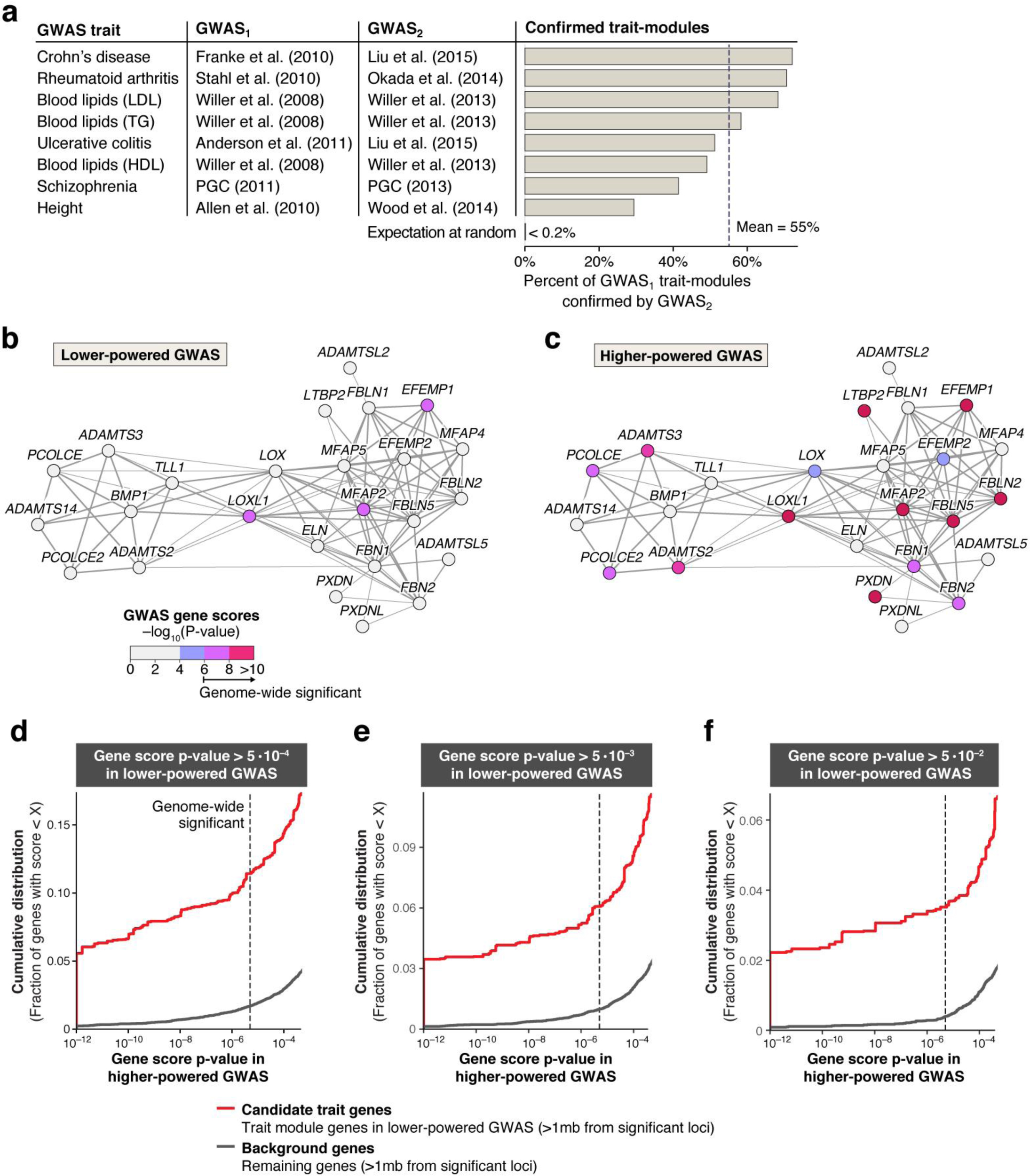

Supplement: Support of trait-module genes in higher-powered GWASs. — Trait-associated modules comprise many genes that show only borderline or no signal in the corresponding GWAS (called “candidate trait genes”). To assess whether modules correctly prioritized candidate trait genes, we considered eight traits for which older (lower-powered) and more recent (higher-powered) GWAS datasets were available in our holdout set. This allowed us to evaluate how well trait-associated modules and candidate trait genes predicted using the lower-powered GWAS datasets were supported in the higher-powered GWAS datasets. (a) Pairs of older (lower-powered) and more recent (higher-powered) GWASs used for the evaluation of module-based gene prioritization. The first column gives the trait and the second and third columns the corresponding GWASs. The bar plot shows the percentage of trait-associated modules from the first GWAS that are also trait-associated modules in the second GWAS. At the bottom, the expected percentage of confirmed modules at random is shown (i.e., assuming the trait-associated modules in the second GWAS were randomly selected from the set of predicted modules). (b) Height-associated module from Fig. 5 as an illustrative example (n = 25 genes). The module shows modest association to height in the lower-powered GWAS. Color indicates GWAS gene scores (FDR-corrected Pascal p-value = 0.04, see Methods). The signal is driven by three genes from different loci with significant scores (pink), while the remaining genes (grey) are predicted to be involved in height because of their module membership. (c) The module from (b) is supported in the higher-powered GWAS (q-value = 0.005). 45% of candidate trait genes (grey in (b)) are confirmed (pink). (d) Since high-powered GWASs typically result in many trait-associated genes, even random modules would have some genes “confirmed”. It is thus important to evaluate whether more candidate trait genes are confirmed than expected. Here we show support of candidate trait genes across the eight traits l [file 41592_2019_509_Fig17_ESM.jpg]

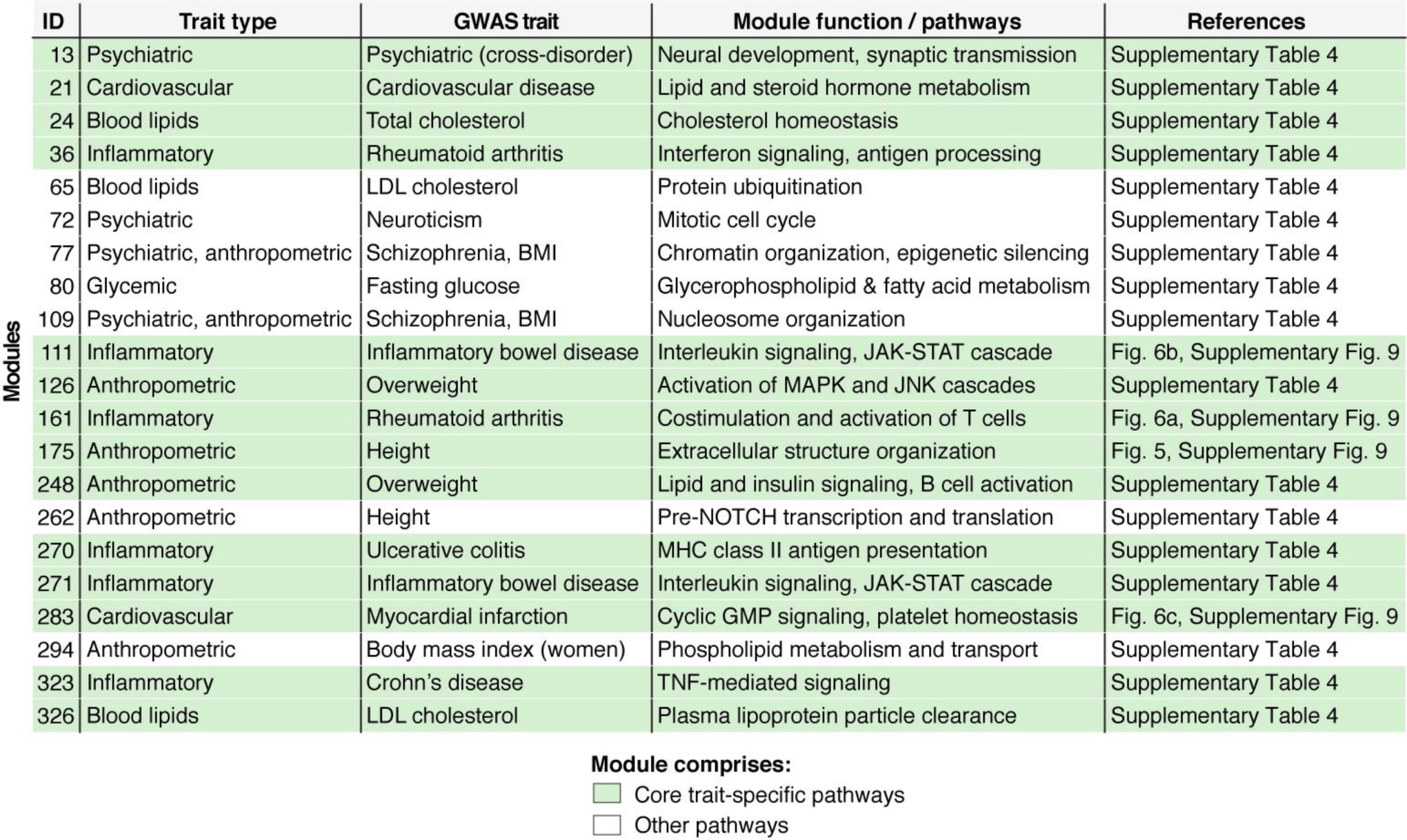

Supplement: Overview of Consensus Trait-modules in the STRING Network. — Overview of all 21 trait-associated consensus modules in the STRING protein-protein interaction network. The first three columns give the module ID, the trait type, and the specific GWAS trait that the module is associated to. We tested all modules for enrichment in GO annotation, mouse mutant phenotypes, and other pathway databases using the non-central hypergeometric test (Methods). The putative function of each module based on this enrichment analysis is summarized in the fourth column (see Figs. 5 and 6, Supplementary Fig. 9, and Supplementary Table 4 for details). Two thirds of the modules have functions that correspond to core pathways underlying the respective traits, while the remaining modules correspond either to generic pathways that play a role in diverse traits or to pathways without an established connection to the considered trait or disease. Only pathways with a well-established link to the trait were considered core pathways. Generic pathways, such as cell-cycle-related or epigenetic pathways, were not considered core pathways because they are relevant for many traits and tissues, making them more difficult to target therapeutically. For example, modules 77 and 109 are both associated with schizophrenia and comprise pathways related to epigenetic gene silencing and nucleosome organization, respectively. Although there is evidence that epigenetic mechanisms may play a role in schizophrenia, we considered this to be a generic pathway. [file 41592_2019_509_Fig18_ESM.jpg]

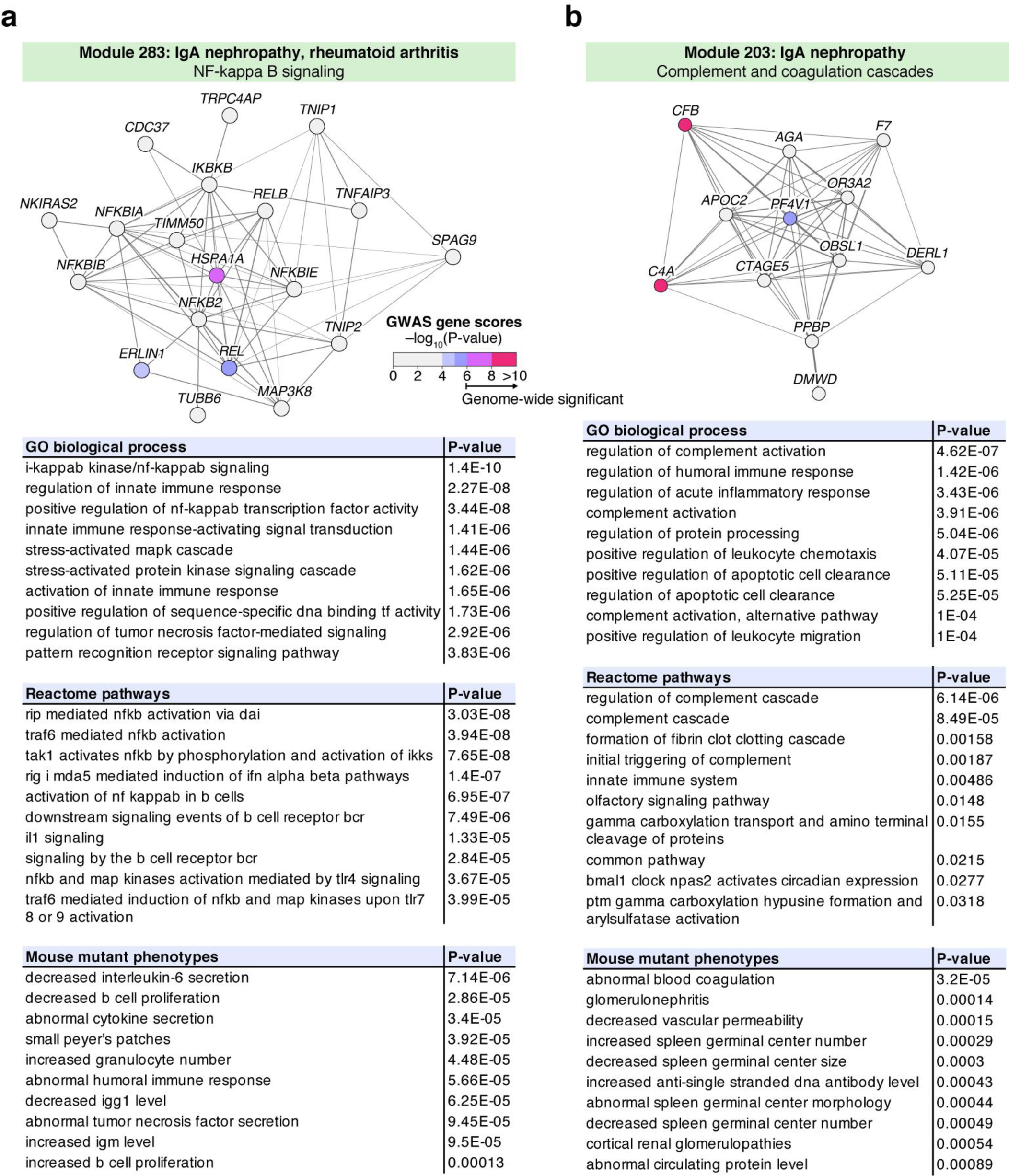

Supplement: Modules Associated with IgA Nephropathy. — The top ten enriched GO biological processes, Reactome pathways and mouse mutant phenotypes are shown for two IgA nephropathy (IgAN) associated modules. P-values were computed using the non-central hypergeometric distribution (Methods). (a) IgAN-associated module identified using the consensus analysis in the InWeb protein-protein interaction network (n = 19 genes). The module comprises immune-related NF-κB signaling pathways. Enriched mouse mutant phenotypes for module gene homologs include perturbed immunoglobulin levels (IgM and IgG1). The module implicates in particular the NF-κB subunit REL as a candidate gene. The REL locus does not reach genome-wide significance in current GWASs for IgAN but is known to be associated with other immune disorders such as rheumatoid arthritis. (b) IgAN-associated module identified by the best-performing method (K1) in the InWeb protein-protein interaction network (n = 12 genes). Besides finding complement factors that are known to play a role in the disease (CFB and C4A), the module implicates novel candidate genes such as the chemokine Platelet Factor 4 Variant 1 (PF4V1) from a sub-threshold locus, and is enriched for coagulation cascade, a process known to be involved in kidney disease62. The top two enriched mouse mutant phenotypes are precisely “abnormal blood coagulation” and “glomerulonephritis”. 62. Madhusudhan, T., Kerlin, B. A. & Isermann, B. The emerging role of coagulation proteases in kidney disease. Nat. Rev. Nephrol. 12, 94–109 (2016). [file 41592_2019_509_Fig19_ESM.jpg]
