# Supplementary material for: Assessment of network module identification across complex diseases
Source: Nat Methods. 2019 Aug 30;16(9):843–52. doi: 10.1038/s41592-019-0509-5 (PMC6719725; doi:10.1038/s41592-019-0509-5)
Supplement: Supplementary file 14 — Supplementary Figs. 1–13, and Supplementary Table 2. [file 41592_2019_509_MOESM1_ESM.pdf]

nature methods

SUPPLEMENTARY INFORMATION

https://doi.org/10.1038/s41592-019-0509-5

In the format provided by the authors and unedited.

Assessment of network module identification across complex diseases

Sarvenaz Choobdar[1,2,20], Mehmet E. Ahsen[3,117], Jake Crawford[4,117], Mattia Tomasoni[1,2], Tao Fang[5], David Lamparter[1,2,6], Junyuan Lin[7], Benjamin Hescott[8], Xiaozhe Hu[7], Johnathan Mercer[9,10], Ted Natoli[11], Rajiv Narayan[11], The DREAM Module Identification Challenge Consortium[12], Aravind Subramanian[11], Jitao D. Zhang[5], Gustavo Stolovitzky[3,13], Zoltán Kutalik[2,14], Kasper Lage[9,10,15], Donna K. Slonim[4,16], Julio Saez-Rodriguez[17,18], Lenore J. Cowen[4,7], Sven Bergmann[1,2,19,21]* and Daniel Marbach[1,2,5,21]*

[1]Department of Computational Biology, University of Lausanne, Lausanne, Switzerland. [2]Swiss Institute of Bioinformatics, Lausanne, Switzerland. [3]Icahn Institute for Genomics and Multiscale Biology and Department of Genetics and Genomic Sciences, Icahn School of Medicine at Mount Sinai, New York, NY, USA. [4]Department of Computer Science, Tufts University, Medford, MA, USA. [5]Roche Pharma Research and Early Development, Pharmaceutical Sciences, Roche Innovation Center Basel, F. Hoffmann-La Roche Ltd, Basel, Switzerland. [6]Verge Genomics, San Francisco, CA, USA. [7]Department of Mathematics, Tufts University, Medford, MA, USA. [8]College of Computer and Information Science, Northeastern University, Boston, MA, USA. [9]Department of Surgery, Massachusetts General Hospital, Harvard Medical School, Boston, MA, USA. [10]Stanley Center at the Broad Institute of MIT and Harvard, Cambridge, MA, USA. [11]Broad Institute of MIT and Harvard, Cambridge, MA, USA. [12]Full list of members appears at the end of the paper. [13]IBM T.J. Watson Research Center, Yorktown Heights, NY, USA. [14]University Institute of Primary Care and Public Health, University of Lausanne, Lausanne, Switzerland. [15]Institute for Biological Psychiatry, Mental Health Center Sct. Hans, University of Copenhagen, Roskilde, Denmark. [16]Department of Immunology, Tufts University School of Medicine, Boston, MA, USA. [17]Institute for Computational Biomedicine, Faculty of Medicine, Heidelberg University, Bioquant, Heidelberg, Germany. [18]RWTH Aachen University, Faculty of Medicine, Joint Research Center for Computational Biomedicine, Aachen, Germany. [19]Department of Integrative Biomedical Sciences, University of Cape Town, Cape Town, South Africa. [20]These authors contributed equally: Sarvenaz Choobdar, Mehmet E. Ahsen, Jake Crawford. [21]These authors jointly supervised this work: Sven Bergmann, Daniel Marbach. *e-mail: sven.bergmann@unil.ch; daniel.marbach.dm1@roche.com

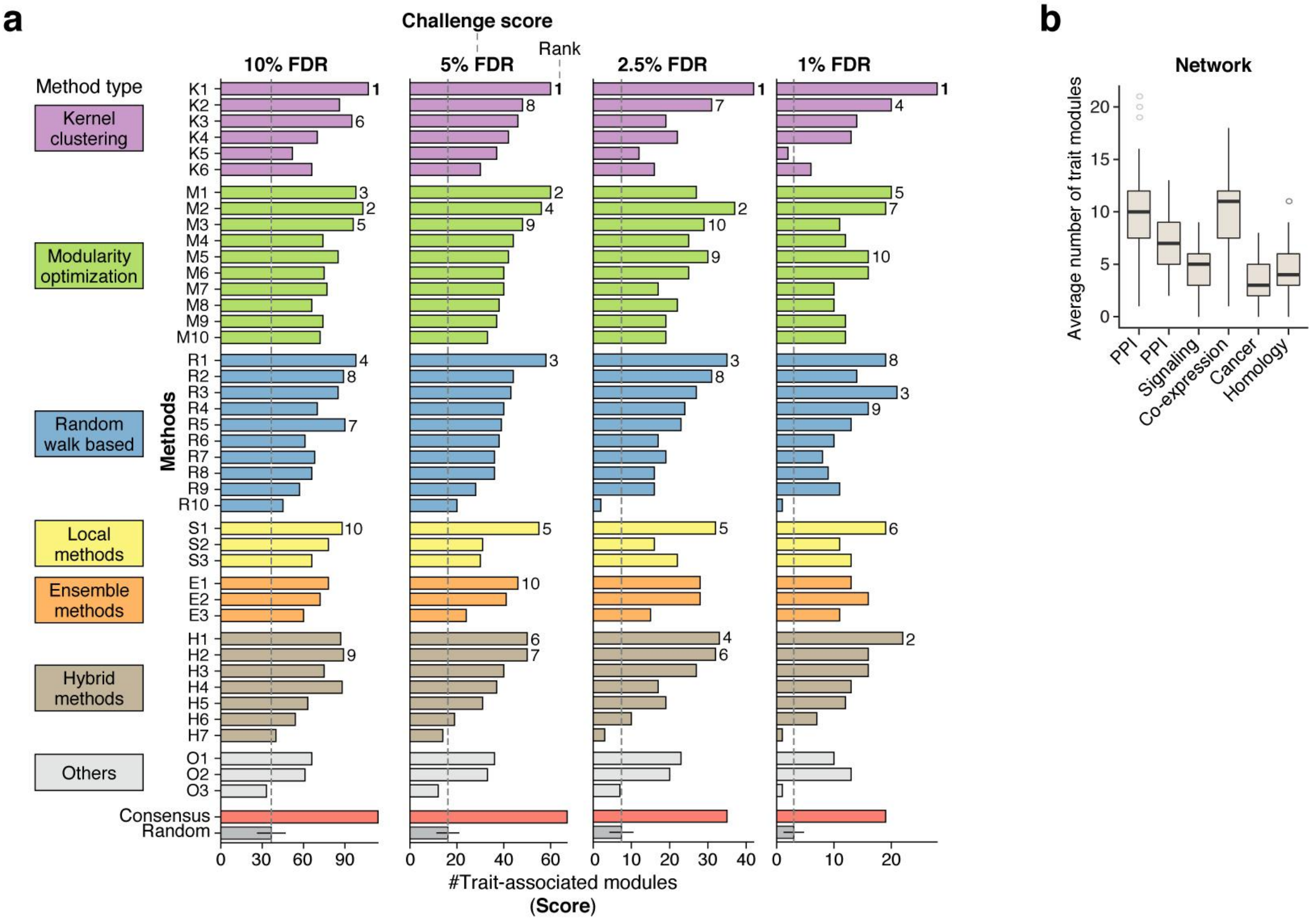

Supplementary Figure 1

Scores in Sub-challenge 1.

(a) Overall scores of the 42 module identification methods applied in Sub-challenge 1 at four different FDR cutoffs (10%, 5%, 2.5%, and 1% FDR). For explanation see legend of **Fig. 2b**, which shows the scores at 5% FDR (the predefined cutoff used for the challenge ranking). The top-performing method (K1) ranks first at all four cutoffs. The consensus prediction achieves the top score at 10% and 5% FDR, but not at the more stringent cutoffs.

(b) Average number of trait-associated modules across the 42 methods for each of the six networks. The most trait modules are found in the two protein-protein interaction (PPI) and the co-expression networks. Related to **Fig. 2d**, which shows the average number of trait modules relative to network size.

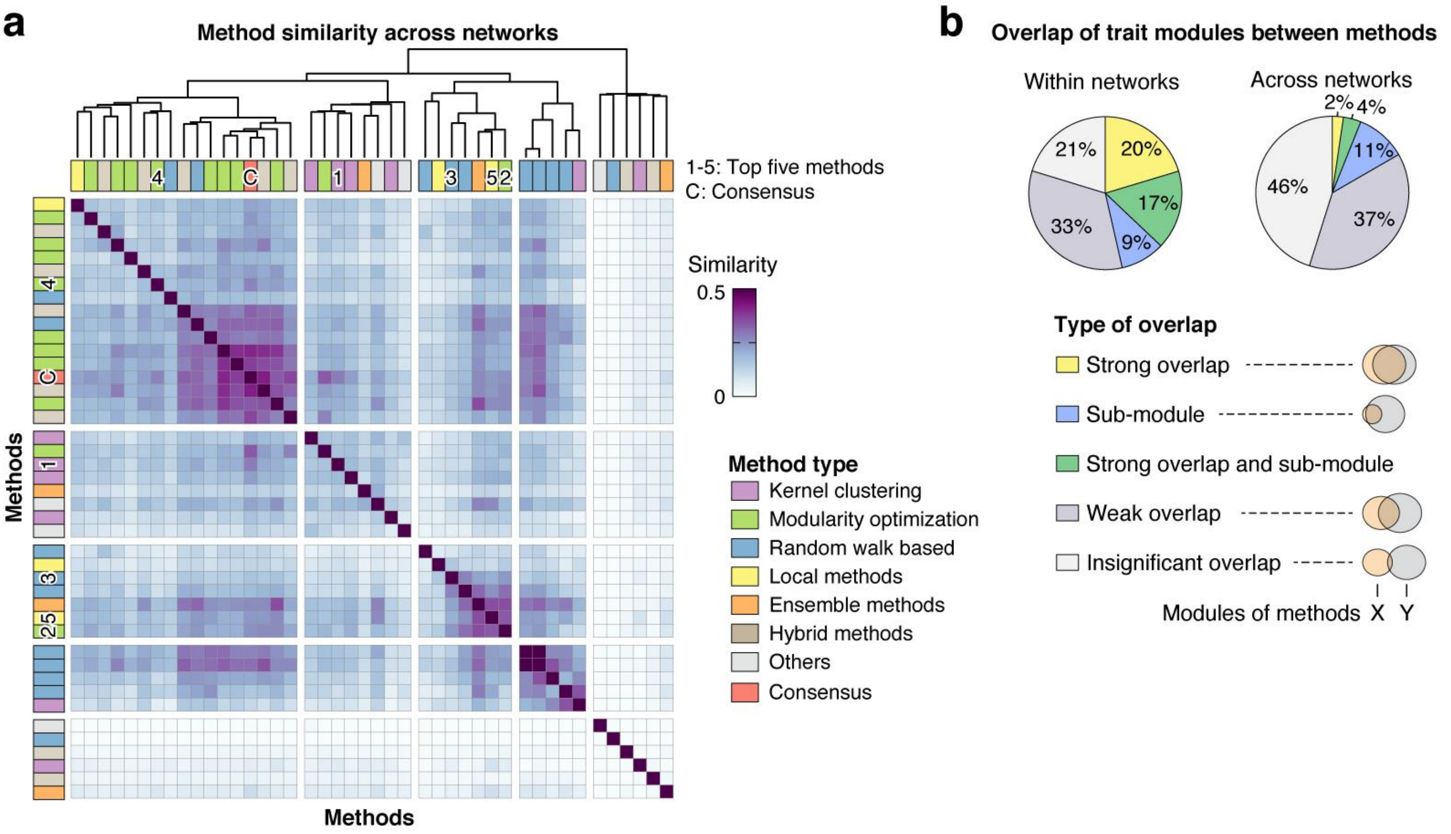

Supplementary Figure 2

Pairwise similarity of module predictions from different methods.

(a) Pairwise similarity of module predictions from different methods in Sub-challenge 1, averaged over all networks. Similarity was computed based on whether the same genes were clustered together by the two methods. Specifically, a prediction vector $\mathbf{P}_{mk}$ was defined for every method m and network k, specifying for every pair of genes whether they were co-clustered in the same module (Methods). The prediction vectors $\mathbf{P}_{mk}$ of method m for the six networks ($k = 1,2, \ldots ,6$) were then concatenated, forming a single vector $\mathbf{P}_m$ representing the module predictions of that method for all six networks. A corresponding distance matrix between the 42 methods was computed as described in Methods (Equation 1) and hierarchically clustered using Ward's method. The annotation row and column show the method type. The top five methods (1-5) and the consensus (C) are highlighted. The top methods did not converge to similar module predictions (they are not all grouped together in the hierarchical clustering). Related to **Fig. 3**, which shows similarity of module predictions from individual networks.

(b) Comparison of trait-associated modules identified by all challenge methods. Pie-charts show the percentage of trait modules that show overlap with at least one trait module from a different method in the same network (top) and in different networks (bottom). We distinguish between strong overlap, sub-modules, weak but statistically significant overlap, and insignificant overlap (Methods).

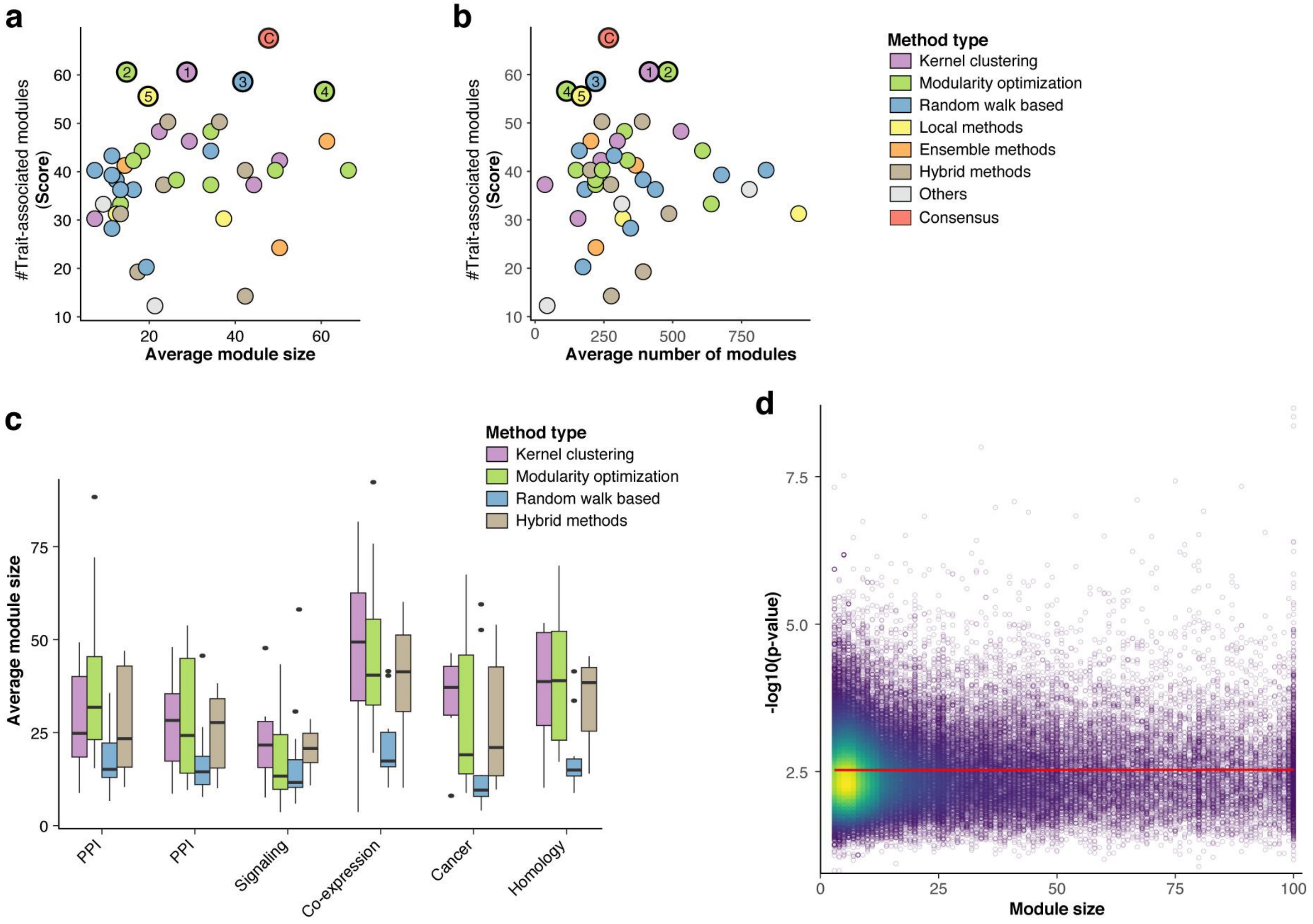

Supplementary Figure 3

Optimal module granularity is method- and network-specific.

All panels show results for single-network module identification methods (Sub-challenge 1).

(a) Average module size versus score for each of the 42 methods. The x-axis shows the average module size of a given method across the six networks. The y-axis shows the overall score of the method. Top teams (highlighted) produced modules of varying size, i.e., they did not converge to a similar module size during the leaderboard round. There is no significant correlation between module size and score (p-value = 0.13 using two-sided Pearson's correlation test), i.e., the scoring metric did not generally favor either small or large modules. Rather, when optimizing parameters during the leaderboard round, teams converged to very different granularities that led to the best performance for their specific methods.

(b) Average number of modules versus score for each method. The x-axis shows the average number of submitted modules across networks for a given method, and the y-axis shows the corresponding score. The top five teams (highlighted) submitted a variable number of modules (between 103 and 470 modules, on average, per network). There is no significant correlation between the number of submitted modules and the obtained score (p-value = 0.99 using two-sided Pearson's correlation test), i.e., the scoring metric was not biased to generally favor either a small or high number of submitted modules.

(c) Comparison of module sizes between networks and method types. For each network, boxplots show the distribution of average module sizes for kernel clustering (n = 6 methods), modularity optimization (n = 10 methods), random-walk-based (n = 10 methods), and hybrid methods (n = 7 methods; the remaining categories are not shown because they comprise only three methods each). Note that teams tuned the resolution (average module size) of their method during the leaderboard round. The variation in module size between different method categories and networks suggests that the optimal resolution is method- and network-specific. For example, teams using random-walk-based methods tended to choose a higher resolution (smaller average module size) than teams using kernel

clustering or modularity optimization methods. On average, modules were smallest in the signaling network and largest in the co-expression network.

(d) Module size versus trait-association p-value for individual modules from all methods and networks. For all n = 84,798 modules, the module size (x-axis) is plotted against the -log10 of the minimum Pascal p-value across all GWASs (y-axis). Color shows the density of points. By design, Pascal p-values are not confounded by module size[23], which is confirmed here (the regression line, shown in red, is flat; see also **Supplementary Fig. 4**).

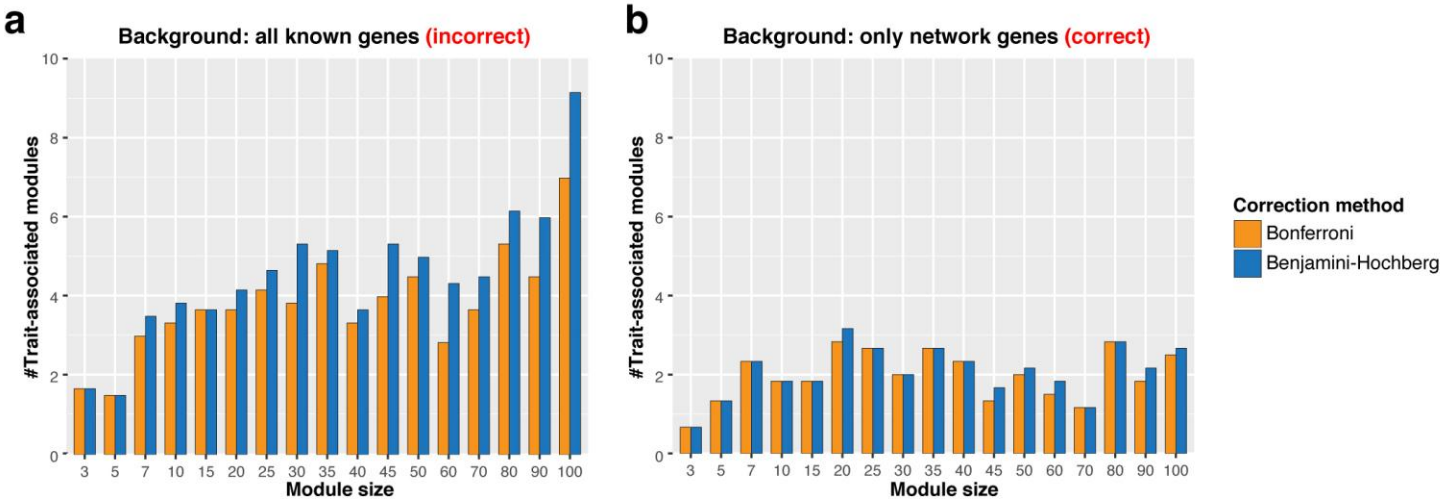

Supplementary Figure 4

Module granularity of random predictions does not correlate with score.

The panels show the average number of trait-associated modules for 17 random modularizations of the networks (i.e., networks were decomposed into random modules of the given sizes). Results are shown both for Bonferroni (orange) and Benjamini-Hochberg (blue) corrected p-values at a significance level of 0.05. The difference between the two panels is the background gene set used for the Pascal module enrichment test (see Methods).

(a) The complete set of all annotated genes is used as background to compute module enrichment (the UCSC known genes). This is an incorrect choice for the background because module genes are drawn from the network genes, which is a subset of all known genes. As expected, this incorrect choice of a background set leads to a higher number of trait-associated random modules than in Panel **b**, in particular for large modules.

(b) The set of all genes in a given network is used as background to compute module enrichment. This is the approach that was employed for the challenge scoring. Besides from very small modules of size 3, the module size does not affect the number of trait-associated random modules, i.e., our scoring methodology is not biased towards a specific module size (see also **Supplementary Fig. 3d**).

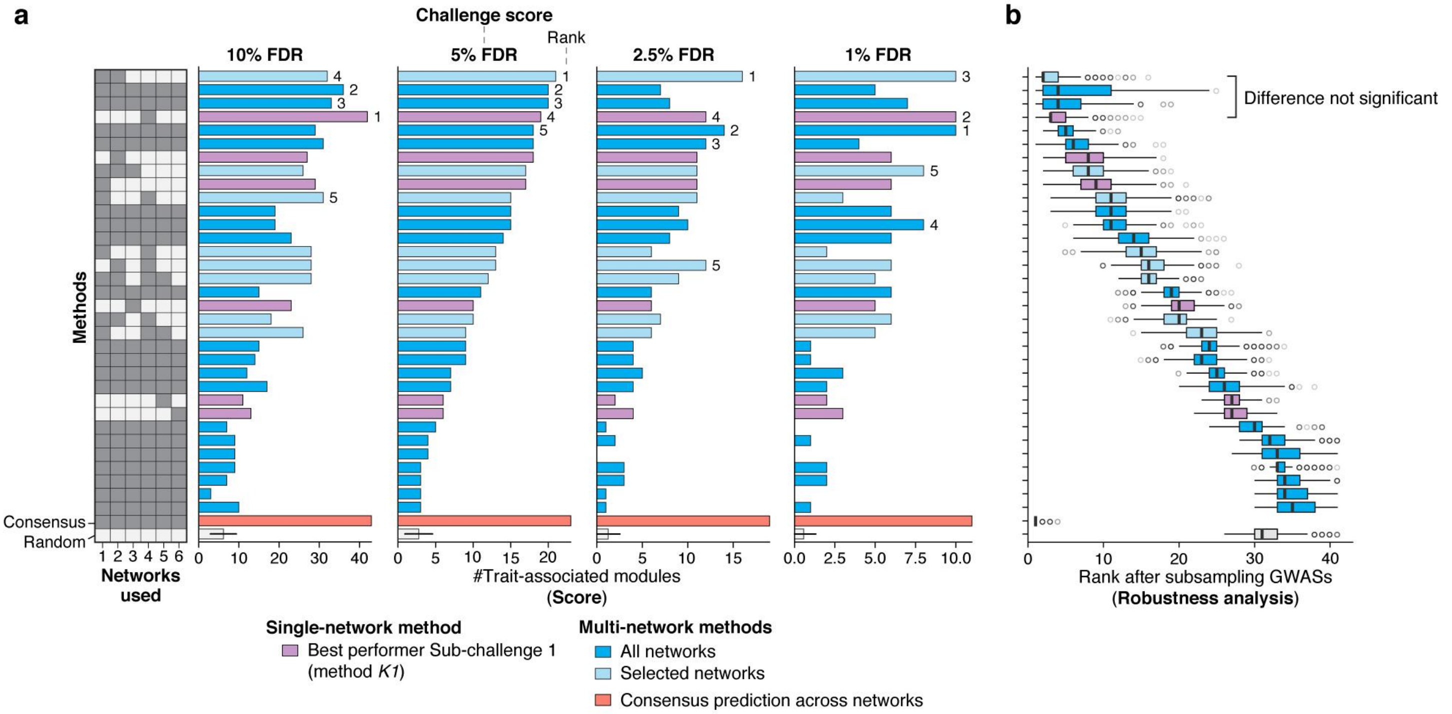

Supplementary Figure 5

Scores in Sub-challenge 2.

(a) Final scores of multi-network module identification methods in Sub-challenge 2 at four different FDR cutoffs (10%, 5%, 2.5%, and 1% FDR). For explanation see legend of **Fig. 3e**, which shows the scores at 5% FDR (the predefined cutoff used for the challenge ranking). Ranks are indicated for the top five teams (ties are broken according to robustness analysis described in **Panel b**). The multi-network consensus prediction (red) achieves the top score at each FDR cutoff. Interestingly, the performance of methods integrating all five networks (dark blue) seems to drop substantially at the more stringent FDR thresholds. For example, the second and third ranking methods at both 5% and 10% FDR, which integrated all five networks, performed poorly at the 2.5% and 1% FDR thresholds (see second and third row from the top). This suggests that not only the absolute number of trait-associated modules, but also their quality in terms of association strength could not be improved by considering multiple networks. As mentioned in the Discussion, the challenge networks may not have been sufficiently related for multi-network methods to reveal meaningful modules spanning several networks. Indeed, the similarity between our networks in terms of edge overlap was small (**Supplementary Fig. 6**). Of note, there is an important conceptual difference between the multi-network methods that teams applied (blue) and the multi-network consensus prediction (red). While the former performed modularization on blended or multi-layer networks, the latter integrated the single-network module predictions obtained from each individual network (see **Supplementary Fig. 7**). Results thus suggest that our multi-network consensus approach is better suited than multi-layer module identification methods when network similarity is low. Exploring the performance of these different approaches when applied to networks of varying similarity is a promising avenue for future work.

(b) Robustness of the overall ranking in Sub-challenge 2 was evaluated by subsampling the GWAS set used for evaluation 1,000 times. For each method, the resulting distribution of ranks is shown as a boxplot (using the 5% FDR cutoff for scoring). Related to **Fig. 2c**, which shows the same analysis for Sub-challenge 1. The difference between the top single-network module prediction and the top multi-network module predictions is not significant when sub-sampling the GWASs (Bayes factor < 3, see Methods section "Robustness analysis of challenge ranking").

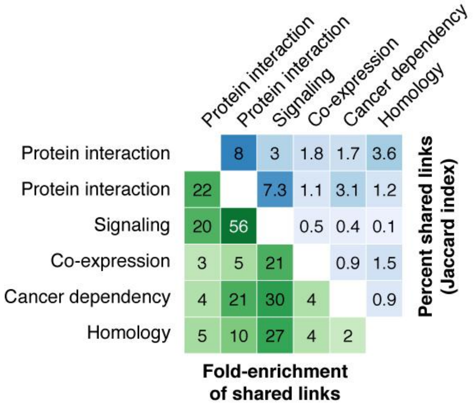

Supplementary Figure 6

Pairwise similarity of challenge networks.

Pairwise similarity of challenge networks. The upper triangle of the matrix shows the percent of shared links (the Jaccard index multiplied by 100) and the lower triangle shows the fold-enrichment of shared links compared to the expected number of shared links at random. The two protein-protein interaction networks are the two most similar networks, yet they have only 8% shared edges. Of note, a recent study has found similarly low overlap between protein-protein interaction networks from different sources, suggesting that these molecular maps are still far from complete[60].

60. Huang, J. K. *et al.* Systematic Evaluation of Molecular Networks for Discovery of Disease Genes. *Cell Syst.* **6**, 484-495.e5 (2018)

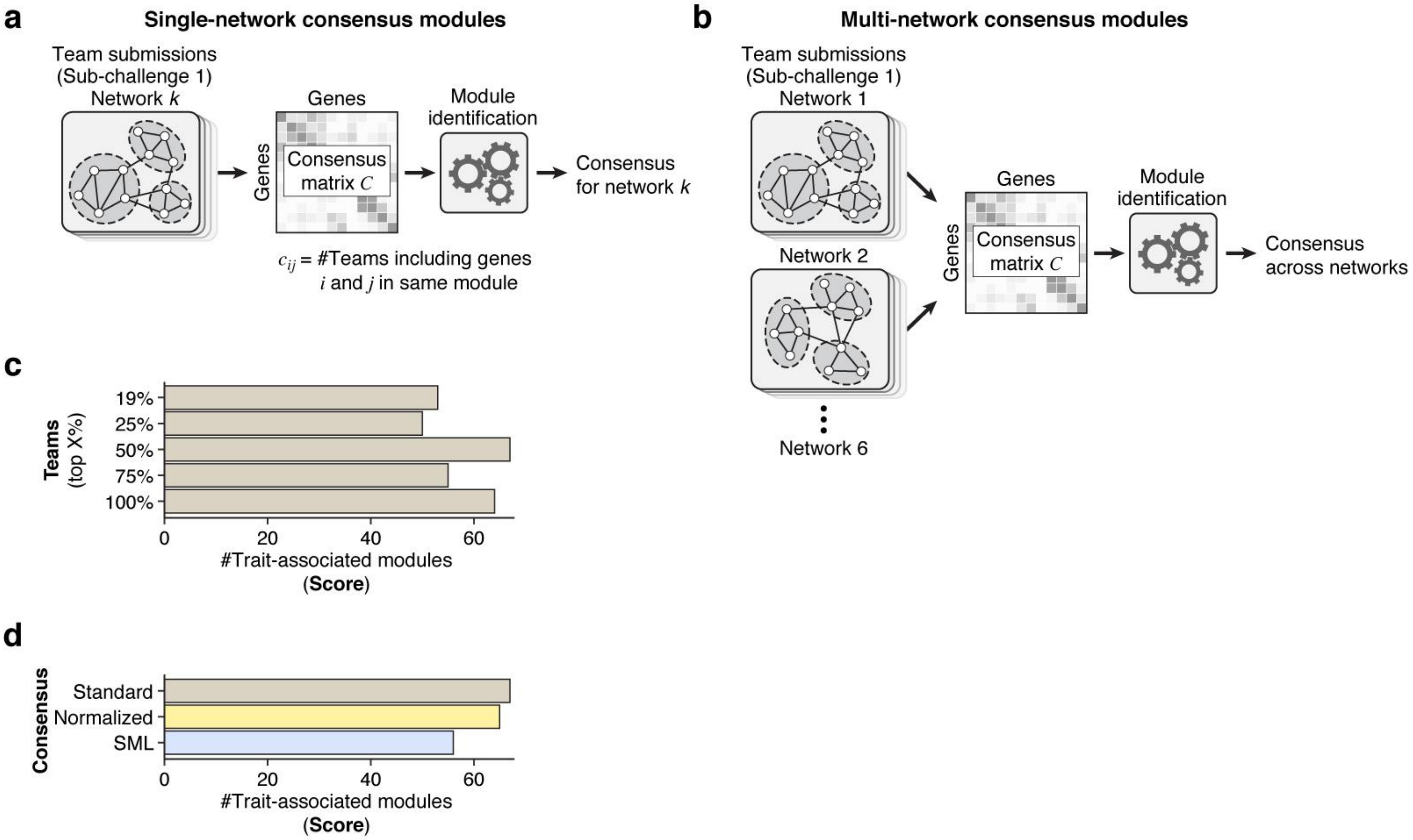

Supplementary Figure 7

Consensus Module Predictions.

(a) Schematic of the approach used to generate single-network consensus module predictions for Sub-challenge 1. For each network, module predictions from the top 50% of teams were integrated in a consensus matrix *C*, where each element c_{ij} gives the fraction of teams that clustered gene *i* and *j* together in the same module in the given network (performance as the percentage of considered teams is varied is shown in (**c**)). The overall score from the leaderboard round was used to select the top 50% of teams, i.e., the same set of teams was used for each network. The consensus matrix of each network was then clustered using the top-performing module identification method of the challenge (method K1; see Methods).

(b) The approach used to generate multi-network consensus module predictions for Sub-challenge 2 was exactly the same as for single-network predictions, except that team submissions from all networks were integrated in the consensus matrix *C*. In other words, as input we still used the single-network predictions of the top 50% of teams from Sub-challenge 1, but instead of forming a consensus matrix for each network, a single cross-network consensus matrix was formed. This cross-network consensus matrix is then clustered using method K1 as described above (see Methods).

(c) Scores of the single-network consensus predictions as the percentage of integrated teams is varied. We considered the top 25%, 50%, 75% and 100% of teams, as well as the top eight (19%) teams (these are the teams that ranked 2nd, or tied with the team that ranked 2nd, at any of the considered FDR cutoffs).

(d) Performance of different methods to construct the consensus matrix *C*. In addition to the basic approach described above (*Standard*), two more sophisticated approaches to construct the consensus matrix were evaluated (*Normalized* and *SML*). In each case, the same set of team submissions were integrated (top 50%) and method K1 was applied to cluster the resulting consensus matrix.

The first alternative (*Normalized*) is similar to the basic method but further assumes that appearing together in a smaller cluster is stronger evidence that a pair of genes is associated than appearing together in a larger cluster. Thus, each cluster's contribution to the consensus matrix was normalized by the size of the cluster. Furthermore, we normalized the *ij*-entry of the consensus matrix by the number of methods that assigned gene *i* to a cluster, thus taking the presence of background genes into account. We found that the consensus still achieved the top score with these normalizations, but there was no improvement compared to the basic approach.

The second method is a very different approach called Spectral Meta Learner (SML)[56]. SML is an unsupervised ensemble method designed for two-class classification problems. Briefly, it takes a matrix of predictions P, where each row corresponds to different samples being classified and the columns correspond to different methods. Accordingly, each matrix element P_{ij} is the class (0 or 1) assigned to sample i by method j. Under the assumption of conditional independence of methods given class labels, SML can estimate the balanced accuracy of each classifier in a totally unsupervised manner using only the prediction matrix P. The algorithm then uses this information to construct an ensemble classifier in which the contribution of each classifier is proportional to its estimated performance (balanced accuracy). The module identification problem is an unsupervised problem by its nature and we applied the SML algorithm as a new way for constructing consensus modules. For each method m and network k, we created a vector of prediction P_{mk}, of size $N_{G_k}$by N_{G_k}, where $N_{G_k}$is the number genes in network as follows:

$P_{mk}(i,j) = 1,\ \ if\ method\ m\ puts\ genes\ i\ and\ j\ in\ the\ same\ module$ (1)

$P_{mk}(i,j) = 0,\ \ otherwise.$

For each network, we constructed the prediction matrix P with each column P_m defined as above. We then provided this matrix as input to the SML algorithm. The SML algorithm outputs a consensus matrix, which assigns a weight between each pair of genes. We found that SML did not perform well in the context of this challenge, likely because the underlying assumption of SML is that top-performing methods converge to similar predictions, which was not the case here (see **Fig. 3** and **Supplementary Fig. 2**).

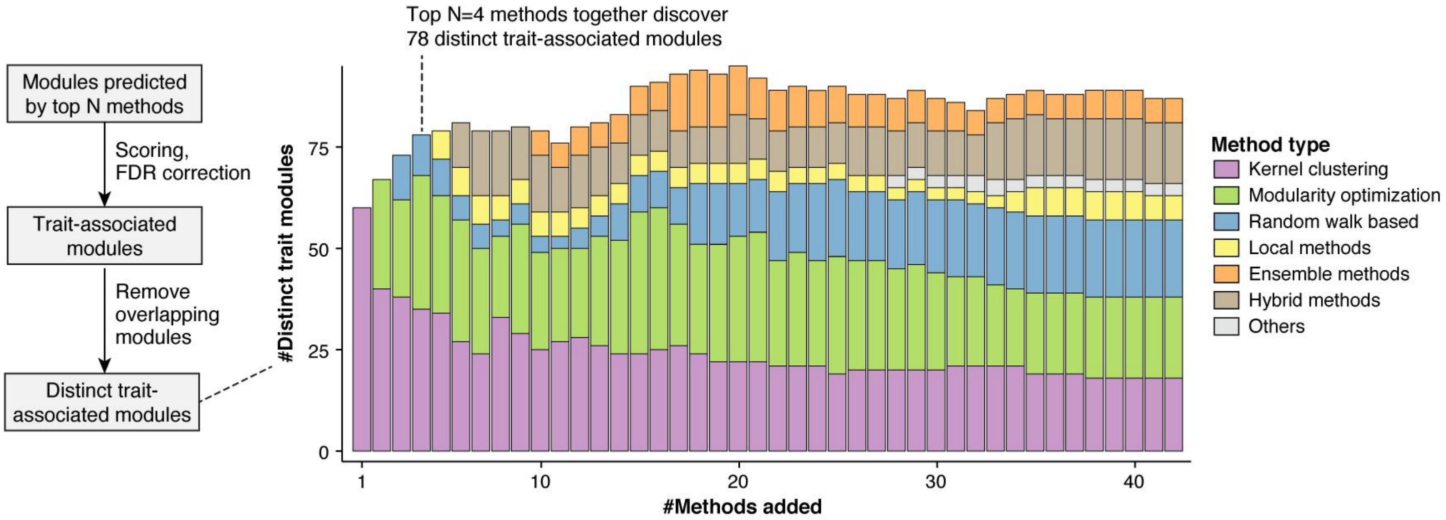

Supplementary Figure 8

Number of distinct trait-associated modules recovered by top methods.

Number of distinct trait-associated modules recovered by the top *K* methods. Here, we did not form consensus modules. Instead, given the top *K* methods, we considered the set including all individual modules predicted by these methods and scored them with the same pipeline as used for the challenge submissions. We then evaluated how many "distinct" trait-associated modules were recovered by these methods. Distinct modules were defined as modules that do not show any significant overlap among each other. Overlap between pairs of modules was evaluated using the hypergeometric distribution and called significant at 5% FDR (Benjamini-Hochberg adjusted p-value < 0.05). From the set of trait-associated modules discovered by the top *K* methods, we thus derived the subset of distinct trait-associated modules (when several modules overlapped significantly, only the module with the most significant GWAS p-value was retained). Although the resulting scores (number of distinct trait-associated modules) cannot be directly compared with the challenge scores (because module predictions had to be strictly non-overlapping in the challenge), it is instructive to see how many distinct trait modules can be recovered when applying multiple methods. The stacked bars (colors) further show how many of the distinct trait modules are contributed by each method category. The number of distinct trait modules is not monotonically increasing as more methods are added because the larger sets of modules also increase the multiple testing burden of the GWAS scoring. The top four methods together discover 78 distinct trait-associated modules. Relatively little is gained by adding a higher number of methods.

a

Module 175: Height
Extracellular structure organization

Visualized in **Fig. 5**

Mouse mutant phenotypes	P-value
abnormal cutaneous elastic fiber morphology	9.7E-04
emphysema	7.9E-03
loose skin	2.2E-02

Reactome pathways	P-value
collagen formation	9.7E-07
extracellular matrix organization	1.5E-05

GO biological process	P-value
extracellular matrix organization	<1E-12
extracellular structure organization	<1E-12
embryonic eye morphogenesis	6.1E-04
post-embryonic eye morphogenesis	6.2E-03
post-embryonic aaaaaorgan morphogenesis	1.4E-02
extracellular matrix disassembly	1.8E-02
collagen fibril organization	2.0E-02
supramolecular fiber organization	2.7E-02

c

Module 111: Inflammatory bowel disease
Interleukin signaling, JAK-STAT cascade

Visualized in **Fig. 6b**

Mouse mutant phenotypes	P-value
decreased interferon-gamma secretion	<1E-12
abnormal t-helper 1 physiology	5.7E-03
increased susceptibility to experimental autoimmune encephalomyelitis	1.2E-02

Reactome pathways	P-value
il 6 signaling	4.8E-05
growth hormone receptor signaling	1.8E-04
signaling by ils	2.8E-04
signaling by scf kit	1.5E-03
cytokine signaling in immune system	8.4E-03
il 7 signaling	1.1E-02
signaling by fgfr1 fusion mutants	3.7E-02

GO biological process	P-value
adaptive immune response	<1E-12
cytokine-mediated signaling pathway	1.2E-09
cellular response to cytokine stimulus	1.3E-07
response to cytokine	2.5E-07
regulation of lymphocyte activation	9.5E-07
regulation of cell activation	3.4E-06
jak-stat cascade	5.7E-06
positive regulation of cell activation	3.3E-04
jak-stat cascade involved in growth hormone signaling pathway	1.0E-02

b

Module 161: Rheumatoid arthritis
Costimulation and activation of T cells

Visualized in **Fig. 6a**

Mouse mutant phenotypes	P-value
decreased cd8-positive, alpha-beta t cell number	3.0E-05
increased interferon-gamma secretion	1.8E-03
decreased t cell proliferation	2.0E-03
increased double-negative t cell number	3.8E-03
abnormal t cell differentiation	4.1E-03
decreased double-positive t cell number	5.2E-03
abnormal t cell physiology	7.0E-03
abnormal regulatory t cell physiology	8.0E-03
abnormal double-negative t cell morphology	1.4E-02
abnormal cd8-positive, alpha beta t cell morphology	3.3E-02

Reactome pathways	P-value
generation of second messenger molecules	<1E-12
tcr signaling	<1E-12
pd1 signaling	<1E-12
costimulation by the cd28 family	<1E-12
phosphorylation of cd3 and tcr zeta chains	4.6E-07
translocation of zap 70 to immunological synapse	1.3E-05
immunoregulatory interactions	3.3E-05
downstream tcr signaling	4.9E-05

GO biological process	P-value
immune response-activating cell surface receptor signaling	<1E-12
immune response-activating signal transduction	<1E-12
immune response-regulating signaling pathway	<1E-12
immune response-regulating cell surface receptor signaling	<1E-12
regulation of cell-cell adhesion	<1E-12
positive regulation of cell-cell adhesion	<1E-12
regulation of cell adhesion	<1E-12
lymphocyte costimulation	<1E-12
t cell costimulation	<1E-12
t cell activation	<1E-12
positive regulation of cell adhesion	<1E-12
positive regulation of immune response	<1E-12
antigen receptor-mediated signaling pathway	<1E-12
t cell receptor signaling pathway	<1E-12
regulation of t cell activation	<1E-12
regulation of cell activation	<1E-12
positive regulation of cell activation	<1E-12
positive regulation of t cell activation	<1E-12
regulation of lymphocyte activation	<1E-12
adaptive immune response	4.8E-05
positive regulation of interleukin-2 biosynthetic process	1.3E-04
regulation of interleukin-2 biosynthetic process	1.0E-03
positive regulation of t cell proliferation	1.6E-03
regulation of t cell proliferation	2.3E-03
positive regulation of interleukin-2 production	6.8E-03
positive regulation of lymphocyte proliferation	7.2E-03
positive regulation of mononuclear cell proliferation	7.4E-03

d

Module 283: Myocardial infarction
Cyclic GMP signaling, platelet homeostasis

Visualized in **Fig. 6c**

Mouse mutant phenotypes	P-value
-	-

Reactome pathways	P-value
nitric oxide stimulates guanylate cyclase	2.4E-06
platelet homeostasis	1.3E-02

GO biological process	P-value
positive regulation of cgmp biosynthetic process	2.1E-03
nitric oxide mediated signal transduction	2.6E-03
positive regulation of cgmp metabolic process	3.5E-03
regulation of cgmp biosynthetic process	4.4E-03
regulation of cgmp metabolic process	1.6E-02

Supplementary Figure 9

Functional Enrichment for Example Modules.

Enrichment p-values for mouse mutant phenotypes, Reactome pathways and GO biological processes are shown for four example modules discussed in the main text. P-values were computed using the non-central hypergeometric distribution and adjusted using the Bonferroni method (Methods). Results for the remaining trait-associated modules from the consensus analysis in the STRING protein-protein interaction network are shown in **Supplementary Fig. 12** and **Supplementary Table 4**. Functional enrichment analysis for additional pathway databases and modules from all methods and networks are available on the challenge website.

(a) Module associated with height described in **Fig. 5** (n = 25 genes).

(b) Module associated with rheumatoid arthritis described in **Fig. 6a** (n = 25 genes).

(c) Module associated with inflammatory bowel disease described in **Fig. 6b** (n = 42 genes).

(d) Module associated with myocardial infarction described in **Fig. 6c** (n = 36 genes).

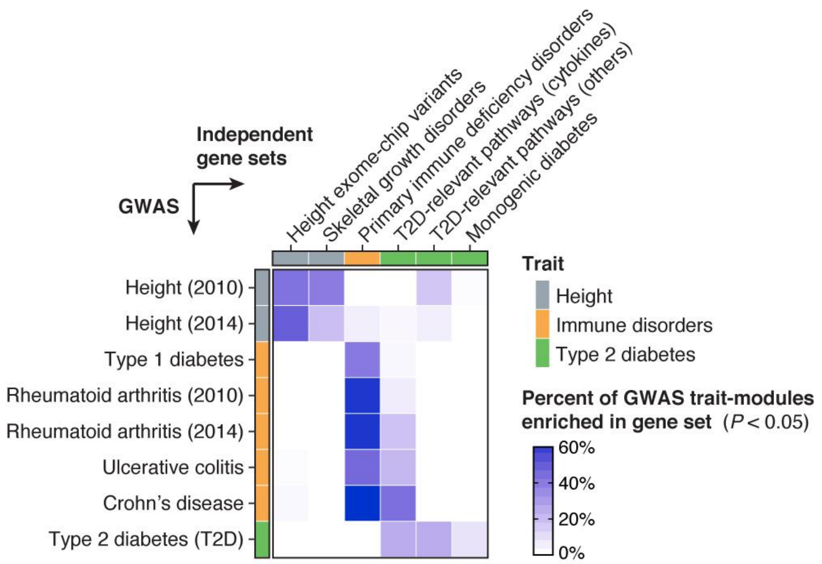

Supplementary Figure 10

Enrichment of trait-associated modules in curated gene sets from recent studies.

Enrichment of trait-associated modules in six curated gene sets from three recent studies. The first two gene sets were taken from Marouli et al.[32] and correspond to genes comprising height-associated ExomeChip variants (n = 475 genes) and genes known to be involved in skeletal growth disorders (n = 266 genes), respectively. The third gene set was taken from de Lange et al.[61] and corresponds to genes causing monogenic immunodeficiency disorders (n = 316 genes). Lastly, three gene sets relevant for type 2 diabetes (T2D) were taken from Fuchsberger et al.[62] and correspond to genes in literature-curated pathways that are believed to be linked to T2D (we distinguished between genes in cytokine signalling pathways [n = 384 genes] and other pathways [n = 390 genes]) and genes causing monogenic diabetes (n = 81 genes). We then considered corresponding GWAS traits in our hold-out set, namely height, all immune-related disorders, and T2D. We tested all modules associated with these GWAS traits for enrichment in these six external gene sets. Enrichment was tested using the hypergeometric distribution and p-values were adjusted to control FDR using the Benjamini-Hochberg method. The heatmap shows for each GWAS (row) the fraction of trait-associated modules that significantly overlap with a given gene set (column). It can be seen that modules associated with a given trait predominantly overlap the external gene sets that are expected to be relevant for that trait.

61. de Lange, K. M. *et al.* Genome-wide association study implicates immune activation of multiple integrin genes in inflammatory bowel disease. *Nat. Genet.* **49**, 256–261 (2017).

62. Fuchsberger, C. et al. The genetic architecture of type 2 diabetes. *Nature* **536**, 41–47 (2016).

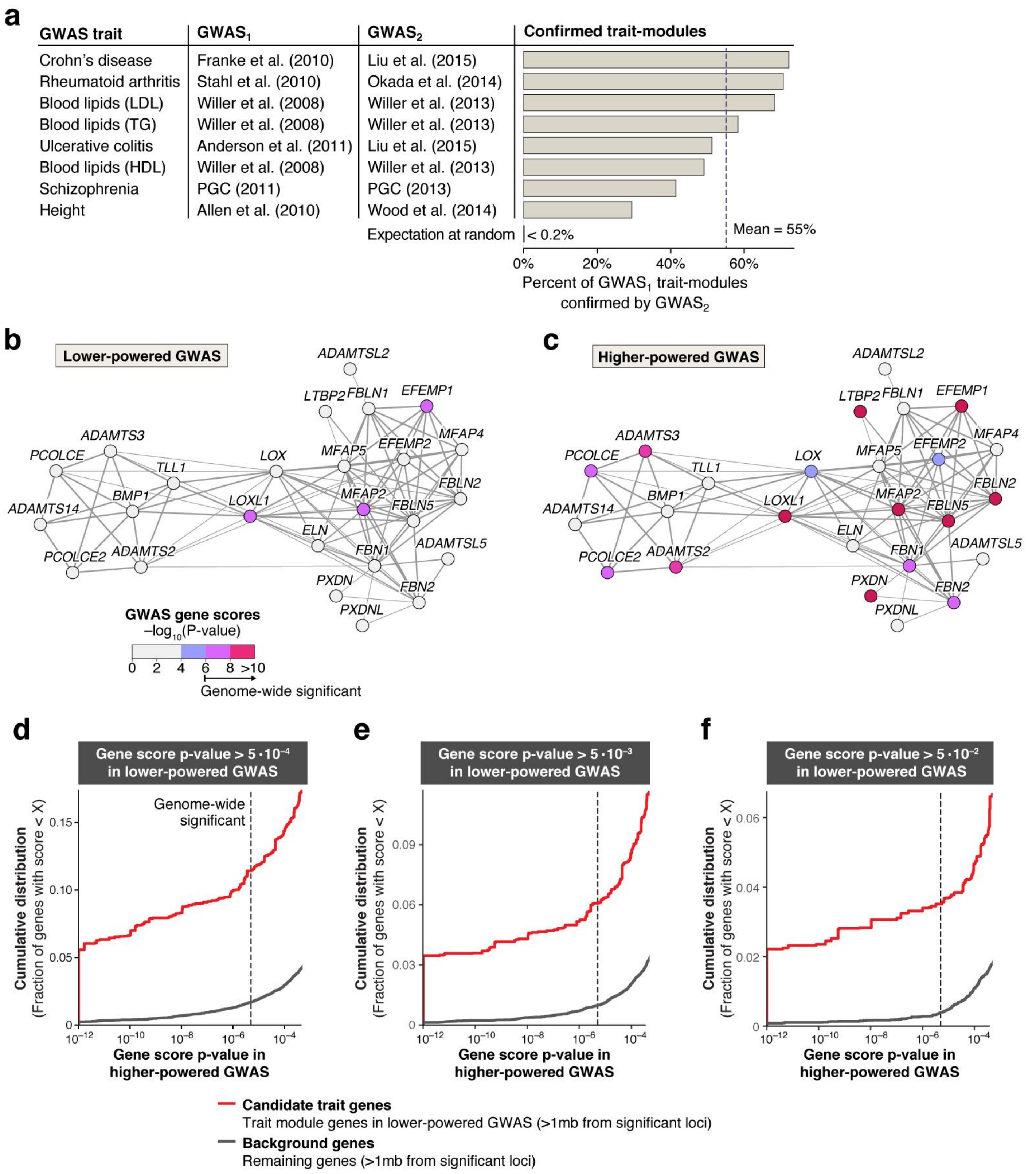

Supplementary Figure 11

Support of trait-module genes in higher-powered GWASs.

Trait-associated modules comprise many genes that show only borderline or no signal in the corresponding GWAS (called “candidate trait genes”). To assess whether modules correctly prioritized candidate trait genes, we considered eight traits for which older (lower-powered) and more recent (higher-powered) GWAS datasets were available in our holdout set. This allowed us to evaluate how well trait-associated modules and candidate trait genes predicted using the lower-powered GWAS datasets were supported in the higher-powered GWAS datasets.

(a) Pairs of older (lower-powered) and more recent (higher-powered) GWASs used for the evaluation of module-based gene prioritization. The first column gives the trait and the second and third columns the corresponding GWASs. The bar plot shows the percentage of trait-associated modules from the first GWAS that are also trait-associated modules in the second GWAS. At the bottom, the expected percentage of confirmed modules at random is shown (i.e., assuming the trait-associated modules in the second GWAS were randomly selected from the set of predicted modules).

(b) Height-associated module from **Fig. 5** as an illustrative example (n = 25 genes). The module shows modest association to height in the lower-powered GWAS. Color indicates GWAS gene scores (FDR-corrected Pascal p-value = 0.04, see Methods). The signal is driven by three genes from different loci with significant scores (pink), while the remaining genes (grey) are predicted to be involved in height because of their module membership.

(c) The module from (**b**) is supported in the higher-powered GWAS (*q*-value = 0.005). 45% of candidate trait genes (grey in (**b**)) are confirmed (pink).

(d) Since high-powered GWASs typically result in many trait-associated genes, even random modules would have some genes “confirmed”. It is thus important to evaluate whether more candidate trait genes are confirmed than expected. Here we show support of candidate trait genes across the eight traits listed in (**a**). The lower-powered GWASs were used to predict candidate trait genes, defined as genes that: (*i*) are within a trait-associated module in the lower-powered GWAS; (*ii*) have a high gene p-value (p > 5E-4, i.e., two orders of magnitude above the genome-wide significance threshold of 5E-6 (cf. grey genes in (**a**)) and (*iii*) are located more than one megabase away from the nearest significant locus of the corresponding GWAS. Gene p-values were computed using Pascal as described above. Finally, the Pascal p-value of all candidate trait genes was evaluated for the higher-powered GWAS (n = 2,254 genes considering trait-modules from all methods). Since there is a genome-wide tendency for p-values to become more significant in higher-powered GWAS data[38], Pascal p-values were also evaluated for a background gene set (all genes that meet the two conditions (*ii*, *iii*), but do not belong to trait-associated modules of the lower-powered GWAS). The plot shows the cumulative distribution of gene scores in the higher-powered GWASs for candidate trait genes (red line) and genes in the background set (grey line). a substantial fraction of module genes that do not show any signal and are located far from any significant locus in the lower-powered GWAS are subsequently confirmed by the higher-powered GWAS.

(e) Since candidate trait genes (i.e., genes satisfying the three conditions (*i*-*iii*) described above) could still have lower p-values than genes in the background set (i.e., genes satisfying the two conditions (*ii*, *iii*)), we repeated the same analysis with higher gene p-value thresholds for condition (*ii*): p-value > 5E-3 (n = 2,185 genes) (**e**) and p-value > 5E-2 (n = 1,969 genes) (**f**). For this range the “discovery” gene score p-values in the candidate set and the background set are much more similar. Although there may remain some confounding, the same trend as in (**d**) is observed, indicating that the result is robust. This suggests that modules are predictive for trait-associated genes and could potentially be used to prioritize candidate genes for follow-up studies, for instance.

ID	Trait type	GWAS trait	Module function / pathways	References
13	Psychiatric	Psychiatric (cross-disorder)	Neural development, synaptic transmission	Supplementary Table 4
21	Cardiovascular	Cardiovascular disease	Lipid and steroid hormone metabolism	Supplementary Table 4
24	Blood lipids	Total cholesterol	Cholesterol homeostasis	Supplementary Table 4
36	Inflammatory	Rheumatoid arthritis	Interferon signaling, antigen processing	Supplementary Table 4
65	Blood lipids	LDL cholesterol	Protein ubiquitination	Supplementary Table 4
72	Psychiatric	Neuroticism	Mitotic cell cycle	Supplementary Table 4
77	Psychiatric, anthropometric	Schizophrenia, BMI	Chromatin organization, epigenetic silencing	Supplementary Table 4
80	Glycemic	Fasting glucose	Glycerophospholipid & fatty acid metabolism	Supplementary Table 4
109	Psychiatric, anthropometric	Schizophrenia, BMI	Nucleosome organization	Supplementary Table 4
111	Inflammatory	Inflammatory bowel disease	Interleukin signaling, JAK-STAT cascade	Fig. 6b, Supplementary Fig. 9
126	Anthropometric	Overweight	Activation of MAPK and JNK cascades	Supplementary Table 4
161	Inflammatory	Rheumatoid arthritis	Costimulation and activation of T cells	Fig. 6a, Supplementary Fig. 9
175	Anthropometric	Height	Extracellular structure organization	Fig. 5, Supplementary Fig. 9
248	Anthropometric	Overweight	Lipid and insulin signaling, B cell activation	Supplementary Table 4
262	Anthropometric	Height	Pre-NOTCH transcription and translation	Supplementary Table 4
270	Inflammatory	Ulcerative colitis	MHC class II antigen presentation	Supplementary Table 4
271	Inflammatory	Inflammatory bowel disease	Interleukin signaling, JAK-STAT cascade	Supplementary Table 4
283	Cardiovascular	Myocardial infarction	Cyclic GMP signaling, platelet homeostasis	Fig. 6c, Supplementary Fig. 9
294	Anthropometric	Body mass index (women)	Phospholipid metabolism and transport	Supplementary Table 4
323	Inflammatory	Crohn’s disease	TNF-mediated signaling	Supplementary Table 4
326	Blood lipids	LDL cholesterol	Plasma lipoprotein particle clearance	Supplementary Table 4

Modules

Module comprises:
Core trait-specific pathways
Other pathways

Supplementary Figure 12

Overview of Consensus Trait-modules in the STRING Network.

Overview of all 21 trait-associated consensus modules in the STRING protein-protein interaction network. The first three columns give the module ID, the trait type, and the specific GWAS trait that the module is associated to. We tested all modules for enrichment in GO annotation, mouse mutant phenotypes, and other pathway databases using the non-central hypergeometric test (Methods). The putative function of each module based on this enrichment analysis is summarized in the fourth column (see **Figs. 5**, **6**, **Supplementary Fig. 9**, and **Supplementary Table 4** for details). Two thirds of the modules have functions that correspond to core pathways underlying the respective traits, while the remaining modules correspond either to generic pathways that play a role in diverse traits or to pathways without an established connection to the considered trait or disease. Only pathways with a well-established link to the trait were considered core pathways. Generic pathways, such as cell-cycle-related or epigenetic pathways, were not considered core pathways because they are relevant for many traits and tissues, making them more difficult to target therapeutically. For example, modules 77 and 109 are both associated with schizophrenia and comprise pathways related to epigenetic gene silencing and nucleosome organization, respectively. Although there is evidence that epigenetic mechanisms may play a role in schizophrenia, we considered this to be a generic pathway.

a

Module 283: IgA nephropathy, rheumatoid arthritis
NF-kappa B signaling

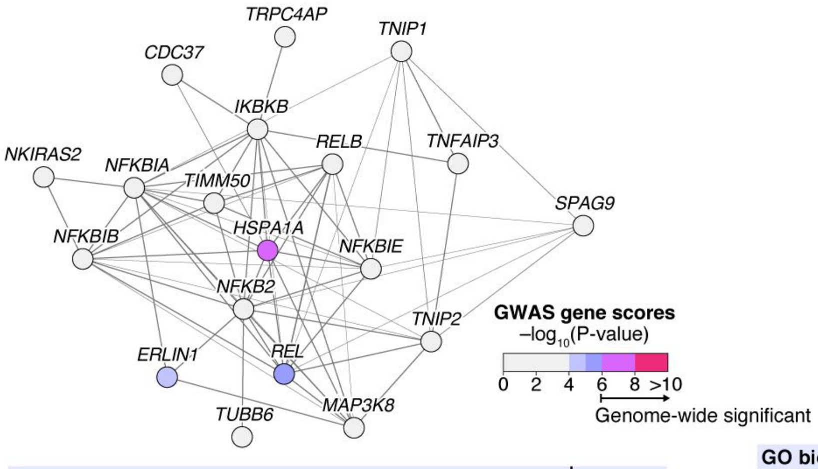

GO biological process	P-value
i-kappab kinase/nf-kappab signaling	1.4E-10
regulation of innate immune response	2.27E-08
positive regulation of nf-kappab transcription factor activity	3.44E-08
innate immune response-activating signal transduction	1.41E-06
stress-activated mapk cascade	1.44E-06
stress-activated protein kinase signaling cascade	1.62E-06
activation of innate immune response	1.65E-06
positive regulation of sequence-specific dna binding tf activity	1.73E-06
regulation of tumor necrosis factor-mediated signaling	2.92E-06
pattern recognition receptor signaling pathway	3.83E-06

Reactome pathways	P-value
rip mediated nfkb activation via dai	3.03E-08
traf6 mediated nfkb activation	3.94E-08
tak1 activates nfkb by phosphorylation and activation of ikks	7.65E-08
rig i mda5 mediated induction of ifn alpha beta pathways	1.4E-07
activation of nf kappab in b cells	6.95E-07
downstream signaling events of b cell receptor bcr	7.49E-06
il1 signaling	1.33E-05
signaling by the b cell receptor bcr	2.84E-05
nfkb and map kinases activation mediated by tlr4 signaling	3.67E-05
traf6 mediated induction of nfkb and map kinases upon tlr7 8 or 9 activation	3.99E-05

Mouse mutant phenotypes	P-value
decreased interleukin-6 secretion	7.14E-06
decreased b cell proliferation	2.86E-05
abnormal cytokine secretion	3.4E-05
small peyer's patches	3.92E-05
increased granulocyte number	4.48E-05
abnormal humoral immune response	5.66E-05
decreased igg1 level	6.25E-05
abnormal tumor necrosis factor secretion	9.45E-05
increased igm level	9.5E-05
increased b cell proliferation	0.00013

b

Module 203: IgA nephropathy
Complement and coagulation cascades

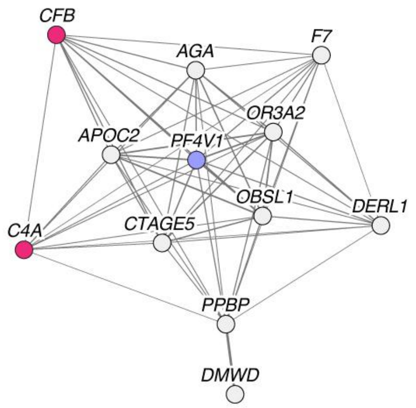

GO biological process	P-value
regulation of complement activation	4.62E-07
regulation of humoral immune response	1.42E-06
regulation of acute inflammatory response	3.43E-06
complement activation	3.91E-06
regulation of protein processing	5.04E-06
positive regulation of leukocyte chemotaxis	4.07E-05
positive regulation of apoptotic cell clearance	5.11E-05
regulation of apoptotic cell clearance	5.25E-05
complement activation, alternative pathway	1E-04
positive regulation of leukocyte migration	1E-04

Reactome pathways	P-value
regulation of complement cascade	6.14E-06
complement cascade	8.49E-05
formation of fibrin clot clotting cascade	0.00158
initial triggering of complement	0.00187
innate immune system	0.00486
olfactory signaling pathway	0.0148
gamma carboxylation transport and amino terminal cleavage of proteins	0.0155
common pathway	0.0215
bmal1 clock npas2 activates circadian expression	0.0277
ptm gamma carboxylation hypusine formation and arylsulfatase activation	0.0318

Mouse mutant phenotypes	P-value
abnormal blood coagulation	3.2E-05
glomerulonephritis	0.00014
decreased vascular permeability	0.00015
increased spleen germinal center number	0.00029
decreased spleen germinal center size	0.0003
increased anti-single stranded dna antibody level	0.00043
abnormal spleen germinal center morphology	0.00044
decreased spleen germinal center number	0.00049
cortical renal glomerulopathies	0.00054
abnormal circulating protein level	0.00089

Supplementary Figure 13

Modules Associated with IgA Nephropathy.

The top ten enriched GO biological processes, Reactome pathways and mouse mutant phenotypes are shown for two IgA nephropathy (IgAN) associated modules. P-values were computed using the non-central hypergeometric distribution (Methods).

(a) IgAN-associated module identified using the consensus analysis in the InWeb protein-protein interaction network (n = 19 genes). The module comprises immune-related NF-κB signaling pathways. Enriched mouse mutant phenotypes for module gene homologs include perturbed immunoglobulin levels (IgM and IgG1). The module implicates in particular the NF-κB subunit *REL* as a candidate gene. The *REL* locus does not reach genome-wide significance in current GWASs for IgAN but is known to be associated with other immune disorders such as rheumatoid arthritis.

(b) IgAN-associated module identified by the best-performing method (K1) in the InWeb protein-protein interaction network (n = 12 genes). Besides finding complement factors that are known to play a role in the disease (*CFB* and *C4A*), the module implicates novel candidate genes such as the chemokine *Platelet Factor 4 Variant 1* (*PF4V1*) from a sub-threshold locus, and is enriched for coagulation cascade, a process known to be involved in kidney disease[62]. The top two enriched mouse mutant phenotypes are precisely "abnormal blood coagulation" and "glomerulonephritis".

62. Madhusudhan, T., Kerlin, B. A. & Isermann, B. The emerging role of coagulation proteases in kidney disease. *Nat. Rev. Nephrol.* **12**, 94–109 (2016).

Supplementary Table 1 *(Included in the online Supplementary Information as Excel file)*

Collection of GWAS Datasets used for the Challenge.

The table lists the GWAS datasets used for the module scoring. The first column indicates whether the GWAS was used during the "leaderboard" or "final" evaluation phase. The five GWAS listed in the end ("extra") were not used for the scoring as they were added to the collection after the challenge. The PASCAL gene scores for all GWAS are available for download from the challenge website (file names are given in the last column). The original GWAS SNP summary statistics can be downloaded individually from the indicated sources or we can share the complete collection upon request.

Supplementary Table 2. Module identification methods

ID[a]	Description	Score[b]	Pre- / post-processing[c]
**Kernel clustering:** (i) the weighted adjacency matrix is transformed into a gene similarity matrix; (ii) a clustering algorithm is applied.			
K1	(i) Diffusion State Distance metric[24]; (ii) spectral clustering.	60	R
K2	(i) Singular Value Thresholding[63] maps the graph into a latent feature space; (ii) hierarchical clustering using Ward's method.	48	W, R
K3	(i) Large-scale Information Network Embedding (LINE)[48]; (ii) K-means clustering.	46	-
K4	(i) Extension of Spectral Clustering On Ratios-of-Eigenvectors (SCORE)[64] allowing for weighted networks and hierarchical structure of submodules; (ii) spectral clustering.	42	R
K5	(i) SCORE[64]; (ii) spectral clustering.	38	-
K6	(i) Diffusion kernel is applied to graph Laplacian[47]; (ii) Weighted Gene Coexpression Network Analysis (WGCNA)[7].	30	M
**Modularity optimization:** search algorithms are employed to find modules that maximize a modularity quality function.			
M1	Modularity optimization algorithms are extended with a multiresolution technique[27].	60	S, R
M2	Louvain community detection algorithm[49].	56	S,W,R,M
M3	Extension of a multi-network module identification method[31,36], here applied to single-layer networks.	48	R
M4	PageRank algorithm is used to create an initial partition for the Louvain method[75].	44	W, R
M5	Hierarchical module tree generated using Louvain algorithm, optimal partitions selected using modularity, conductance, and connectivity metrics[74].	42	W,R,M,F
M6	Greedy agglomerative clustering approach optimizes a score based on total weight of intra-module edges and module size.	40	S,W, M
M7	Fast greedy clustering algorithm[65] that iteratively divides modules to optimize the modularity.	40	-
M8	Modularity optimization by Conformational Space Annealing (Mod-CSA)[66] using the weighted adjacency matrix.	38	S, R
M9	Louvain algorithm is used for optimization of a generalized modularity metric with a resolution parameter.	37	R
M10	Louvain algorithm.	33	R
**Random-walk-based:** modules are identified using diffusion processes over the network.			
R1	Multi-level Markov clustering is extended with a regularization matrix to balance module sizes[28].	58	S, W, R
R2	Walktrap algorithm[50], output modules are filtered based on the median node degree.	44	S, R
R3	Walktrap algorithm.	43	S, R
R4	A machine learning approach for predicting disease genes from graph features is combined with the Infomap algorithm[51].	40	S,R,F
R5	Walktrap algorithm with varying number of steps.	39	S, F, M
R6	Infomap algorithm, Markov-time parameter is optimized to yield maximum number of modules of valid size.	38	R,M
R7	Markov clustering, output modules are filtered based on conductance and module size.	36	S, w
R8	Recursive local graph sparsification and clustering using Infomap for scalable community detection[73].	36	S, R
R9	Walktrap is used for the first network, Infomap for the remaining networks.	28	R
R10	Modules detected using Walktrap and Infomap are combined.	20	S
**Local methods:** agglomerative algorithms that grow modules from seed nodes.			
L1	Topological overlap matrix is clustered using the fast agglomerative SPICi[52] and SCAN++ algorithms[67].	55	S, W,R
L2	Basic agglomerative approach assigning genes to connected modules until the module size limit is reached.	31	W,R,M
L3	Local method that grows modules from seed nodes using a novel Triangle based Community Expansion (TCE) method.	30	M
**Ensemble clustering:** alternative clusterings sampled either from stochastic runs or from a set of different methods are merged.			
E1	Various clustering methods are applied on network embeddings created using DeepWalk[68], consensus modules are obtained using a bagging method.	46	S,W,M
E2	Consensus modules are derived from two flat clustering algorithms: ClusterOne and Finding Low-Conductance set with Dense interactions (FLCD)[69].	41	S,W,F
E3	Ensemble approach applied to integrate multiple Markov clustering runs.	24	S,R
**Hybrid methods:** different clustering methods are selected for each network based on leaderboard performance or structural quality scores.			
H1	Either Louvain, Infomap, or a continuous optimization method[70] are selected for each network.	50	R, F
H2	Either Louvain, Infomap, SPICi, or DCut[71] are selected for each network.	50	W,R
H3	Five different methods are applied to cluster networks, followed by filtering of modules based on structural quality metrics.	40	W,R, M, F
H4	Nine different methods are applied in different combinations, followed by module filtering and post-processing steps.	37	R,M,F
H5	Seven different methods are applied including an ensemble approach, followed by filtering and post-processing steps[72].	31	S,W,R,M,F
H6	WGCNA followed by fast greedy community detection to refine modules.	19	R
H7	No detailed description provided.	14	-
**Others**			
O1	Agglomerative algorithm that joins clusters based on the number of shared neighbors and the cluster sizes.	36	W,F
O2	Two-way modules (dense bipartite subgraphs) are mined using a heuristic algorithm.	33	W,F
O3	No detailed description provided.	12	-

(legend on next page)

Supplementary Table 2 *(See previous page)*

The 42 module identification methods applied in Sub-challenge 1 grouped by category.

[a]Identifier (ID) of the method used throughout the paper.

[b]Overall score of the method as defined in **Fig. 2b**.

[c]Common pre- and post-processing steps. Pre-processing steps are coded as: (S) sparsification of networks and (W) rescaling of edge weights. Post-processing steps are coded as: (R) recursive break-down of large modules, (M) merging modules of invalid size followed by re-modularization, and (F) filtering modules according to a quality metric.

Supplementary Table 3 *(Included in the online Supplementary Information as Excel file)*

Challenge scores of methods in the leaderboard and final round.

The table shows the challenge scores of all methods both for the leaderboard and final rounds.

Supplementary Table 4 *(Included in the online Supplementary Information as Excel file)*

Functional Enrichment of Consensus Trait Modules.

For each of the 21 consensus trait-modules shown in **Supplementary Fig. 12**, all categories with a Bonferroni-corrected P-value below 0.05 are listed (Methods). Only results for mouse mutant phenotypes, Reactome pathways and GO biological process annotations are included for brevity. Full results including all tested pathway databases and all challenge modules are available on the challenge website.

References

60. Huang, J. K. *et al.* Systematic Evaluation of Molecular Networks for Discovery of Disease Genes. *Cell Syst.* **6**, 484-495.e5 (2018).
61. de Lange, K. M. *et al.* Genome-wide association study implicates immune activation of multiple integrin genes in inflammatory bowel disease. *Nat. Genet.* **49**, 256–261 (2017).
62. Madhusudhan, T., Kerlin, B. A. & Isermann, B. The emerging role of coagulation proteases in kidney disease. *Nat. Rev. Nephrol.* **12**, 94–109 (2016).
63. Cai, J., Candès, E. & Shen, Z. A Singular Value Thresholding Algorithm for Matrix Completion. *SIAM J. Optim.* **20**, 1956–1982 (2010).
64. Jin, J. Fast community detection by SCORE. *Ann. Stat.* **43**, 57–89 (2015).
65. Clauset, A., Moore, C. & Newman, M. E. J. Hierarchical structure and the prediction of missing links in networks. *Nature* **453**, 98–101 (2008).
66. Lee, J., Gross, S. & Lee, J. Mod-CSA: Modularity optimization by conformational space annealing. *Phys Rev E* **85**, (2012).
67. Shiokawa, H., Fujiwara, Y. & Onizuka, M. SCAN++: Efficient Algorithm for Finding Clusters, Hubs and Outliers on Large-scale Graphs. *Proc VLDB Endow* **8**, 1178–1189 (2015).
68. Perozzi, B., Al-Rfou, R. & Skiena, S. DeepWalk: Online Learning of Social Representations. *ArXiv14036652 Cs* 701–710 (2014). doi:10.1145/2623330.2623732
69. Wang, Y. & Qian, X. Finding low-conductance sets with dense interactions (FLCD) for better protein complex prediction. *BMC Syst. Biol.* **11**, (2017).
70. Li, D., He, S., Pan, Z. & Hu, G. Active modules for multilayer weighted gene co-expression networks: a continuous optimization approach. *bioRxiv* 056952 (2016). doi:10.1101/056952
71. Shao, J., Yang, Q., Liu, J. & Kramer, S. Graph Clustering with Density-Cut. *ArXiv160600950 Phys.* (2016).
72. Tripathi, B., Parthasarathy, S., Sinha, H., Raman, K. & Ravindran, B. Adapting Community Detection Algorithms for Disease Module Identification in Heterogeneous Biological Networks. *Front. Genet.* **10**, (2019).
73. Banf, M. Network Module Detection using Recursive Local Graph Sparsification and Clustering. (2018). doi:10.20944/preprints201808.0421.v1
74. Mall, R., Ullah, E., Kunji, K., Ceccarelli, M. & Bensmail, H. An unsupervised disease module identification technique in biological networks using novel quality metric based on connectivity, conductance and modularity. *F1000Res* **7**, 378 (2018).
75. Perrin, D. & Zuccon, G. Recursive module extraction using Louvain and PageRank. *F1000Res* **7**, 1286 (2018).
