# Supplementary material for: Assessment of network module identification across complex diseases
Source: Nat Methods. 2019 Aug 30;16(9):843–52. doi: 10.1038/s41592-019-0509-5 (PMC6719725; doi:10.1038/s41592-019-0509-5)
Supplement: Supplementary file 15 — Reporting Summary [file 41592_2019_509_MOESM2_ESM.pdf]

nature research

Corresponding author(s): Daniel Marbach
Last updated by author(s): Apr 2, 2019

Reporting Summary

Nature Research wishes to improve the reproducibility of the work that we publish. This form provides structure for consistency and transparency in reporting. For further information on Nature Research policies, see Authors & Referees and the Editorial Policy Checklist.

Statistics

For all statistical analyses, confirm that the following items are present in the figure legend, table legend, main text, or Methods section.

n/a	Confirmed	
☐	☒	The exact sample size (n) for each experimental group/condition, given as a discrete number and unit of measurement
☐	☒	A statement on whether measurements were taken from distinct samples or whether the same sample was measured repeatedly
☐	☒	The statistical test(s) used AND whether they are one- or two-sided<br>*Only common tests should be described solely by name; describe more complex techniques in the Methods section.*
☐	☒	A description of all covariates tested
☐	☒	A description of any assumptions or corrections, such as tests of normality and adjustment for multiple comparisons
☐	☒	A full description of the statistical parameters including central tendency (e.g. means) or other basic estimates (e.g. regression coefficient) AND variation (e.g. standard deviation) or associated estimates of uncertainty (e.g. confidence intervals)
☐	☒	For null hypothesis testing, the test statistic (e.g. F, t, r) with confidence intervals, effect sizes, degrees of freedom and P value noted<br>*Give P values as exact values whenever suitable.*
☐	☒	For Bayesian analysis, information on the choice of priors and Markov chain Monte Carlo settings
☐	☒	For hierarchical and complex designs, identification of the appropriate level for tests and full reporting of outcomes
☐	☒	Estimates of effect sizes (e.g. Cohen's d, Pearson's r), indicating how they were calculated

Our web collection on statistics for biologists contains articles on many of the points above.

Software and code

Policy information about availability of computer code

Data collection	Does not apply / no software used for data collection
Data analysis	All code used in the challenge is freely available and open source. The Pascal tool was used for the challenge scoring, which is available from github. In addition, a snapshot of the Pascal version used for the challenge, scoring scripts, and code for module identification methods submitted by teams are available from the challenge website. Code used for functional enrichment analysis of modules is available from github.

For manuscripts utilizing custom algorithms or software that are central to the research but not yet described in published literature, software must be made available to editors/reviewers. We strongly encourage code deposition in a community repository (e.g. GitHub). See the Nature Research guidelines for submitting code & software for further information.

Data

Policy information about availability of data

All manuscripts must include a data availability statement. This statement should provide the following information, where applicable:

- Accession codes, unique identifiers, or web links for publicly available datasets
- A list of figures that have associated raw data
- A description of any restrictions on data availability

All challenge data and results are available from the challenge website (https://synapse.org/modulechallenge). This includes the challenge networks, module identification method descriptions and code provided by teams, the final module predictions of all teams for both sub-challenges, consensus module predictions for both sub-challenges, method scores at varying FDR cutoffs, individual module scores for all GWASs, enriched functional annotations for all modules, a snapshot of the PASCAL tool and scoring scripts, and the gene score p-values for the compendium of 180 GWASs used in the challenge (plus 5 additional GWASs obtained after the challenge). GWAS SNP p-values are available from the corresponding author (D.M.) upon request.

Field-specific reporting

Please select the one below that is the best fit for your research. If you are not sure, read the appropriate sections before making your selection.

☒ Life sciences ☐ Behavioural & social sciences ☐ Ecological, evolutionary & environmental sciences

For a reference copy of the document with all sections, see nature.com/documents/nr-reporting-summary-flat.pdf

Life sciences study design

All studies must disclose on these points even when the disclosure is negative.

Sample size	Considering the DREAM Challenge a scientific experiment, the number of samples corresponds to the number of final team submissions (Subchallenge 1: N=42; Subchallenge 2: N=33). Since the challenge was open for anyone to participate, the number of participating teams could not be determined in advance.
Data exclusions	No data were excluded.
Replication	Measures taken to verify reproducibility included the scoring of challenge submissions: (1) on a blinded test set, (2) at varying FDR cutoffs and (3) on subsampling of the test set (robustness analysis). The top-performing method scored best at each of these metrics, while performance of other teams varied.
Randomization	Does not apply, there were no experiments performed that could have been randomized.
Blinding	Challenge participants were blinded to the test set used for the scoring.

Reporting for specific materials, systems and methods

We require information from authors about some types of materials, experimental systems and methods used in many studies. Here, indicate whether each material, system or method listed is relevant to your study. If you are not sure if a list item applies to your research, read the appropriate section before selecting a response.

Materials & experimental systems

n/a	Involved in the study	
☒	☐	Antibodies
☒	☐	Eukaryotic cell lines
☒	☐	Palaeontology
☒	☐	Animals and other organisms
☒	☐	Human research participants
☒	☐	Clinical data

Methods

n/a	Involved in the study	
☒	☐	ChIP-seq
☒	☐	Flow cytometry
☒	☐	MRI-based neuroimaging
